# Structural and immunogenetic signatures guide CD4-mimetic HIV vaccine development

**DOI:** 10.1016/j.celrep.2026.117180

**Published:** 2026-04-15

**Authors:** Daniel L.V. Bader, Claudia T. Flynn, Oleksandr Kalyuzhniy, Gabriel Ozorowski, Martin M. Corcoran, Alison Burns, Pilar Altman, Romy Rouzeau, Troy Sincomb, Alessia Liguori, Monica L. Fernández-Quintero, Johannes R. Loeffler, Jonathan L. Torres, Hannah L. Turner, Erik Georgeson, Amelia Zhou, Hannah Voic, Sue Goo, Lara Shahin, Iszac Burton, Mengyu Wu, Robyn L. Stanfield, Saman Eskandarzadeh, Danny Lu, Nushin Alavi, Nicole Phelps, Ryan Tingle, Katherine McKenney, John Youhanna, Sonya Amirzehni, Torben Schiffner, Jon M. Steichen, Dennis R. Burton, Ian A. Wilson, Gunilla B. Karlsson Hedestam, Elise Landais, Jeong Hyun Lee, Devin Sok, Christopher A. Cottrell, Andrew B. Ward, William R. Schief

**Affiliations:** 1 Department of Immunology and Microbiology, The Scripps Research Institute, La Jolla, CA 92037, USA; 2 IAVI Neutralizing Antibody Center, The Scripps Research Institute, La Jolla, CA 92037, USA; 3 Consortium for HIV/AIDS Vaccine Development (CHAVD), The Scripps Research Institute, La Jolla, CA 92037, USA; 4 Department of Integrative Structural and Computational Biology, The Scripps Research Institute, La Jolla, CA 92037, USA; 5 Department of Microbiology, Tumor and Cell Biology, Karolinska Institutet, 17177 Stockholm, Sweden; 6 The Ragon Institute of Massachusetts General Hospital, Massachusetts Institute of Technology and Harvard University, Cambridge, MA 02139, USA; 7 The Skaggs Institute for Chemical Biology, The Scripps Research Institute, La Jolla, CA 92037, USA; 8 Moderna, Inc., Cambridge, MA 02142, USA; 9 Lead contact

## Abstract

HIV vaccine strategies include aims to elicit broadly neutralizing antibodies (bnAbs) targeting the CD4-binding site, that are derived from immunoglobulin heavy-chain variable genes 1–2 (V_H_1–2) and 1–46 (V_H_1–46). Here, we present an integrated analysis of V_H_1–46 bnAbs, including *in vitro* functional studies, cryo-electron microscopy structures of two V_H_1–46 bnAbs (1–23 and 9–71) complexed with envelope trimers, and comprehensive structural and immunogenetic analyses, to help guide vaccine design. We show that V_H_1–46-derived bnAbs use diverse light-chain variable (V_K_/V_L_) genes and LCDR3 lengths commonly found in human antibody repertoires, which generate unique LCDR3 signatures that influence both the antibody paratope and approach angle. We identify three V_H_1–46 bnAb classes, 1B2530 (V_L_1–47), CH235 (V_K_3–15), and 561 (V_K_3–20), with the 561 class further subdivided into types I and II. Our findings indicate that V_H_1–46 priming immunogens should be tailored to each bnAb class, with 561-class bnAbs presenting optimal targets for germline-targeting vaccine design.

## In brief

Identifying structural and immunogenetic features of bnAbs that determine neutralizing activity provides essential blueprints for HIV vaccine development. Bader et al. classify V_H_1–46 bnAb signatures, propose that V_H_1–46/V_K_3–20-derived bnAbs represent promising targets for germline-targeting vaccine design, and inform strategies for priming such humoral immune responses to the CD4bs.

## Graphical Abstract

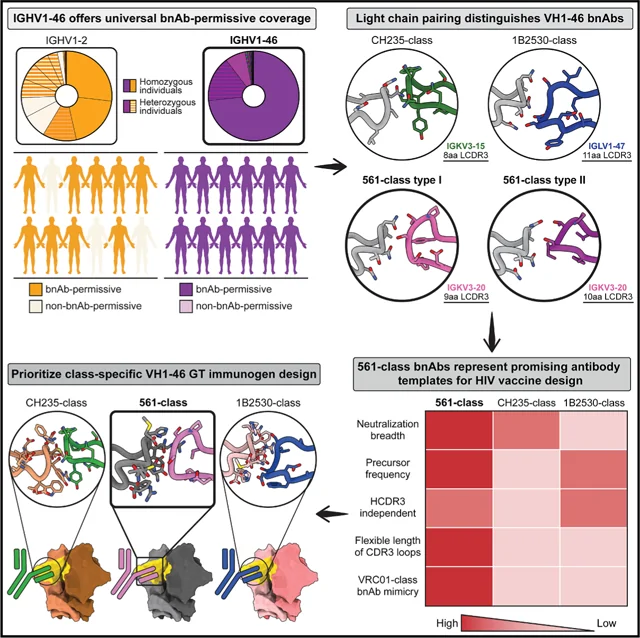

## INTRODUCTION

Despite decades of research, an effective, prophylactic HIV-1 vaccine has not yet been developed, mainly due to the extraordinary global diversity and immune-evading mechanisms of HIV-1.^1^ Antibody passive transfer studies in non-human primates (NHPs) have demonstrated that broadly neutralizing antibodies (bnAbs) can prevent infection, supporting antibody-based vaccine strategies.^2–7^ Antibody-mediated prevention (AMP) trials in humans further showed that the passively administered VRC01 bnAb, which is a V_H_1–2-encoded antibody that targets the CD4 binding site (CD4bs), effectively prevented infection but only for VRC01-sensitive viral strains with 80% inhibitory concentration (IC_80_) below 1 μg/mL in the TZM-bl *in vitro* neutralization assay.^8,9^ Although these findings strengthen the rationale for vaccine strategies aimed at eliciting protective bnAbs, most HIV-1 bnAbs fail to maintain broad neutralization coverage of contemporary isolates at this clinically relevant IC_80_ threshold, underscoring the importance of developing vaccination approaches that elicit highly potent bnAbs.^10^ Broad and potent neutralizing responses develop against sites of vulnerability on the HIV envelope protein (Env) in a small proportion of infected individuals,^11,12^ and the targeted induction of bnAb responses against these relatively conserved neutralizing epitopes has become central to HIV-1 vaccine development efforts.^13–15^ Each conserved supersite is recognized by bnAbs originating from diverse B cell ontogenies through distinct modes of recognition.^11,12,16–18^ However, exceptional breadth and potency remains restricted to specific supersites and precise molecular recognition.^11,12,16–18^ For instance, many CD4bs-targeting bnAbs achieve broad and potent HIV-1 neutralization by structural mimicry of CD4 receptor interactions with HIV-1 gp120.^18–26^ The essential role of CD4 in viral entry has resulted in relative conservation of the CD4bs across HIV-1 strains despite extensive variation in other areas of Env.^27–31^

CD4bs targeting by bnAbs is achieved by means of either heavy-chain variable (V_H_)-dominant^21,22,26,32,33^ or heavy-chain complementarity-determining region 3 (HCDR3)^24,34,35^ recognition, which have been termed “V_H_ binders” and “loop binders,” respectively.^18,23,24^ The V_H_-dominant mode of recognition is exemplified by members of the V_H_1 gene family, IGHV1–2, and IGHV1–46, which employ HCDR2 and heavy-chain framework region 3 (HFR3) to structurally emulate CD4 receptor contacts.^21,23–26,33,36^ V_H_1–2-derived antibodies are represented by VRC01-class bnAbs that utilize short 5-amino acid LCDR3 loops and diverse light-chain genes to engage the CD4bs with a conserved binding mode.^6,19,20,23,36–39^ V_H_1–46-restricted bnAbs demonstrate a similar diversity in their light chains, with representative examples including V_L_1–47 (1B2530 and 1NC9), V_K_3–15 (CH235.12), and V_K_3–20 (8ANC131, 8ANC134, 1–18, 9–89, 9–71, and 1–23).^21,25,26^ Unlike VRC01-class bnAbs, these bnAbs do not require relatively rare and short LCDR3 loops. Furthermore, the genetic and structural signatures of V_H_1–46 bnAbs remain less extensively characterized.^18,25,26^ VRC01-class and V_H_1–46-derived bnAbs, typified by N6 and 1–18, respectively, achieve remarkable neutralization breadth and potency, establishing them as important candidates for therapy and vaccine development.^26,33^ Despite the favorable phenotypes of V_H_-dominant CD4bs-specific bnAbs, their mode of recognition faces significant challenges. V_H_-restricted HIV neutralization is contingent on substantial somatic hypermutation (SHM) and specific binding geometries dictated by V_H_-gene and LCDR3 contacts and constrained by an extensive N-linked glycan shield that circumscribes the supersite.^24,31,40–42^ In particular, the N276 glycan restricts CD4bs accessibility and poses a major bottleneck for CD4bs bnAb elicitation by vaccination.^36,41–46^ To overcome this steric barrier, mature VRC01-class bnAbs have evolved specific adaptations: extensively mutated light chains with either amino acid deletions or glycine mutations within LCDR1 and key LFR3 mutations.^23,24,33,44,47^ N276 glycan accommodation is not restricted to LCDR1 modifications, as exemplified by VRC08, which retains wild-type HIV-1 neutralization capacity with germline-reverted light chains instead relying on an extended HCDR3 stabilized by intra-HCDR3 disulfide bonds to accommodate the N276 glycan.^44,48^ Furthermore, most of the VRC01-class responses primed by eOD-GT8 60-mer in the IAVI G001 trial exhibited the capacity to accommodate the N276 glycan on the engineered outer domain (eOD) mini-domain, though with reduced binding affinity compared to glycan-free variants, and structural analyses identified N276 accommodation mechanisms involving HCDR3 curvature or LCDR1 shift.^49^ Unlike well-characterized VRC01-class antibodies, the precise mechanisms by which V_H_1–46 bnAbs accommodate the N276 glycan, without LCDR1 deletions and with common-length LCDR3 loops, remain largely unexplored.

Germline-targeting vaccine design requires a priming immunogen to activate rare B cell precursors with defined germline-encoded features that confer the potential to bind a relatively conserved Env site with a pre-determined binding mode and ultimately develop broad neutralizing activity following a series of booster immunogens designed to select for the required SHM.^50–56^ Multiple germline-targeting priming immunogens engineered to activate VRC01-class bnAb precursors have shown success in pre-clinical^43,50,51,57-62^ and clinical trials.^55,63-66^ However, VRC01-class bnAbs are contingent on IGHV1–2*02 or *04 alleles, meaning individuals lacking both these alleles are unable to develop VRC01-class bnAbs through vaccination.^55,66–69^ In contrast, the IGHV1–46 gene displays minimal allelic diversity and, critically, the 100% sequence conservation within key paratope motifs across allelic variants indicates that all IGHV1–46 alleles are permissive for V_H_1–46-restricted bnAb development.^69^ Furthermore, to our knowledge, no IGVH1–46 gene deletions have been reported.^69^ Therefore, vaccine strategies that elicit V_H_1–46 bnAbs hold potential to achieve broader population coverage than strategies that elicit VRC01-class responses.^67,70^ Moreover, V_H_1–46-derived bnAbs are attractive targets because they recognize the CD4bs with LCDR3 lengths that occur at high frequencies in the human antibody repertoire.^26,71,72^ Efforts to elicit V_H_1–46-derived antibodies have advanced to clinical evaluation through a lineage-based vaccine approach (distinct from germline targeting) in which the priming immunogen was selected based on affinity for a specific unmutated common ancestor (UCA) antibody.^71,72^ The HVTN312 clinical trial (ClinicalTrials.gov: NCT06557785) is currently evaluating V_H_1–46 bnAb precursor priming and maturation in humans following vaccination with CH235-targeting immunogens.^73^ Previous studies have described the functional relevance of specific HC SHMs that are critical for development of CH235-like bnAbs.^25,74,75^ However, these studies do not address other classes of V_H_1–46 bnAbs. As a result, a comprehensive classification of structural and immunogenetic determinants that define V_H_1–46 bnAb classes remains lacking, limiting precise evaluation of vaccine-induced responses in pre-clinical and ongoing clinical trials. Moreover, a systematic classification of V_H_1–46 bnAbs may reveal promising alternative vaccine templates and guide the rational design of germline-targeting priming immunogens tailored to specific bnAb precursors with distinct genetic signatures.^36,50,54,76^ To address these knowledge gaps and evaluate V_H_1–46 bnAbs as vaccine templates, we employed an integrated approach combining *in vitro* functional studies, cryo-electron microscopy (cryo-EM), and comprehensive structural and immunogenetic analyses.

## RESULTS

### Global IGHV1–46 allelic conservation enables universal germline-targeting vaccine strategies

The prevalence of IGHV1–2 alleles non-permissive for VRC01-class bnAb development limits pan-population vaccine applications, particularly in African and Asian populations, where only 87% and 75% of individuals carry bnAb-permissive alleles, respectively, regions where prophylactic vaccines are most needed (Figures 1A, 1B, S1A, and S1B; Tables S1A–S1C).^69^ To assess whether V_H_1–46-restricted bnAbs offer advantages for alternative CD4bs-targeting vaccines, we analyzed IGHV1–46 allelic diversity, prevalence, and bnAb permissiveness using 1000 Genomes Project (1KGP) data.^69^ By analyzing the 1KGP data available from Corcoran et al.,^69^ we found that there are nine distinct IGHV1–46 alleles, four of which share 100% amino acid homology and none of which encode non-synonymous polymorphisms at key HCDR2 paratope positions, indicating that all alleles are bnAb permissive (Figures S1C–S1E). IGHV1–46 is predominantly represented by *01 and amino acid homologous alleles (*01_7985, *03, and *03_S3473), with most individuals carrying either *01/*01 or *01/*03 genotypes (Figures 1A, S1C, and S1D; Tables S1D and S1E). In contrast, IGHV1–2 displays higher allelic diversity with more distinct allelic combinations identified across populations (Figure 1A; Tables S1A–S1C). Thus, IGHV1–46 exhibits full bnAb-permissive coverage across the human population, whereas nearly 20% of individuals cannot mount VRC01-class V_H_1–2 bnAb responses (Figures 1A, 1B, and S1B; Table S1C). Importantly, all VRC01-class non-permissive individuals carry V_H_1–46 bnAb-permissive alleles, offering an alternative route to bnAb immunity (Figure S1F; Table S1C). These findings demonstrate that IGHV1–46 is universally represented by bnAb-permissive alleles across diverse human populations, making V_H_1–46-dependent bnAbs promising vaccine templates for global implementation.

### V_H_1–46/V_K_3–20 bnAbs potently neutralize contemporary HIV-1 isolates

Among V_H_1–46 bnAbs, we hypothesized that V_H_1–46 bnAbs utilizing V_K_3–20 light chains are attractive targets for HIV-1 vaccine design because they demonstrate broad and potent neutralization and utilize common immunogenetic features (Table S2).^21,26^ Contemporary HIV resistance to VRC01-class bnAbs observed in AMP trials prompted us to assess V_H_1–46/V_K_3–20-derived bnAb activity against current strains.^10^ We compared the neutralizing activity of four best-in-class V_H_1–46/V_K_3–20 bnAbs (1–18, 9–89, 9–71, and 1–23) with VRC01-class bnAbs, VRC01 and N6, against a contemporary 121-virus panel of HIV-1 strains isolated from infected patients in the AMP trials (Figure 1C; Table S3A).^8,10,26,77^ BnAbs 9–89 and 9–71 matched VRC01 in neutralization breadth while exhibiting greater potency, neutralizing >30% of AMP isolates at IC_80_ < 1 μg/mL (Figure 1C; Table S3A). BnAb 1–23 potently neutralized 50% of AMP isolates at IC_80_ < 1 μg/mL, approaching the breadth of best-in-class CD4bs-targeting bnAbs 1–18 and N6 (Figure 1C; Table S3A).^26,33^ This neutralization trend persisted in the conventional 111-virus multiclade Seaman panel, where bnAb 1–23 neutralized nearly 60% of global isolates at IC_80_ < 1 μg/mL (Figure 1D; Table S3B). Taken together, bnAbs 9–71 and 1–23 neutralized 38% and 55% of a 232-virus multiclade HIV-1 panel at IC_80_ < 1 μg/mL, respectively (Figures 1E and 1F; Table S3C). For comparison, bnAbs 1–18 and N6 potently neutralized 78% and 74% of global isolates, respectively (Figures 1E and 1F; Table S3C). Similar to VRC01-class bnAbs, 561-class bnAbs showed reduced potency and breadth against HIV-1 isolates from clades C and G and circulating recombinant forms (CRFs) strains (01_AE and 02_AG) (Figure 1G). When tested against AMP virus panels, 561-class bnAbs displayed higher breadth against clade B than clade C strains, mirroring N6, but demonstrated superior potency against clade C isolates (Figure S1G). VRC01 uniquely diverged from this pattern, showing reduced efficacy against AMP-derived clade B, F1, and BF1 isolates (Figure S1G). We confirmed that V_H_1–46/V_K_3–20-paired bnAbs demonstrate exceptional breadth and potency against both conventional and contemporary panels of HIV-1 isolates, offering promising features for HIV-1 vaccine design.

### V_H_1–46/V_K_3–20 bnAbs reveal subclass-specific glycan sensitivity profiles distinct from VRC01

As glycans within and around the CD4bs can restrict antibody neutralization potency and breadth, we next examined the interactions between CD4bs-proximal N-linked glycans and 561 bnAbs compared to VRC01. We generated seven potential N-linked glycosylation site (PNGS) knockout (KO) mutants across five genetically diverse HIV-1 strains and measured the hierarchy of glycan sensitivity by V_H_1–2- and V_H_1–46-derived bnAbs (Figures 1H and S1H; Table S3D). Consistent with previous observations for VRC01-class bnAbs, neutralization potency improved most dramatically against virions expressing Env lacking the N197, N276, N301, and N462 glycans (Figures 1I–1L and S1H; Table S3D).^41^ Compared to VRC01, V_H_1–46/V_K_3–20 bnAbs were less affected by steric constraints imposed by the N461/462 N-linked glycans but showed higher hindrance by N197 and N301 glycans (Figures 1I–1L). This pattern aligns with structural observations, as 1–18 approaches the CD4bs differently than VRC01 through a 6-amino acid (aa) HCDR1 insertion that enables binding closer to the adjacent protomer near N197 and N301 (Figures 1I–1L).^26^ In the absence of complex structures for other 561-subclass bnAbs, these distinct glycan sensitivity patterns suggest differential binding modalities for each group. Specifically, 9–89 is exclusively hindered by N197 and N301 glycans, consistent with an insertion in LFR3 that likely alleviates N276 glycan interference, whereas 9–71 and 1–23 bnAbs converge on a more canonical CD4bs bnAb-like glycan sensitivity profile (Figures 1H, 1J, and 1K). In summary, V_H_1–46/V_K_3–20 bnAbs show altered glycan sensitivity relative to VRC01 that varies across 561 bnAb subclasses and likely correlates with distinct binding orientations influenced by specific patterns of affinity maturation.

### V_H_1–46/V_K_3–20 bnAbs exhibit distinctive antibody footprints and molecular interactions

To enhance our structural understanding of V_H_1–46/V_K_3–20 bnAb recognition of the CD4bs, we determined cryo-EM structures of 9–71 and 1–23 in complex with BG505-MD39 Env at 3.5 and 3.1 Å resolution, respectively (Figures S2A–S2D). Both structures contained a secondary antibody fragment (Fab), RM19R, to minimize orientation bias during structure determination. Despite differences in SHM patterns and HCDR3 loop length, bnAbs 9–71 and 1–23 engage highly similar epitopes and show nearly identical V_H_ gene- and LCDR3-mediated contacts with gp120, with minor paratope differences in the HCDR3, LCDR1, and LFR3 motifs (Figures 2A–2C and S2C–2G; Table S2). Both antibodies share substantial epitope overlap with CD4, VRC01, and other V_H_1–46 bnAbs but display a unique footprint among CD4bs bnAbs (Figures 2A, 2B, and 2D). The footprint for V_H_1–46/V_K_3–20 bnAbs mimics CD4 receptor engagement, featuring a reduced buried surface area (BSA) at loop D and V5 regions but increased engagement of bridging sheets, CD4-BLP, inner domain (ID) and outer domain exit (ODe) loop (Figure 2E; Tables S6A and S6B). Consistent with this pattern, the binding footprint correlates with heightened sensitivity to N197 and N301 glycans near engaged regions and negligible sensitivity to V5 loop glycan N462 in areas of reduced contact (Figures 1F–1J, 2A, 2B, and 2E). Like other CD4bs V_H_-dominant binders, for V_H_1–46/V_K_3–20 bnAbs, the BSA within the paratope is focused on HCDR2 and HFR3 (Figures 2F and S3A; Table S6C). Notably, CH235.12 represents an exception with complete avoidance of N276 glycan contacts and dependence on HCDR3 for paratope BSA, demonstrating a hybrid neutralization strategy combining HCDR3 and V_H_ gene dependencies (Figures S3A and S3B; Table S6C).

BnAbs 1–23 and 9–71 mimic CD4- and V_H_1–2-derived bnAbs in CD4bs recognition by employing a germline-encoded R71_HC_-mediated salt bridge with D368_gp120_ (CD4-binding loop [CD4-BLP]), HCDR2 contacts with the CD4-BLP and ODe loop that vary in their Phe43 cavity-filling capacity, and somatic mutations in HFR3 that enable bridging sheet interactions (Figure S3C). However, V_H_1–46-derived bnAbs display a unique sequence motif at the HCDR2/HFR3 junction, a critical paratope region that exhibits the lowest sequence conservation between the two V_H_1 gene family members (Figures S3D-F). V_H_1–46 bnAbs exhibit two unique cavity-filling strategies. First, the S56R_HC_ mutation fills a pocket between loop D, β23/24 sheets, the ODe loop, and CD4-BLP (Figures 2G, 2H, S3F, and S3G). Second, except for CH235.12, the V_H_1–46 bnAbs preferentially use hydrophobic residues to fill the V5/D loop cavity, unlike V_H_1–2-derived bnAbs, which do not require SHM at N58 (Figures 2G, 2H, and S3F–S3H). While VRC01-class bnAbs engage the hypervariable V5 loop through a conserved Q61R_HC_ mutation, V_H_1–46-restricted bnAbs utilize a Q64R_HC_ mutation to bridge the β−23 sheet and the CD4-BLP via residues D457_gp120_ (99.4% conserved) and S365_gp120_ (86.9% conserved). Notably, N6, the broadest VRC01-class bnAb, also preferentially mutates residue 64 to an arginine (Figures 2G, 2H, and S3H).^33^ To conclude, V_H_1–46/V_K_3–20 bnAb CD4bs recognition displays V_H_-dependent characteristics while featuring a unique antibody footprint and convergent heavy-chain affinity maturation.

### Light-chain pairing dictates V_H_1–46 bnAb signatures and precursor abundance in human repertoires

For light-chain variable (V_K_/V_L_) genes, the highest sequence diversity is at the 3′ end, which VRC01-class bnAbs avoid through VJ recombination that produces short 5-residue LCDR3s, ensuring relative structural conservation independent of light-chain gene usage (Figures 3A, S4A, and S4B; Table S2).^24^ In contrast, IOMA-class and V_H_1–46-restricted bnAbs utilize longer LCDR3s that preserve gene-specific sequences yielding V_K_/V_L_-dependent paratope chemistries (Figures 3A–3G and S4A–S4E).^78^ Based on different V_K_/V_L_ gene usage, we classify V_H_1–46 bnAbs named after their prototype bnAbs: 1B2530-class (IGLV1–47), CH235-class (IGKV3–15), and 561-class (IGKV3–20) (Figure 3I). We propose that the 561-class, isolated from two distinct individuals, is further divided into two types: type I bnAbs with 9-residue LCDR3 loops, which include both 1–18-like and 8ANC-like bnAbs, and type II bnAbs with 10-residue LCDR3 loops, which include 1–23-like bnAbs (Figures 3F, 3G, 3I, and 3J).^21,26^ Compared to VRC01-class antibodies, V_H_1–46 bnAbs form more extensive hydrogen bonding networks with HIV-1 gp120 (Figure S4E). Additionally, 561-class bnAbs uniquely interact with loop D via conserved GSTP-rich LCDR3 motifs (Figures 3A and S4E). Independent of LCDR3 length, all 561-class bnAbs share a conserved T94/95_LC_ residue that forms hydrogen bonds with loop D N280_gp120_ (Figures 3A, 3F, and 3G). Similar to VRC01-class LCDR3s, CH235.12 utilizes aromatic (FYW) and hydrophilic (NDEQ) residues to form critical interactions with the CD4bs, exhibiting LCDR3-mediated BSA contributions that are twice that of the average 561-class antibodies, which engage gp120 with small polar residues (GST) (Figures 3A, S4D, and S4E).

Beyond their conserved LCDR3 motif, 561-class bnAbs utilize a conserved tyrosine at position 103–5_HC_ to hydrogen bond with highly conserved loop D residue K282_gp120_ (Figures 3H, S4G, and S4H). This is analogous to VRC01-class bnAbs, which use W103–5 to contact N/D279_gp120_ (Figures 3H and S4H).^46,55^ Convergent evolutionary solutions in LCDR3, HCDR2/HFR3, and HCDR3 resemble VRC01-class bnAbs, offering features for longitudinal immune monitoring during step-wise vaccine regimens (Figures 2H, 3A, 3F–3H, S3G, S3H, and S4H). Next, we assessed the putative frequency of 561-class bnAb precursors in the human antibody repertoire using a paired B cell receptor (BCR) database compiled from sequences obtained from four donors (STAR Methods). Notably, the estimated frequency of 561-class precursors was the highest among all CD4bs bnAb classes and was 23-fold higher than that of VRC01- and CH235-class precursors (Figures 3I and 3K). Our analysis highlights the pivotal role of V_K_/V_L_ gene usage in directing unique LCDR3-mediated interactions with gp120 and influencing ancestor B cell abundance in the human repertoire, particularly for V_H_1–46 bnAbs that utilize longer LCDR3 loops (Figures 3J and S4E). This immunogenetic distinction of V_H_1–46 bnAb classes guides vaccine template selection and establishes a framework to evaluate induction of V_H_1–46 antibody responses in pre-clinical and clinical trials (Figure S4M).

### 561-class type I and II bnAbs adopt distinct angles of approach

Antibody access to the CD4bs is sterically restricted by an adjacent protomer and a combination of N-glycans that impose approach angle constraints on CD4bs-directed antibodies (Figures 4A and 4B).^41,42,79^ Broad HIV-1 neutralization by CD4bs bnAbs is restricted to specific ranges of latitudinal and longitudinal angles, which define the bnAb heavy-chain orientation perpendicular to the viral membrane (latitude) and within the plane of the membrane (longitude) (Figures 4A, 4B, and S5A–S5J).^24^ Previous studies have shown that antibodies representing VRC01 subclasses engage the CD4bs with conserved approach geometries independent of light-chain pairing.^23^ To determine whether V_H_1–46-restricted bnAbs employ similar approach angles, we conducted a comparative analysis of 27 structurally characterized antibodies representing diverse B cell ontogenies and neutralizing capacities ranging from broad and potent to weakly neutralizing (Figure 4F and S5C–S5N; Table S7).^80^ VRC01-class bnAbs maintain fixed latitudinal orientations while allowing moderate longitudinal freedom (Figure 4D and S5G–S5J). In contrast to 561-class antibodies, CH235- and 1B2530-class bnAbs display angles of approach close to those of VRC01-class bnAbs (Figures 4D, 4E, and S5D–S5J). Among V_H_ binders, 561-class type I antibodies exhibit the highest longitudinal angles that more closely mimic CD4 engagement (Figure 4B, 4D, 4E, S5D, and S5J). 561-class type II antibodies display steeper latitudinal angles, resembling IOMA-like CD4bs recognition (Figure 4A, 4D, 4E, S5D, and S5G).^78^ Importantly, the approach geometry of 561-class bnAbs differs from other V_H_-dependent antibodies, which correlates with differential epitope recognition and N-linked glycan sensitivity phenotypes (Figures 1E–1I, 2E, 4B, 4D, and 4E; Table S6). Although 561-class type I and II bnAbs exhibit latitudinal variation, they maintain a conserved heavy- and light-chain (HC-LC) twist perpendicular to the membrane (Figures 4C–4F and S5N). This conserved binding mode is shared among V_H_-dominant bnAbs but contrasts with non-neutralizing, HCDR3-dominated, and N276g-dependent antibodies, which adopt wide-ranging HC-LC twist angles, constraining neutralization breadth (Figures 4C, 4F, and S5N). Overall, 561-class bnAbs engage the CD4bs more proximal to the neighboring protomer and display considerable latitudinal variation. These insights highlight how light-chain pairing and VJ recombination critically shape CD4bs-targeting antibody approach geometry, ultimately directing antibody footprints and affinity maturation.

### V_H_1–46 bnAbs overcome the N276 glycan barrier without light-chain mutations

The need for N276 glycan accommodation is arguably the principal impediment to VRC01-class bnAb induction, requiring reproducible elicitation of light-chain mutations to avoid inhibitory steric clashes (Figure 5A and S6E–S6G).^41–43,45,46^ On average, VRC01-class bnAbs cause a 60° latitudinal displacement of the N276 glycan to engage the CD4bs (Figures 5A–5D and S6A–S6D). Comparatively, IOMA-class and 1–18-like bnAbs displace the N276 glycan by 40° along the latitudinal plane (Figures 5A–5D and S6A–S6D). In contrast, N276 glycan-dependent antibodies, as for CD4 itself, cause very little displacement of the N276 glycan (Figures 5A–5D and S6A–S6D).^42^ Facilitated by steeper latitudinal approach angles, 561-class type II bnAbs induce a minor 12° displacement of the N276 glycan from its native conformation (Figures 5A–5D and S6A–S6D). Despite accommodating similar glycan orientations as N276 glycan-dependent bnAbs, 1–23 and 9–71 do not exhibit this glycan-dependent phenotype (Figures 1F, 5A–5D, and S6G). Longer LCDR3 loops increase the distance between the light-chain center of mass (COM_LC_) and N276_CA_, accommodating a binding mode with higher HC-LC latitudinal angles while minimizing N276 displacement (Figures S6K and S6L; Table S8). These structural insights support the hypothesis that 561-class type II bnAbs may be less susceptible to N276 glycan steric hindrance.

We then characterized light-chain requirements across CD4bs bnAb classes relevant to HIV-1 vaccine design (VRC01-, CH235-, and 561-class). We produced chimeric Abs with germline-reverted light chains (mHgL) for N6, VRC01, 1–23, 9–71, and CH235.12 bnAbs and evaluated their neutralization capacity against a 34-virus global HIV-1 panel (Figure 5E; Table S3D). VRC01 and N6 mHgL antibodies neutralized 6% and 9% of global HIV-1 isolates at an IC_50_ of <1 μg/mL, respectively (Figure 5E; Table S3E). In contrast, 1–23 and 9–71 mHgL potently neutralized 32% and 15% of viruses at the same threshold, respectively (Figure 5E; Table S3E). Remarkably, CH235.12 demonstrated negligible light chain requirements for neutralization activity, with both mature and mHgL mAbs potently neutralizing >80% of isolates (Figure 5E; Table S3E). This finding substantiates the unique ability of CH235.12 to flip the loop D conformation, avoiding N276 glycan contacts entirely, representing a conformational transition mediated by non-templated junctional HCDR3 motifs (Figures 5I, 5J, S6M, and S6N).^72,81^ The DH1285 antibody, elicited by a CH235-lineage priming immunogen in NHPs, also distorts loop D through HCDR3-mediated contacts (Figures S5M and S6O).^72^ DH1285 shares epitope specificity and structural signatures with 561-class bnAbs, including a GSTP-rich LCDR3 motif, but its binding mode is uncharacteristic of V_H_-dependent CD4bs bnAbs (Figure S6Q; Tables S6A, S6B, and S7). Thus, HCDR3-mediated loop D rearrangements are compatible with diverse LCDR3 motifs and accommodate distinct binding modes (Figures S6M–S6Q).

Binding affinity measurements corroborated the neutralization phenotype, whereby CH235.12 mHgL retained near-native binding and VRC01 lacking light-chain SHM exhibited substantially reduced geometric mean affinity against wild-type (WT) stabilized trimers (Figures 5F, 5G, and S6H; Table S4B). This affinity reduction was exclusively mediated by N276 glycan steric occlusion, as binding by all mHgL mAbs to the N276Q KO BG505 trimer remained largely unaffected (Figure 5H; Table S4B). Notably, light-chain mutations primarily contribute to binding by reducing the dissociation rate, suggesting that heavy-chain SHM alone is sufficient for initial CD4bs engagement but cannot maintain stable binding under glycan repulsion (Figures 5G and S6H; Table S4B). In conclusion, V_H_1–46 bnAbs exhibit diverse mechanisms to avoid N276-mediated steric clashes. Although germline-reverted CH235.12 displays remarkable neutralizing activity, this phenotype is HCDR3 dependent (Figures 5I, 5J, S6N, and S6O). In contrast, 561-class type II bnAbs display reduced light-chain requirements for HIV-1 neutralization with extended LCDR3 loops that retract the LC away from loop D (Figures 5K and 5L).

### CD4bs recognition by 561-class bnAbs is relatively independent of HCDR3

While germline-targeting vaccine design favors antibodies that rely on common V, D, and J genes rather than non-templated HCDR3 motifs, the critical role of HCDR3 in CH235.12 neutralizing activity prompted us to assess whether 561-class bnAbs share this dependency through three experimental approaches (Figures S7A–S7C).^24,32,34,42,44^ First, we generated two 1–23 mutants with HCDR3 lengths of 15 and 20 residues, representing the average length in the human antibody repertoire and the native 1–23 length, respectively. To avoid sequence-dependent biases leading to alternate loop conformations upon shortening the loop, we replaced the native sequence with a flexible (GGS)_n_ tandem repeat (Figures 6A and S7A). 20H3-GGS and 15H3-GGS 1–23 variants potently neutralized 78% of a Seaman panel-derived multiclade panel of 9 viruses (GeoMean IC_50_ of 0.087 μg/mL and 0.104 μg/mL), corresponding to a 2.8- and 3.4-fold reduction in neutralization potency (Figures 6B, 6C, and S7B; Table S3F). Both GGS-rich antibodies were capable of CD4bs recognition and displayed negligible reduction in GeoMean affinity against multiple WT Env trimers (Figures 6B, 6C, and S7B; Table S4C). Minimal effects on binding and neutralization activity imposed by the flexible Gly-rich motif and the 5-residue HCDR3 truncation underscore that 561-class bnAbs recognize the CD4bs in a V_H_-dependent manner.

Next, we selected 22 random V_H_1–46/V_K_3–20 BCRs from an antibody sequence database with paired heavy and lightchains (STAR Methods) and replaced the HCDR3 of 1–23 with the HCDR3s of the selected antibodies to generate chimeric 1–23 monoclonal immunoglobulin G (IgG) (Figure 6D and S7C–S7E). The selected BCRs display varied HCDR3 loop lengths and use diverse IGHD and IGHJ genes, simulating the natural diversity that originates from V(D)J recombination (Figures 6D and S7C–S7E). We found that 64% of HCDR3-chimeric 1–23 variants recognized WT stabilized trimers (Figures 6F, S7E, and S7G). These mAbs display a broad range of GeoMean K_D_ values from 122 nM to 3.5 μM, equating to 22- to 621-fold lower affinity compared to the 1–23 bnAb (Figures 6F, 6G, and S7E; Table S4C). Furthermore, 100% (8 of 8) of chimeric HCDR3 1–23 mAbs derived from IGHJ6^+^ BCRs exhibited measurable affinity against all WT stabilized trimers, consistent with the fact that all known V_H_1–46/V_K_3–20-derived bnAbs use IGHJ6 (Figures 6F and S7E). Therefore, although 561-class bnAbs may not strictly require IGHJ6, our data suggest that IGHJ6 usage greatly favors effective binding. Importantly, the geometric mean affinity for our HIV-1 Env panel directly corroborated neutralization capacity against a distinct panel of HIV-1 isolates across three engineered 1–23 mAbs (Figures 6G and S7E). 1–23 H3-Ab2 and H3-Ab3 mAbs potently neutralized 78% of HIV-1 isolates (GeoMean IC_50_ of 0.696 μg/mL and 0.938 μg/mL), while H3-Ab4 neutralized only 33% of viruses at 1.538 μg/mL GeoMean IC_50_ (Figures 6G and S7E; Table S4F). The capability of 1–23 variants with diverse and randomly selected HCDR3s to retain binding and neutralization activity further emphasizes the inherent V_H_ gene dependency.

Next, we evaluated the importance of Y103–5 in CD4bs recognition by site-saturation mutagenesis (SSM) (Figure 6H). All 20 single-residue 1–23 mutants bound a cocktail of WT Envs with low nanomolar geometric mean affinity (Figures 6H, 6I, and S7F; Table S4C). Notably, aromatic and hydrophobic residue replacement had negligible effects on affinity, while acidic residues and glycine mutations caused the greatest reductions in affinity (Figure 6I; Table S4C). This amino acid tolerance mirrors VRC01-class bnAbs that typically employ W103–5 but can tolerate alternative bulky and hydrophobic residues for optimal loop D contacts.^55,66^

Taken together, these findings show that the prevalence of Y103–5 and long HCDR3 loops observed in 561-class antibodies are merely by-products of V(D)J rearrangement with IGHJ6 rather than specific requirements for CD4bs recognition. Importantly, HIV-1 neutralization by 561-class bnAbs is primarily V_H_-gene dependent, an important finding to guide germline-targeting vaccine design.

### Minimally mutated 561-class bnAbs guide reductionist vaccine design

CD4bs-targeting antibodies are among the most mutated classes of bnAbs against HIV-1 (Figure S8A).^12^ To understand which mutations are more important for bnAb activity, we explored key structural determinants that influence HIV neutralization by 561-class bnAbs.^36,46,55^ First, we generated mAb 1–23 variants with individual and combined heavy-chain CDR/FR patches mutated from the germline, paired with mature and germline-reverted light chains (Figure S7B).^36^ No single mutated patch alone was sufficient for WT HIV-1 neutralization (Figure S8B; Table S3G). Heavy chains with combined mutated patches HCDR2/HFR3 (1–23 mH2-FR3) and HCDR1/HCDR2 (1–23 mH1-H2) paired with the mature light chain neutralized 80% and 50% of a 10-virus global tier 2 panel with a GeoMean IC_50_ of 0.21 and 0.50 μg/mL, respectively (Figure S8B; Table S3G). The 1–23 mH2-FR3 heavy chain paired with a germline-reverted light chain (gL) neutralized 40% of isolates, demonstrating that N276^+^ tier-2 neutralization can be achieved with key heavy-chain SHM alone (Figure S8B; Table S3G). Thus, 561-class bnAbs require both HCDR2 and HFR3 mutations for neutralizing activity, a signature commonly required by VRC01-class and other HIV bnAbs (Figure S8B; Table S3G).^36,82^

Building on these findings and following previous efforts to develop minimally mutated VRC01-class bnAbs, we employed structure-guided design to generate minimally mutated variants of 9–71 and 1–23 bnAbs (min1–23 and min9–71) that are 16% and 10% mutated in the heavy and light chains, respectively, similar to minVRC01 and min12A21 (Figures 7A–7C and S8C).^36,83^ min1–23 and min9–71 neutralized 39% and 37% of a 132-virus global AMP- and Seaman-derived panel with a GeoMean potency of 2.277 μg/mL and 2.134 μg/mL, respectively (Figure 7D; Table S3H). Against a multiclade 23-virus AMP panel, both min561-class antibodies display a neutralization breadth rivaling that of mature and minimally mutated bnAbs of interest for HIV-1 vaccine design (Figures S8E-S8G; Tables S3J-K). Notably, all minimally mutated bnAbs demonstrated a significant drop in neutralization potency except minVRC01, an exception that maintains effectiveness with an engineered key heavy-chain mutation (G54F) and deletions in LCDR1 (Figure 7D and S8D–S8F; Tables S3H-J).^36^ The reductions in potency upon reverting second-shell mutations highlight the critical role of such mutations in stabilizing the antigen-Ab interface and decreasing dissociation rates (*k*_off_) to improve overall binding affinity (K_D_) (Figures S8H and S8I; Table S4D). Faster *k*_off_ rates observed by minimally mutated antibodies correlated with N276 glycan steric hindrance, as evidenced by a modest 1.7-fold increase in dissociation rate when 1–23 minHgL binds to BG505 N276(−) trimer, compared to a 7-fold increase against WT BG505 (Figures S8H and S8I; Table S4D). We highlight that 1–23 minHgL neutralized 14% of a comprehensive panel of 132 WT viruses, which, to the best of our knowledge, is an unprecedented phenotype previously only observed with CD4bs-targeting bnAbs against N276A mutant viruses (Figure 7D; Table S3H).^36^ Collectively, these findings show that 561-class bnAbs require comparable heavy- and light-chain requirements as VRC01-class bnAbs and establish a plausible goal for reductionist vaccine design strategies.

## DISCUSSION

Whereas most HIV bnAbs exhibit HCDR3-dominant interactions with Env, the majority of CD4bs-targeting bnAbs exhibit V_H_-dominant recognition restricted to IGHV1–2 and IGHV1–46 genes, distinguished by similar binding modalities and substantial epitope overlap. Despite these shared features, V_H_1–2- and V_H_1–46-derived bnAbs demonstrate notable molecular diversity in epitope engagement, driven by distinctive SHM patterns and LCDR3 motifs. Here, we defined three distinct classes of V_H_1–46 bnAbs that recognize the CD4bs with specific LCDR3s determined by light-chain V gene usage. Our findings demonstrate that 561-class (V_H_1–46/V_K_3–20-paired) bnAbs represent promising germline-targeting vaccine candidates based on their neutralizing activity against contemporary isolates, common genetic elements (stable IGHV gene, V_H_/V_K_-pairing, LCDR3 lengths), higher precursor frequencies, flexible L- and H-CDR3 lengths, and convergent maturation signatures (Figures 1A, 1B, 2G, 2H, and 3I–3K).

We identify a hybrid HCDR3- and V_H_-dependent mechanism for CD4bs recognition that enables broad HIV neutralization without requiring light-chain SHM. Specifically, for CH235.12 and vaccine-elicited DH1285 antibodies, CD4bs binding involves a HCDR3-mediated conformational change of loop D that reorients the N276 glycan away from the light-chain interface, eliminating steric clashes altogether.^25,72^ This HCDR3-dependent phenotype offers a potentially expedited pathway to induce neutralizing responses that overcome the N276 glycan barrier using fewer boosting immunogens. However, despite these advantages, successful germline-targeting vaccine approaches require antibody targets that rely on genetically encoded structural features common in human repertoires, ensuring targetable motifs for germline-targeting (GT) immunogen design and increased likelihood of reproducible elicitation. HCDR3-dominant vaccine campaigns that have demonstrated pre-clinical success target specific D and/or J gene encoded structural motifs, enabling rational immunogen design that compensates for the typically lower precursor frequencies of HCDR3-dependent bnAbs.^54,56,76,84,85^ In contrast, antibody targets dependent on extensive non-templated HCDR3 residues generated by stochastic V(D)J recombination, such as CH235.12 and DH1285, present significant design and immune monitoring challenges, and whether current germline-targeting approaches can successfully extend to such targets remains to be determined.

From a vaccine elicitation standpoint, 561-class bnAbs have notable similarities to VRC01-class bnAbs but also potential advantages over VRC01-class bnAbs. 561-class bnAbs utilize key heavy-chain mutations and a canonical GSTP-rich LCDR3 with conserved T94/95_LC_ and P95/95a_LC_ residues, with the key threonine arising through SHM, which is reminiscent of conserved selection for E96_LC_ in VRC01-class bnAbs.^23,55,66^ In addition, HIV neutralization by minimally mutated 561- and VRC01-class antibodies demonstrates similar heavy-chain requirements and functional relevance. These structural parallels to VRC01-class bnAbs create traceable developmental signatures that enable longitudinal immune monitoring of vaccine-induced responses through conserved LCDR3 and heavy-chain affinity maturation patterns. The primary advantage of 561-class induction is the potential for broad bnAb precursor priming across the human population due to the apparent capacity of all IGHV1–46 alleles to permit interactions with priming immunogens, in contrast to the restriction to IGHV1–2 alleles *02 and *04 for VRC01-class precursor priming (Figures 1A, 1B, S1A, and S1B).^55,65,68,69^ Differences in neutralization specificities between 561-class and VRC01-class bnAbs, linked to differences in antibody footprint and angle of approach, indicate that inducing both types of bnAbs might offer a CD4bs response with optimal breadth. Future development and testing of full germline-targeting regimens to induce 561-class, VRC01-class, and combined responses should be explored.

Our analysis identifies critical considerations for the development of V_H_1–46-specific priming immunogens, underpinned by two distinguishing features: the intrinsic structural diversity between IGHV1–2 and IGHV1–46 genes and the distinct genetically prescribed LCDR3 signatures. IGHV1–2 and IGHV1–46 genes differ at the key HCDR2 paratope residues 50, 53, 54, 56, and 58 (Figures S3D–S3F). While IGHV1–46-derived bnAbs require SHM at these positions (I50, S53, G54, S56, and S58) to achieve optimal gp120 interactions, IGHV1–2*02/04-dependent bnAbs inherently possess favorable germline-encoded contact residues at four of those positions (W50, N53, S54, and N58). This distinction may give V_H_1–2-naive B cells a competitive advantage when encountering circulating HIV, likely explaining the greater prevalence of VRC01-class compared to V_H_1–46 bnAbs among people living with HIV. Consequently, effective V_H_1–46 GT immunogens must be engineered to compensate for the relative paucity of favorable germline-encoded interactions and preferentially engage V_H_1–46-naive B cells.

Whereas clinically validated VRC01-class priming immunogens (eOD-GT8 60-mer and GT1.1 trimer) engage diverse subclasses of VRC01-class precursors using different light-chain V genes, the structural divergence among V_H_1–46 bnAb classes utilizing unique V_K_/V_L_-prescribed LCDR3 features necessitates class-specific/light-chain V gene-specific GT immunogens.^55,66^ These findings suggest a strategic shift for V_H_1–46 bnAb induction with tailored GT immunogens that activate class-specific V_H_1–46 bnAb precursors with unique LCDR3s rather than widespread V_H_-germline engagement. Whether immunogen design can preferentially select 561-class type I versus type II precursors or whether certain 561-class subtypes possess inherent competitive advantages for HIV antigen recognition remain open questions requiring immunogen design efforts and *in vivo* pre-clinical evaluation. In summary, V_H_1–46/V_K_3–20-restricted bnAbs present an alternate pathway toward the induction of CD4bs-targeting bnAbs and provide key features that support reductionist germline-targeting vaccine design strategies.^50,54,56,76^

### Limitations of the study

While IGHV1–46 is globally prevalent across populations, we cannot exclude that some individuals not represented in the 1KGP dataset may carry IGHV1–46 variants that limit truly universal coverage by V_H_1–46-based vaccines. We classify V_H_1–46 bnAbs based on V_K_/V_L_ gene usage and LCDR3 length based on their binding and functional implications. Importantly, this merely represents our current best understanding of V_H_1–46-restricted CD4bs recognition, and we cannot exclude the possibility that 1B2530- and CH235-class antibodies with alternative LCDR3 lengths might achieve effective binding. Determining such possibilities would require directed evolution efforts beyond the scope of this study, and whether additional V_H_1–46 bnAbs with these features emerge from natural infection or vaccination remains to be observed. Thus, future studies may refine the proposed V_H_1–46 bnAb definitions. While V_H_1–46/V_K_3–20-pairing shows the highest representation in naive B cell repertoires, suggesting significant vaccine design promise, these bioinformatically computed frequencies likely overestimate true bnAb precursor numbers, since many BCRs will not generate optimal CDR3 loops for CD4bs engagement. Importantly, this limitation applies to all bnAb precursor frequency calculations, including those for previously established HIV bnAbs. Additionally, since current clinically tested priming immunogens (eOD-GT8 60-mer and GT1.1) preferentially engage VRC01-class bnAb precursors, developing novel immunogens that selectively target class-specific V_H_1–46 bnAb precursors represents an important future direction that lies beyond the scope of this study.

## RESOURCE AVAILABILITY

### Lead contact

Further information and requests for resources and reagents should be directed to lead contact, William R. Schief (schief@scripps.edu).

### Materials availability

Plasmids or recombinant proteins for HIV-1 stabilized trimers, antibodies, or SPR reagents used in this study are available upon request from the lead contact, William R. Schief (schief@scripps.edu), under a material transfer agreement with the Scripps Research Institute.

### Data and code availability

All data underlying this study are present in the paper or the supplemental information. Maps generated from the electron microscopy data have been deposited in the Electron Microscopy Databank (http://www.emdatabank.org/) under accession numbers EMD-71677 and EMD-71678. Atomic models corresponding to these maps have been deposited in the Protein Data Bank (http://www.rcsb.org/) under accession numbers 9PIT and 9PIV. BCR sequences used in this manuscript for selecting random HCDR3s are available in the public data repository https://github.com/SchiefLab/Bader2025 permanently archived https://zenodo.org/records/16915002. Any additional information required to analyze the data reported in this paper is available from the lead contact, William R. Schief (schief@scripps.edu), upon request.

## STAR★METHODS

### EXPERIMENTAL MODEL AND SUBJECT PARTICIPANT DETAILS

#### Cell lines

HEK-derived cells 293F and 293T were purchased from Thermo Fisher Scientific. 293F cells were used directly from the commercial sources following manufacturer suggestions and maintained in Gibco Freestyle 293 Expression Medium (Thermo Fisher) at 37°C in 7% CO_2_ with shaking at 120 rpm. TZM-bl cells are derived from a HeLa cell line that expresses the CD4 receptor, CXCR4 and CCR5 chemokine co-receptors, luciferase and β-galactosidase genes under the control of the HIV-1 promoter. Both 239T and TZM-bl cell lines were maintained at 37°C in 5% CO_2_ in Dulbecco’s Modified Eagle Medium, 2 mM L-glutamine, 10% fetal bovine serum (FBS), and 1x Penicillin-Streptomycin. Cells were not tested for mycoplasma contamination. Sex of both cell lines was female. Cell lines have not been authenticated.

### METHOD DETAILS

#### HIV-1 Env construct design

A panel of native-like antigens were derived from diverse HIV-1 isolates with wild-type CD4-binding sites to screen mature CD4bs bnAbs and engineered variants, and changes in binding breadth and strength were measured as indicators of potential effects on neutralization activity. We selected five strains whose soluble antigens were expressed in high yields and formed stable pre-fusion Env trimers: BG505 (clade A), CNE40 (clade CRF07_BC), DU422–1 (clade C), 001428 (clade C), and 3000–090-pblib002 (clade C). All HIV-1 Envs were expressed as gp140 recombinant ectodomains truncated at residue 664^102–104^ and contained a His-tag (HHHHHH) sequence at the C terminus of gp41 after residue D664 for purification by affinity chromatography. These trimers (BG505, CNE40, DU422–1 and 001428) were stabilized using the SOSIP-MD39 platform, which includes the original SOSIP mutations (501C, 605C, I559P)^30,105,106^ plus additional MD39 mutations that typically further improve antigenic profile, yield, and thermal stability: 106E, 271I, 288L, 304V, 319Y, 519S, 561P, 568D, 570H, 585H, N363Q.^96^ Homogeneity and expression yields of the 3000–090-pblib002 trimer were optimized by incorporating three sets of stabilizing mutations: established HIV-1 Env mutations (204I, 535N, 538F, 548F, 556P, 573F, 588E, 589V, 647F, 651F, 655I, 658V),^107,108^ two glycine mutations in gp41 (T569G, N636G),^109^ and additional V3 loop stabilizing mutations (R304V and T319V). The furin cleavage site of CNE40, DU422–1, 001428, and 3000 trimers was replaced by the flexible furin cleavage-independent linker (link 14).^54^ Three N-linked glycan knockout trimers (N197Q, N276Q, and N462Q) were generated as single point mutants of BG505 MD39 and expressed with C-terminal His-tags. All five His-tagged WT stabilized trimers served as analytes for affinity measurements, and BG505 MD39 SOSIP was selected for cryo-EM complex structures.

#### Protein expression and purification

His-tagged stabilized trimers were produced as previously described.^46^ Briefly, soluble HIV-1 Envs were obtained by transient transfection of HEK-293F cells (ThermoFisher) and purified by immobilized metal ion affinity chromatography (IMAC) using 5 mL HisTrap columns (Cytiva) followed by size-exclusion chromatography (SEC) using Superdex 200 Increase 10/300 GL columns (Cytiva). The molecular weight and homogeneity of stabilized trimers was confirmed by size-exclusion chromatography-multi-angle light scattering (SEC-MALS) in PBS pH 7.4 using Superdex 200 Increase 100/300 GL columns (Cytiva) with an isocratic flow of 0.5 mL/min followed by DAWN HELEOS II and Optilab T-rEX detectors (Wyatt Technology).

Genes encoding the antibody variable fragment (Fv) regions were synthesized by GenScript and cloned into antibody expression vectors pCW-CHIg-hG1 and pCW-CLIg-hk for heavy and light chain genes, respectively. Monoclonal antibodies (mAbs) were produced and expressed as previously described.^46,80^

#### Cryo-EM sample preparation and data collection

BG505 MD39-RM19R complexes with 1–23 and 9–71 were formed by incubating 500 μg of the trimer with either Fab at a 3:1 molar ratio in Tris-buffered saline (TBS) buffer at 20°C overnight. The Env base-binding RM19R Fab was added to improve tumbling and orientation bias. The complexes were purified from unbound Fabs and antigen by size-exclusion chromatography (SEC) using a HiLoad Superdex 200 16/600 column (Cytiva) equilibrated with TBS buffer. Fractions containing the HIV-1 Env-Fab complexes were pooled and concentrated to approximately 5.8 mg/mL with a 100 kDa MWCO Amicon Ultra centrifugal filter (Millipore Sigma). 3.5 μL of Env-Fab complexes were mixed with 0.5 μL of 0.04 mM lauryl maltose neopentyl glycol (LMNG) and applied to a UltrAuFoil holey gold R1.2/1.3 300 mesh grids (Quantifoil Micro Tools GmbH), which had been glow discharged for 15 s in a PELCO easiGlow Glow Discharge Cleaning System (Ted Pella, Inc.). The grid was blotted and plunged into liquid ethane using a Vitrobot Mark IV (Thermo). Cryo-EM data were collected on a 300 keV FEI Titan Krios electron microscope (ThermoFisher) equipped with Gatan K2 Summit direct electron detector operating in counting mode and a Gatan GIF BioQuantum energy filter with slit with of 10 eV. Images were collected at a nominal magnification of ×130,000×, resulting in a pixel size of 1.045 Å. A defocus range of −0.8 to −1.6 μm and −0.8 to −1.4 μm was applied during data collection of 1–23 and 9–71 samples, respectively. The dose was fractionated over 35 frames collected at a total dose of 50 e^−^Å^−2^, that were aligned and dose-weighted using MotionCor2.^110^ Automated data collection was performed using the Leginon software suite.^100^ Data collection and processing parameters are reported in Table S4.

#### Single-particle cryo-EM data processing

Data processing was performed in CryoSPARC (v4.7).^101^ Initial 2D templates were obtained using blob picker, particle extraction and 2D classification jobs. Select 2D templates were used to extract particles using template picker and particle extraction jobs with a particle diameter cutoff of 250 Å and with a box size of 300 pixels, respectively. After one round of 2D classification, 2D classes with well resolved density for base-binder (RM19R) and CD4bs-targeting (9–71 and 1–23) Fabs were selected to generate two distinct 3D ab-initio reconstructions. The best 3D volume was refined by heterogeneous refinement followed by homogeneous refinement with C3 symmetry to yield the final 3.1 Å and 3.5 Å maps for 1–23 and 9–71 complex structures, respectively. Cryo-EM data processing parameters and particles counts are reported in Table S4.

#### Cryo-EM structure modeling and refinement

A homology model of the BG505 MD39 SOSIP was generated using SWISS-MODEL^111^ with BG505 SOSIP.v5.2.N241.N289 from PDB 6VKN as a reference structure.^112^ Molecular models for variable fragment (Fv) of RM19R, 9–71 and 1–23 were generated using a structure-based antibody prediction (SAbPred) server^113^ using ABodyBuilder2 algorithm.^114^ The initial Fab-trimer model was generated by rigid body docking the BG505 MD39, RM19R and 1–23/9–71 Fvs into the EM density map using UCSF Chimera X.^97^ The models were then manually rebuilt using Coot^98,115^ and fit into the cryo-EM density using Rosetta Relax.^116^ The final model refinement into the cryo-EM maps was done with Phenix real-space refinement^99^ and evaluated using EMRinger^117^ and MolProbity.^118,119^ The Kabat numbering system was applied for the Fvs using the antibody numbering (Abnum) webserver (http://www.bioinf.org.uk/abs/abnum/),^120^ and the conventional HxB2 numbering for the HIV-1 Env glycoprotein was changed manually using Phenix PDB file editor.^99^ The same workflow was used to determine both complex structures of BG505 MD39-RM19R with 1–23 and 9–71. Final model building statistics are summarized in Table S4.

#### Surface plasma resonance (SPR)

Affinity and kinetics of antibody-antigen interactions were measured on a Carterra LSA using a CMDP Sensor Chip (Carterra) and 1x HBS-EP^+^ pH 7.4 running buffer (20x stock from Teknova, Cat. No H8022) supplemented with BSA at 1 mg/mL. The chip surface for ligand capture was prepared following Carterra software instructions. About 800–1000 RU of capture antibody (SouthernBiotech Cat.no 2047–01) in 10 mM sodium acetate pH 4.5 was amine coupled, and regeneration buffer phosphoric acid 1.7% was injected three times per cycle for 60 s. Ligands (IgGs) were used at 1 μg/mL with a contact time of 5 min. Analyte samples (HIV-1 Envs) were buffer exchanged into the running buffer using dialysis and analyte concentrations were quantified on NanoDrop 2000c Spectrophotometer (Thermo Fisher Scientific) using the absorption signal at 280 nm. Raw sensograms were processed using Kinetics software (Carterra), interspot and blank double referencing, Langmuir model. Best referencing practices depend on how fast the dissociation rate (k_off_) is for a particular ligand-analyte interaction. For slow dissociation rates (9e^−3^ 1/s or less), we utilized manual process referencing that includes serial y-align and lower analyte concentrations. For fast dissociation rates (faster than 9e^−3^ 1/s) we use automated batch referencing that includes overlay y-align and higher analyte concentrations. After automated data analysis by Kinetics software, we also performed additional filtering to remove datasets where highest response signals were smaller than signals from negative controls. This additional filtering was performed automatically using an R-script (available upon request).

#### HIV-1 pseudovirus neutralization assay

Neutralizing activity of monoclonal antibodies was determined as previously described.^46^ Briefly, N-linked glycan knockout mutant and wild-type (WT) pseudoviruses were produced by co-transfection of HEK-293T cells (Thermo Fisher) with Env-bearing and Env-deficient genomic backbone plasmid (pSG3ΔEnv) and harvested 72h post transfection for use in TZM-bl neutralization assays. Plasmids for N-linked glycan knockout mutant pseudoviruses were described previously.^41^ The two pseudotyped Env panels in the current studies were selected to represent the global diversity of historical HIV-1 isolate subtypes and recombinant forms (*N* = 111 virus panel)^121^ and contemporary circulating clade B, C, F1, and BF1 strains derived from the Antibody Mediated Prevention (AMP) trials (NCT02716675 and NCT02568215).^8–10^ The AMP derived strains were specifically selected from the placebo arms of HVTN703 (clade C) and HVTN704 (clades B, F1, BF1) and VRC01-recipients arm of HVTN703 (clade C) for their relative resistance to VRC01 (IC_50_ ≥ 1 μg/mL). A pseudotyped murine leukemia virus (MLV) and a DENV-specific antibody (Den3) were used as pseudovirus and antibody negative controls, respectively. Monoclonal antibody neutralization titers are reported as half-maximum (50%) and 80% inhibitory concentration (IC_50_ and IC_80_) and calculated by comparison with control wells in the absence of antibodies. Analysis and graphing of titration curves were done using Prism 10 software (GraphPad).

#### Antibody-antigen interface calculation

Buried surface area (BSA) calculations of the epitope and paratope protein interfaces were performed using Protein Interfaces, Surfaces, and Assemblies (PISA) service of the European Bioinformatics Institute (EBI).^122^

The antibody footprint of CD4bs-targeting bnAbs was defined as the relative BSA contributions to specific subregions within the CD4bs epitope. Key CD4bs subregions were classified as loop D (274–284), loop V5 (β23-sheet: 451–457, hypervariable V5: 458–465, and β24-sheet: 466–470), CD4-binding loop (362–375), bridging sheets (V2 loop: 194–199, and β20/21-sheets: 423–435), outer domain (OD exit loop: 471–483), inner domain (ID-α1: 96–106), adjacent gp120 (V3 loop: 301–324), adjacent protomer (C1-α0: 60–66, and C2-loop: 205–208). Residues are referred in HxB2 numbering. The average BSA contribution of distinct CD4bs bnAb classes was calculated for all representative VRC01-class and 561-class bnAbs.

The contribution of individual complementarity-determining region (CDR) and framework region (FR) of heavy and light chains to HIV-1 Env binding was determined by addition of the BSA contributions of all residues within the CDR and FR regions according to IMGT definition.

#### Angle of approach measurements

A previously described approach angle measurement methodology was adapted for analysis of a different epitope on HIV-1 Env (CD4-binding site).^80^ The trimer 3-fold axis was defined by a vector intersecting the center of mass (COM) of the CA atoms of R166 and V580 in all three protomers and aligned on the z axis. The COM for the CD4bs (COM-CD4bs) was aligned on the x axis to coordinates (38.139,0,0) as defined by the center of mass of all CA atoms of key CD4bs regions I272-V286,L452-S460,T467-E482,N363-S375 (HxB2 numbering). The COM-CD4bs epitope at the trimer 3-fold axis was aligned to the origin coordinates (0,0,0) and a third COM at coordinates (0,0,28.740) corresponding to the COM for the CA of R166 in all three protomers. The vector intersecting the COM-CD4bs and the COM-CD4bs at the trimer 3-fold axis runs parallel to the x axis (hence the membrane) and perpendicular to the z axis (trimer 3-fold axis). To measure the latitudinal and longitudinal angles of approach a new coordinate system was created with its origin at the COM-CD4bs (38.1,0,0) (Figure S5A). The center of mass of the antibody (COM-Fv) was defined as the COM-HC (CA atoms for the six-stranded β-sheet, residues 18–24, 34–39, 46–52, 67–72, 77–82, 89–94) and the COM-LC (CA atoms for the six-stranded β-sheet, residues 19–24, 33–38, 44–49, 62–67, 70–75, 84–89). The center of mass for the CD4 receptor (COM-CD4) was defined as the COM of the CA atoms of the six-stranded β-sheet residues (67–72,11–17,55–58,36–39,26–30,81–85). CD4bs-targeting bnAbs and non-nAbs with available Ag-Fab complex structures in the PDB database were selected for analysis (Figure S5G,J,N). All gp120-Fab complex structures were aligned on the gp120 to the reference BG505 trimer. The latitudinal angle is defined as the angle formed by the z axis and the vector connecting the COM-CD4bs and the COM-Fv in the y-z plane (Figure S5F). The longitudinal angle is the angle formed by the x axis and the vector connecting the COM-CD4bs and the COM-Fv in the y-x plane (Figure S5I).

#### Binding mode measurements

A new coordinate system was created with its origin at the COM_HC_ for each individual antibody to analyze the HC-LC orientation in regard to the CD4bs epitope (Figure S5B). The HC-LC twist is defined as the angle formed by the y axis and a vector connecting the COM_HC_ and the COM_LC_ in the x-y plane (Figure S5M). It symbolizes the HC-LC orientation around the CD4bs epitope (Figure S5L). Negative angles of 0 to −180 represent an LC rotation to the interior of the trimer against the adjacent protomer, while positive angles of 0 to +180 represent an LC rotation to the exterior of the trimer (Figure S5N). The HC-LC latitudinal angle is the angle formed by the y axis and a vector connecting the COM_HC_ and the COM_LC_ in the y-z plane (Figure S5M). This angle represents HC-LC packing parallel to the trimer 3-fold axis and the LC retraction away from the gp120 subunit (Figure S5L). Positive angles of 0 to +90 correspond to an external rotation of the LC away from the CD4bs epitope. The distance between the light chain and the CD4bs epitope was calculated by the distance between a center of mass at the CA atom of the N276 residue and the COM_LC_ for the representative CD4bs-targeting antibody.

#### Measurement of N276 glycan displacement

An additional coordinate system was generated with its origin at the center of mass of the NAG-C1 (COM_NAG-C1_) atom to analyze the N276 glycan latitudinal and longitudinal orientation in regard to the CD4bs epitope in bound and unbound states (Figure S6A). The N276 glycan latitudinal angle is the angle formed by the y axis and a vector connecting the COM_NAG-C1_ and the center of mass of the MAN-C3 (COM_MAN-C3_) on the y-z plane (Figure S6C). The N276 glycan longitudinal angle is the angle formed by the x axis and a vector connecting the COM_NAG-C1_ and COM_MAN-C3_ on the y-x plane (Figure S6B). The latitudinal displacement of the N276 glycan was calculated by subtracting the N276 glycan latitudinal angles in antibody or CD4 receptor bound states by the angle measured in the unbound state (BG505). Positive angles (0 to +90) correspond to upward motion and negative values (0 to −90) represent a downward displacement away from the light chain interface. Data for CH235.12 is absent because the reference coordinates (N276_CA_ and NAG_C1_) differ substantially from other bnAb-gp120 complex and unbound structures, and any differences in glycan displacement could not be meaningfully compared.

#### Amino acid diversity of HIV-1 Env at specific residues

The amino acid diversity of HIV-1 viruses at positions D279, K282, S365, and D457 (HxB2 numbering) was determined using the Los Alamos National Laboratory (LANL) software Compile, Analyze, and Tally Neutralizing Antibody Panels (CATNAP) based on a total of 8735 Env sequences.^123^

#### Quantification of CD4bs identity among HIV isolates

Amino acid identity to BG505 was calculated separately for key CD4-binding site epitope subregions, including loop D (274–283), CD4-binding loop (362–375), OD exit loop (471–477), V5 loop (455–470), β20/21-bridging sheets (425–432). To account for length variability within the hypervariable V5 loop, insertions or deletions relative to BG505 were treated as amino acid mismatches in the identity calculations. An overall epitope identity score was obtained by averaging identity across all epitope residues, weighted by subregion length.

#### Global analysis of IGHV1–46 allelic variation

The population frequencies for allelic variants of IGHV1–2 and IGHV1–46 was extracted from the Karolinska Institute Adaptive Immune Receptor Variant Atlas (KIARVA) dataset (https://kiarva.scilifelab.se). This resource was compiled from the genotypic analysis of IGHV content of 25 populations from the 1KGP set, following full length functional IGHV sequencing of each case using the ImmuneDiscover protocol, as described in Corcoran et al.,^69^ and the subsequent calculation of appropriate allelic frequencies at the individual population level and the continental-origin based superpopulation level.

#### B cell sorting and paired BCR sequencing

##### Human sample collection

Full leukopaks (three blood volumes) were obtained from healthy, HIV-seronegative human donors. Samples were collected at Hemacare’s Southern California donor center and performed under a protocol approved by the Institutional Research Boards of Scripps Research and Hemacare. Informed consent was obtained from each subject. Immediately upon receipt of the leukopak, peripheral blood mononuclear cells (PBMCs) were isolated by diluting blood about 1:1 with room temperature phosphate-buffered saline (PBS) and layered over Histopaque-1077 (Sigma-Aldrich). Tubes were centrifuged to produce a buffy coat that was then aspirated and moved to a clean tube. PBMCs were washed three times with PBS and then frozen in 9:1 fetal bovine serum (FBS)/dimethyl sulfoxide.

##### Sample preparation and B cell sorting

Frozen PBMCs isolated from a single healthy human donor leukopak (D326651) were rapidly thawed in R50 medium (RPMI-1640 - Gibco, cat# 61870–036, 50% heat inactivated FBS - Omega Scientific, cat# FB-02) pre-warmed to 37°C and washed in FACS buffer (2% v/v heat inactivated FBS, 2 mM EDTA - Invitrogen, cat# 15575–038, 25mM HEPES - Gibco, cat# 15630–080, in Dulbecco’s PBS - Corning, cat # 21–031-CV). After counting, 10 million cells were stained with 100 μL antibody cocktail mix consisting of APC-Cy7 anti-CD3 (BioLegend, cat# 317342), APC-Cy7 anti-CD4 (BioLegend, cat# 317418), APC-Cy7 anti-CD8 (BD Biosciences, cat# 557760), APC-H7 anti-CD14 (BD Biosciences, cat# 561384), APC-H7 anti-CD16 (BD Biosciences, cat# 560195), APC-Cy7 anti-CD56 (BioLegend cat# 362512), PE anti-CD19 (BD Biosciences, cat# 555413), PerCP-Cy5.5 anti-IgD (BD Biosciences, cat# 561315), BV786 anti-IgG (BD Biosciences, cat# 563230), and PE-Cy7 anti-IgM (BioLegend cat# 314532), All antibodies were used at 1:40 dilution. Following antibody staining, live/dead stain (LIVE/DEAD Fixable Aqua, Invitrogen, cat# L34966) was added and the sample incubated for an additional 15 min at 4°C. At the end of staining, cells were washed with FACS buffer and resuspended in 500 μL FACS buffer. Samples were filtered through a 35 μm mesh-cap FACS tube (Falcon, cat# 352235) prior to being sorted using a BD FACS Melody. Sorting gates were drawn to select for live B cells defined as Aqua- DUMP- (CD3/4/8/14/16/56-) CD19^+^ IgD-cells. A total of 203,400 IgD- B cells were sorted using purity mode into PCR plates containing 20 μL of 0.2 μm filtered FBS per well. Three operators separately sorted 100, 250, 500, 700, 1000, 2500, 7500, or 10000 sorted cells per well. This process was repeated three times, resulting in 72 individual sorts, which were processed into 72 GEMs as described below. This experimental design deviates from a traditional workflow and was originally designed to evaluate the qualification limits and robustness of the 10X Genomics platform in anticipation of introducing this technology to evaluate antigen-specific B cell responses in the IAVI G003 clinical trial (Willis et al. Science 2025). We opportunistically took advantage of the experimental data to provide an additional source of paired B cell data for this manuscript.

##### BCR sequencing using 10x genomics

Sorted samples were prepared for BCR sequencing by the 10x Genomics Single Cell Immune Profiling platform. After cell sorting, DPBS was added up to near top of the sample collection well (about 100 μL) and gently mixed to dilute the FBS catch buffer. The plate was sealed, and cells were centrifuged for 2 min at 2000 rpm, after which the excess buffer was removed except for about 38 μL required for the 10x Genomics GEM reaction. Samples were processed on a 10x Genomics Chromium Controller according to manufacturer’s user guide for Chromium Next GEM Single Cell 5′ Reagent Kits v2 (Dual Index) with Feature Barcoding, with two main modifications. The number of PCR cycles in the cDNA amplification step were determined by assuming that only 20% of the total number of cells sorted would be recovered. On average, the number of unique paired-BCR sequences actually recovered from the 72 unique reactions was typically about 25% of the total number of cells sorted. In the second modification, the number of PCR cycles for each of the V(D)J amplification steps were increased to 10 cycles if the number of cells sorted was fewer than 1000 cells. Details regarding the modified protocol can be found in Hurtado et al.^124^ Pooled libraries were sequenced on an Illumina NextSeq 2000 using a 100-cycle P3 reagent kit (Illumina, cat# 20040559) with a target depth of 5000 paired-end reads for the V(D)J and run using read parameters indicated in the 10x Genomics user guide. Raw sequencing data were demultiplexed and assembled into V(D)J contigs, using Cell Ranger (v6.1).

#### Bioinformatic processing of paired BCR sequences

In this work we probed the dependence on specific HCDR3 motifs, diversity D genes, junction J genes, and lengths for HIV-1 neutralization by a specific class of CD4bs-targeting bnAbs dependent on IGHV1–46 and IGKV3–20 variable genes. To simulate the natural sequence and structural diversity that originates from V(D)J-recombination within IGHV1–46/IGKV3–20-paired BCRs in the human antibody repertoire, we assembled a paired BCR sequence database based on PBMC samples from healthy, unvaccinated subjects^125,126^ available on the Observed Antibody Space (OAS) server^127^ along with paired BCR sequences from DeKosky et al.^128^ and those generated in house as described above. IGHM constant region calls were added to the DeKosky dataset to account for reads from naive B cells lacking sequence coverage in the constant region. The BCR immunoglobulin (Ig) gene assignments were determined using Sequencing Analysis and Data library for Immunoinformatics Exploration (SADIE, https://github.com/jwillis0720/sadie),^55^ and paired BCR heavy and light chains and their characteristics were stored in the standardized Adaptive Immune Receptor Repertoire (AIRR) format.^129^ The entire bioinformatics pipeline was documented in a repository (https://github.com/SchiefLab/Bader2025). The database contains 524,694 paired BCR sequences of which 450,968 are from naive B cells. We filtered the paired BCR sequence dataset for IGHV1–46/IGKV3–20-paired BCRs to obtain HCDR3 sequences most representative of bona-fide V_H_1–46/V_K_3–20-derived antibodies in the human antibody repertoire. Random IGHV1–46/IGKV3–20-paired BCRs were subsequently selected and their HCDR3 sequence was used to generate HCDR3-chimeric 1–23 variant antibodies.

#### Bioinformatic calculation of CD4bs bnAb precursor frequencies

CD4bs bnAb precursor frequencies were calculated using naive paired BCR data from four donors (D326651, Donor-3, Donor-D1, Donor-D2) with adequate sequence coverage (Figure 3K). Two donors (Donor-D3 and Healthy-1) were excluded from our dataset due to insufficient sequence counts (<3000 total sequences). The immunogenetic definitions applied for the frequency estimates were: 561-class type I (IGVH1–46/IGKV3–20, 9-residue LCDR3); 561-class type II (IGVH1–46/IGKV3–20, 10-residue LCDR3); CH235-class (IGVH1–46/IGKV3–15, 8-residue LCDR3); 1B2530-class (IGVH1–46/IGLV1–47, 11-residue LCDR3); VRC01-class (IGVH1–2 paired with IGKV3–20/IGKV3–15/IGKV1–5/IGLV1–33/IGLV2–14/IGLV1–11, 5-residue LCDR3); and IOMA-class (IGVH1–2/IGLV2–23, 8-residue LCDR3).

### QUANTIFICATION AND STATISTICAL ANALYSIS

No statistical methods or predetermined sample sizes were employed in this study.

## Supplementary Material

1

2

3

4

SUPPLEMENTAL INFORMATION

Supplemental information can be found online at https://doi.org/10.1016/j.celrep.2026.117180.

## Figures and Tables

**Figure 1.**
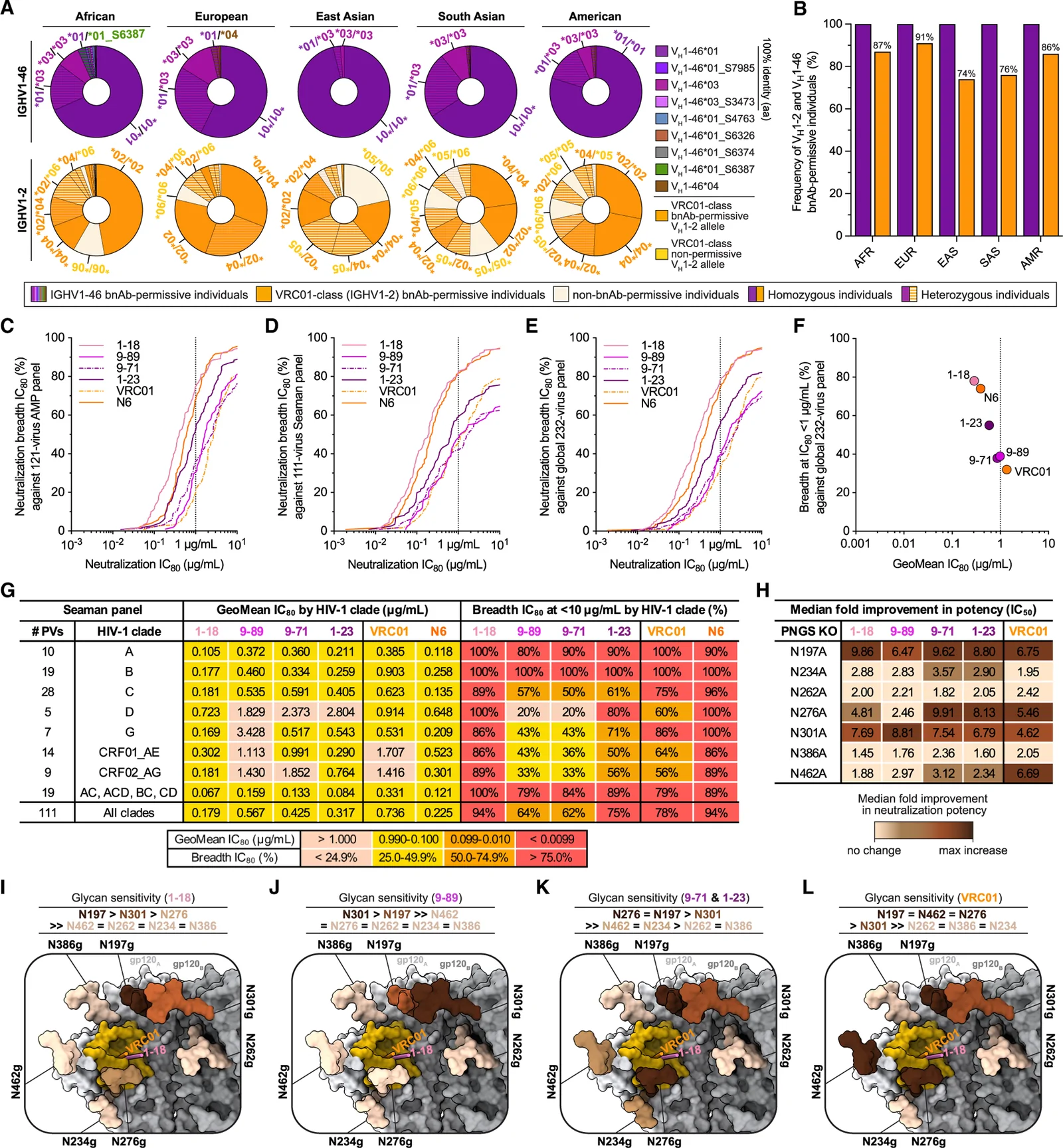
IGHV1–46 conservation enables universal vaccine strategies targeting V_H_1–46/V_K_3–20-derived bnAbs with broad HIV-1 neutralization and distinct glycan sensitivity profiles (A) Frequency of individuals in five 1KGP superpopulations (African [AFR], European [EUR], East Asian [EAS], South Asian [SAS], and American [AMR]) carrying specific IGHV1–2 and IGHV1–46 allele pairs. (B) Frequency of individuals in five 1KGP superpopulations (African, European, East Asian, South Asian, and American) carrying bnAb-permissive (BP) and non-bnAb-permissive (NBP) alleles for V_H_1–2 and V_H_1–46 genes (color coded as indicated). (C) Neutralization breadth (%) plotted as a function of potency (neutralization IC_80_ in μg/mL) for VRC01, N6, 9–89, 1–18, 9–71, and 1–23 bnAbs tested against a 121-virus panel of multi-clade AMP-derived HIV-1 pseudoviruses. (D) Neutralization breadth (%) plotted as a function of potency (neutralization IC_80_ in μg/mL) for VRC01, N6, 9–89, 1–18, 9–71, and 1–23 bnAbs tested against a global 111-virus Seaman panel of HIV-1 pseudoviruses. (E) Neutralization breadth (%) plotted as a function of potency (neutralization IC_80_ in μg/mL) for VRC01, N6, 9–89, 1–18, 9–71, and 1–23 bnAbs tested against the combined panel of 232 viruses. (F) Neutralization breadth (% isolates neutralized at IC_80_ < 1 μg/mL) versus potency (GeoMean IC_80_) for VRC01, N6, 9–89, 1–18, 9–71, and 1–23 bnAbs tested against a 232-virus global panel of AMP and Seaman HIV-1 isolates. (G) Summary of neutralization potency (GeoMean IC_80_) and breadth (%) by HIV-1 clade for VRC01, N6, 9–89, 1–18, 9–71, and 1–23 bnAbs against a cross-clade 111-virus Seaman panel of HIV-1 pseudoviruses. Values are colored according to the legend. (H) Median fold improvement in neutralization potency (IC_50_) by the indicated bnAbs against N-linked glycan KO vs. WT pseudoviruses. Values represent the median improvement across five HIV-1 isolates. Blue gradient intensity indicates the median fold improvement (white = no change, dark blue = maximum median improvement). (I–L) Structural illustration depicting the degree of sensitivity toward N-linked glycans surrounding the CD4-binding site by 1–18 (G), 9–89 (H), 9–71, 1–23 (I), and VRC01 (J) antibodies. Colors reflect fold improvement values from (F) upon glycan removal, indicating glycan-imposed hindrance (beige = weak hindrance, copper brown = medium hindrance; sienna = high hindrance; dark brown = highest hindrance). Approach vectors reveal proximity of 1–18 (PDB: 6UDJ; pink) and VRC01 (PDB: 3NGB; orange) to representative N-linked glycans.

**Figure 2.**
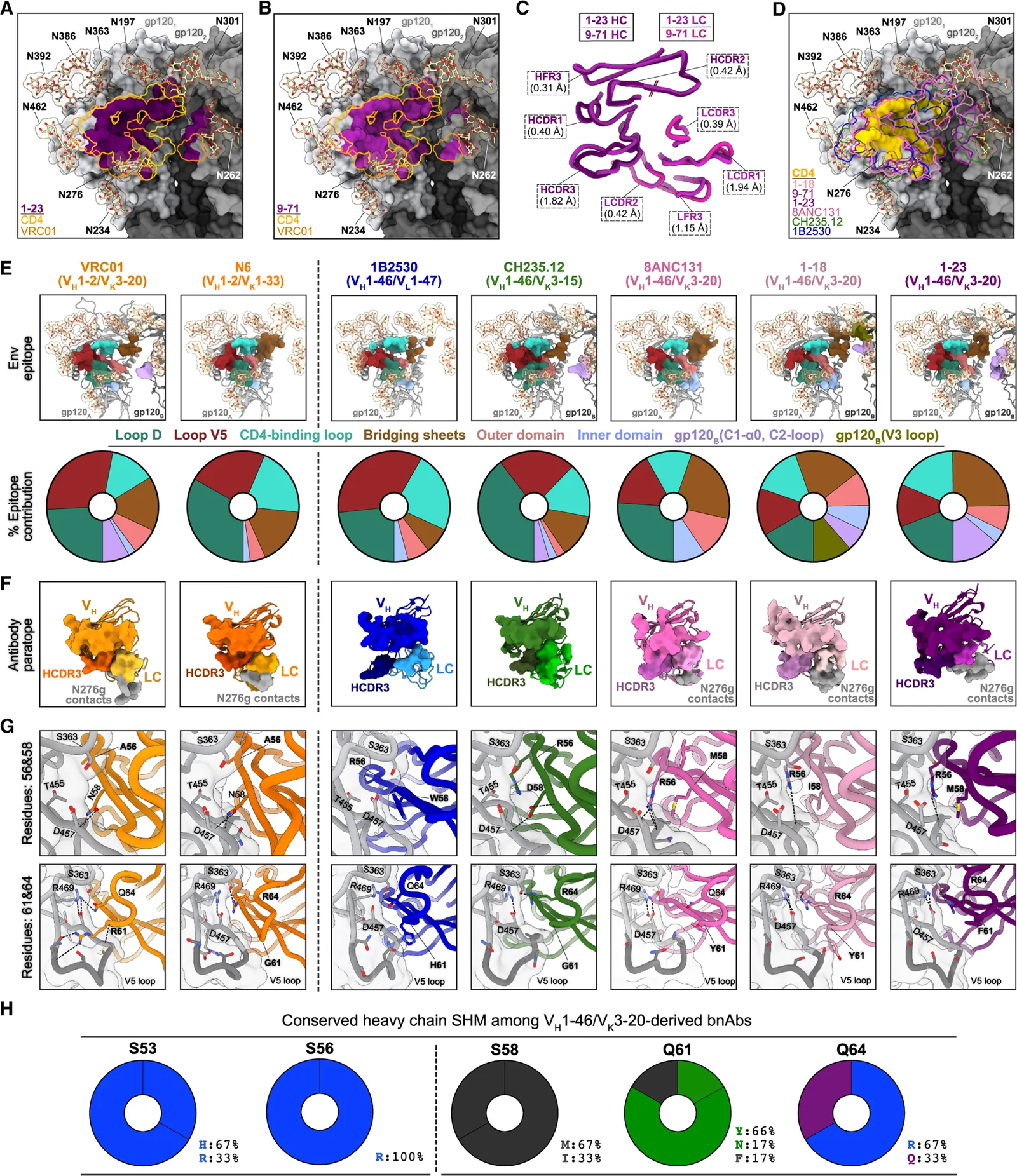
V_H_1–46/V_K_3–20-derived bnAbs exhibit distinctive antibody footprints and conserved heavy-chain affinity maturation (A) The 1–23 epitope (dark purple) shows substantial overlap with VRC01 (PDB: 5FYJ; orange outline) and CD4 receptor (PDB: 5VN3; yellow outline) epitopes. Epitope residues were defined using Protein Interfaces, Surfaces, and Assemblies (PISA) analysis. Glycans from CD4bs-unbound Env (PDB: 5FYL; beige) were overlaid to demonstrate steric occlusion of the supersite. (B) The 9–71 epitope (light purple) reveals substantial overlap with VRC01 (PDB: 5FYJ; orange outline) and CD4 receptor (PDB: 5VN3; yellow outline) epitopes. Epitope residues were defined using PISA analysis. (C) Structural overlay of 1–23 and 9–71 Fabs reveals almost identical binding modes. Cα root-mean-square deviation (RMSD) values for key paratope motifs are labeled in dashed boxes. Red vertical lines separate HCDR2 and HFR3 regions. (D) Magnified view of the CD4 receptor epitope (yellow) shows substantial overlap across known V_H_1–46 bnAbs 1B2530 (PDB: 4YFL), CH235.12 (PDB: 8SXJ), 8ANC131 (PDB: 4RWY), 1–18 (PDB: 6UDJ), 1–23, and 9–71. (E) Epitope footprints on HIV-1 Env trimers for representative V_H_1–2-derived bnAbs VRC01 (PDB: 5FYJ) and N6 (PDB: 5TE6) and V_H_1–46-derived bnAbs 1B2530 (PDB: 4YFL), CH235.12 (PDB: 8SXJ), 8ANC131 (PDB: 4RWY), 1–18 (PDB: 6UDJ), and 1–23, showing epitope surfaces (top) and relative BSA contributions by CD4bs subregions (bottom). (F) Antibody paratope for the above bnAb-Env complexes visualized as a surface rendering of contact residues. Darker color tones distinguish HCDR3 from V_H_-derived paratope residues. Light-chain paratope residues exclusively contacting the N276 glycan are shown in gray. (G) Key interactions of VRC01-class bnAbs VRC01 (PBD: 3NGB) and N6 (PBD: 5TE6) and V_H_1–46-derived bnAbs 1B2530 (PDB: 4YFL), CH235.12 (PDB: 8SXJ), 8ANC131 (PDB: 4RWY), 1–18 (PDB: 6UDJ), and 1–23 with HIV-1 gp120 (light gray) by HCDR2 and HFR3 paratope residues. Key contacts formed by somatically mutated heavy-chain residues are highlighted in bold. Expected hydrogen bonds and salt bridges using a cutoff distance of 3.2 Å are shown as dotted lines. Heavy- and light-chain residues are numbered according to the Kabat numbering scheme. (H) Amino acid frequency at key HCDR2 and HFR3 residues for six V_H_1–46/V_K_3–20-derived bnAbs (1–18, 1–55, 8ANC131, 8ANC134, 1–23, and 9–71) reveals conserved SHM patterns among antibodies. The germline sequence is shown at the top and the SHM residues at the bottom right corner of each representative pie chart.

**Figure 3.**
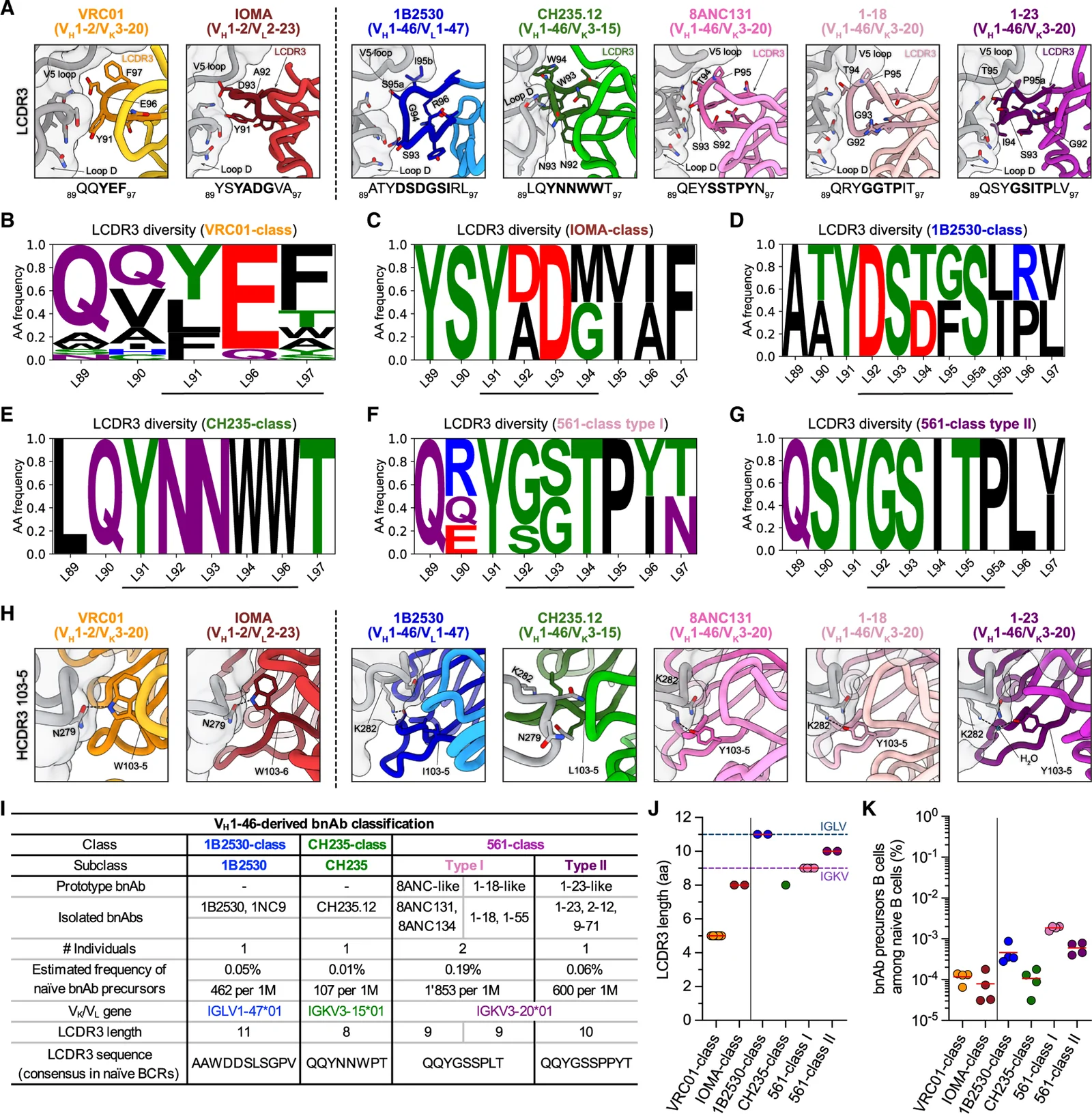
Light-chain pairing dictates V_H_1–46 bnAb signatures and precursor frequencies (A) Cartoon representation of LCDR3-mediated interactions with HIV-1 gp120 by antibodies representing distinct CD4bs bnAb classes (VRC01 [PDB: 3NGB], IOMA [PDB: 5T3Z], 1B2530 [PDB: 4YFL], CH235.12 [PDB: 8SXJ], 8ANC131 [PDB: 4RWY], 1–18 [PDB: 4RWY], and 1–23). A dashed black line separates V_H_1–2- and V_H_1–46-derived bnAbs. Heavy- and light-chain residues are numbered according to the Kabat numbering scheme. (B–G) LCDR3 sequence logos for VRC01-class (B), IOMA-class (C), 1B2530-class (D), CH235-class (E), 561-class type I (F), and 561-class type II (G) bnAbs. Black lines indicate key paratope residues that contact gp120. Heavy- and light-chain residues are numbered according to the Kabat numbering scheme. (H) Cartoon illustration of critical HCDR3-mediated contacts with loop D by antibodies representing distinct CD4bs bnAb classes. VRC01-class bnAbs (orange) employ a conserved Trp, while 561-class bnAbs (pink and purple) use a Tyr at position 103–5. (I) Immunogenetic definition of diverse classes and subclasses of V_H_1–46-derived bnAbs. (J) Dot plot showing the LCDR3 length employed by CD4bs-targeting bnAbs. A red horizontal line represents the average loop length. Purple and blue dashed lines represent the most common LCDR3 length in the human IGKV and IGLV repertoire, respectively. (K) Frequency of putative naive B cell precursors for V_H_1–2-derived (VRC01 and IOMA-class) and V_H_1–46-derived (561-, CH235-, and 1B2530-class) bnAbs among total IgM+ naive B cells in four donors (each dot represents one individual).

**Figure 4.**
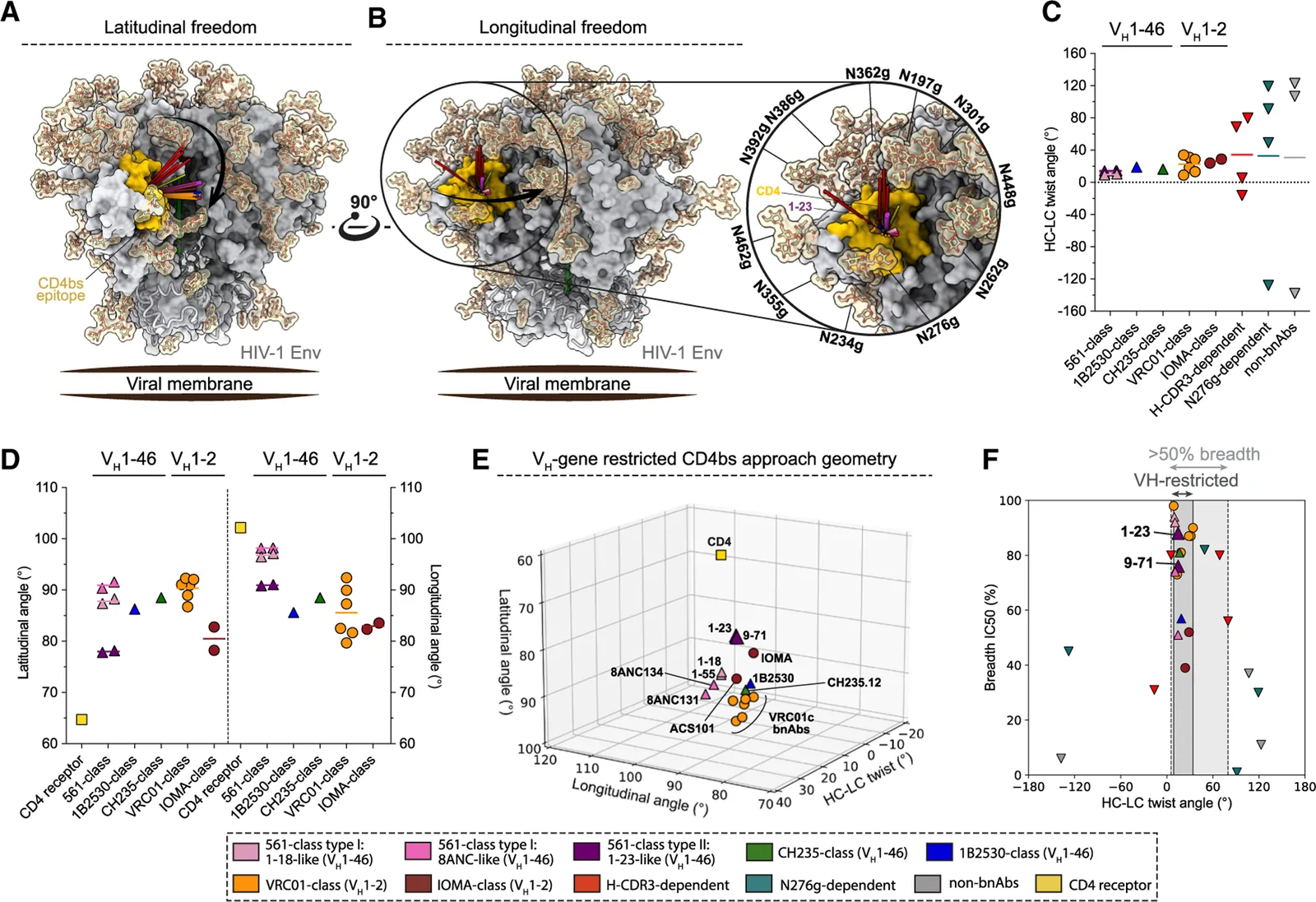
561-class type I and II bnAbs adopt distinct angles of approach to trimer binding (A and B) Vectors connecting the center of mass (COM) of the variable fragment (Fv) domain and the CD4-binding site COM contrast the conserved angle of approach adopted by V_H_-dependent bnAbs with the variable CD4bs recognition by HCDR3-dependent antibodies. CD4 receptor engagement is depicted as a yellow vector for reference. All Fab-gp120 structures were overlaid on the CD4bs-undbound HIV-1 Env structure (PDB: 5FYL), and its glycans (beige) illustrate steric occlusion of the supersite. (C) V_H_1–2 and V_H_1–46-derived bnAbs recognize the CD4bs with a conserved HC-LC twist, with the light chain facing the plane of the membrane. (D) The latitudinal and longitudinal angle of approach by V_H_1–2 and V_H_1–46-derived bnAb classes. (E) A 3D scatterplot depicts the spatial distribution of the COM-Fv of each CD4bs-targeting antibody in complex with the CD4bs epitope. (F) The conserved HC-LC orientation correlated with improved HIV-1 neutralization breadth. The light gray area represents the HC-LC twist that achieves >50% breadth of currently known CD4bs bnAbs. The dark gray area depicts the HC-LC twist angles adopted by V_H_-dependent bnAbs.

**Figure 5.**
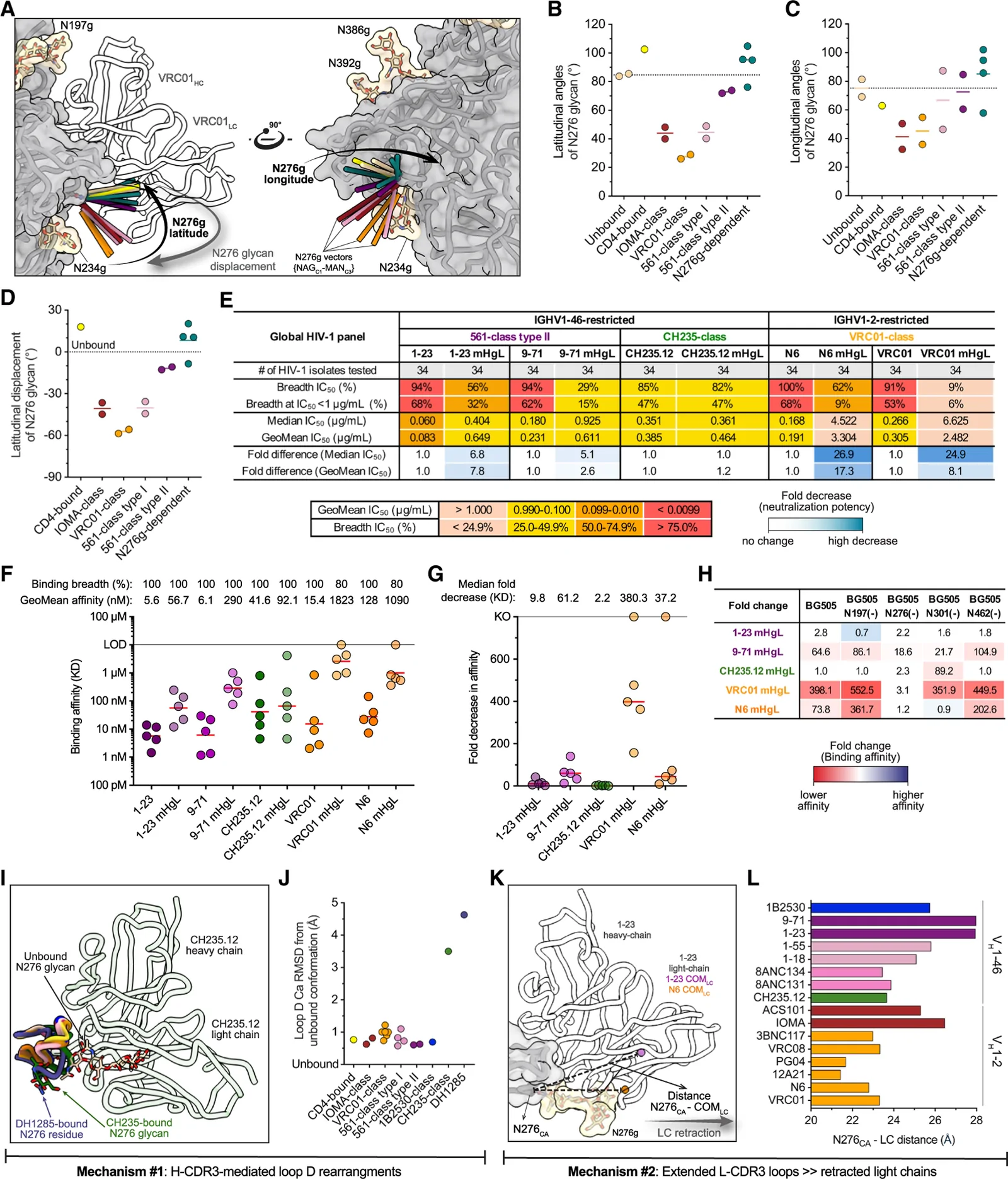
Two distinct mechanisms enable V_H_1–46 bnAbs to overcome the N276 glycan barrier (A) Latitudinal (left) and longitudinal (right) angles of the N276 glycan in CD4bs bnAb-bound and unbound states. A vector from COM-NAG_C1_ to COM-MAN_C3_ defines the N276 glycan orientation when bound to CD4 receptor (PDB: 5VN3; yellow), IOMA-class bnAbs (IOMA [PDB: 5T3Z] and ACS101 [PDB: 7Z3A], brown), VRC01-class bnAbs (VRC01 [PDB: 5FYJ] and 3BNC117 [PDB: 5V8M], orange), 561-class type I bnAbs (1–18 [PDB: 6UDJ] and 1–55 [PDB: 6UDK], pink), and 561-class type II bnAbs (1–23 and 9–71, purple), N276 glycan-dependent (N276g-dependent) bnAbs (VRC40 [PDB: 7LL1], CAP257-RH1 [PDB: 5T33], 179NC75 [PDB: 7LLK], and VRC33 [PDB: 7LL2], teal) compared to the unbound native conformation (BG505 [PDB: 5FYL] and JR-FL [PDB: 5FUU], beige). (B) Dot plot showing the latitudinal angles of the N276 glycan in bnAb-bound and unbound states. Lines represent the average latitudinal angle calculated for each antibody class. (C) Dot plot depicting the longitudinal angles of the N276 glycan in bnAb-bound and unbound states. Lines represent the average longitudinal angle calculated for each antibody class. (D) Dot plot revealing the latitudinal displacement of the N276 glycan imposed by antibody engagement compared to the average unbound latitudinal angle (dotted line). (E) Table summarizing the neutralization efficacy by mature and GL-reverted light chain variants (mHgL) of five CD4bs-targeting bnAbs against a cross-clade 34-virus HIV-1 panel. Values are colored according to the legend, and blue gradient intensity represents fold reduction neutralization potency (white = no change, dark blue = maximum reduction). (F) Dot plot visualizing the binding affinity of mature and GL-reverted light chain variants (mHgL) of 561-class (purple), CH235-class (green), and VRC01-class (orange) antibodies against a panel of five WT stabilized trimers. The geometric mean affinity and binding breadth are highlighted at the top of the graph. (G) Dot plot showing the median fold decrease in binding affinity of 561-class (purple), CH235-class (green), and VRC01-class (orange) antibodies upon reverting the light-chain mutations to the germline residues. (H) Table highlighting the fold change in binding affinity of mature vs. mHgL antibodies against WT BG505 and four N-linked glycan KO trimer variants. The fold change is depicted by a red-to-blue gradient, with blue and red representing improvement and reduction in affinity, respectively. (I) Cartoon representation of the loop D rearrangement induced by CH235.12 (PDB: 5F96; green) and DH1285 engagement (PDB: 8U1D; dark lavender) displacing the N276 glycan away from the light chain. (J) Dot plot representing the RMSD of loop D Cα atoms (residues 274–284, HxB2 numbering) upon binding to CD4bs bnAbs and the CD4 receptor compared to the CD4bs-unbound conformation. (K) Structure schematic illustrating distance between the N276_CA_ atom and the light-chain center of mass (COM_LC_) for VRC01 (PDB: 3NGB) and 1–23 bnAbs. (L) Histogram showing the N276_CA_-COM_LC_ distances in IOMA-class (brown), VRC01-class (orange), 561-class type I (pink), 561-class type II (purple), CH235-class (green), and 1B2530-class (blue) antibody CD4bs recognition.

**Figure 6.**
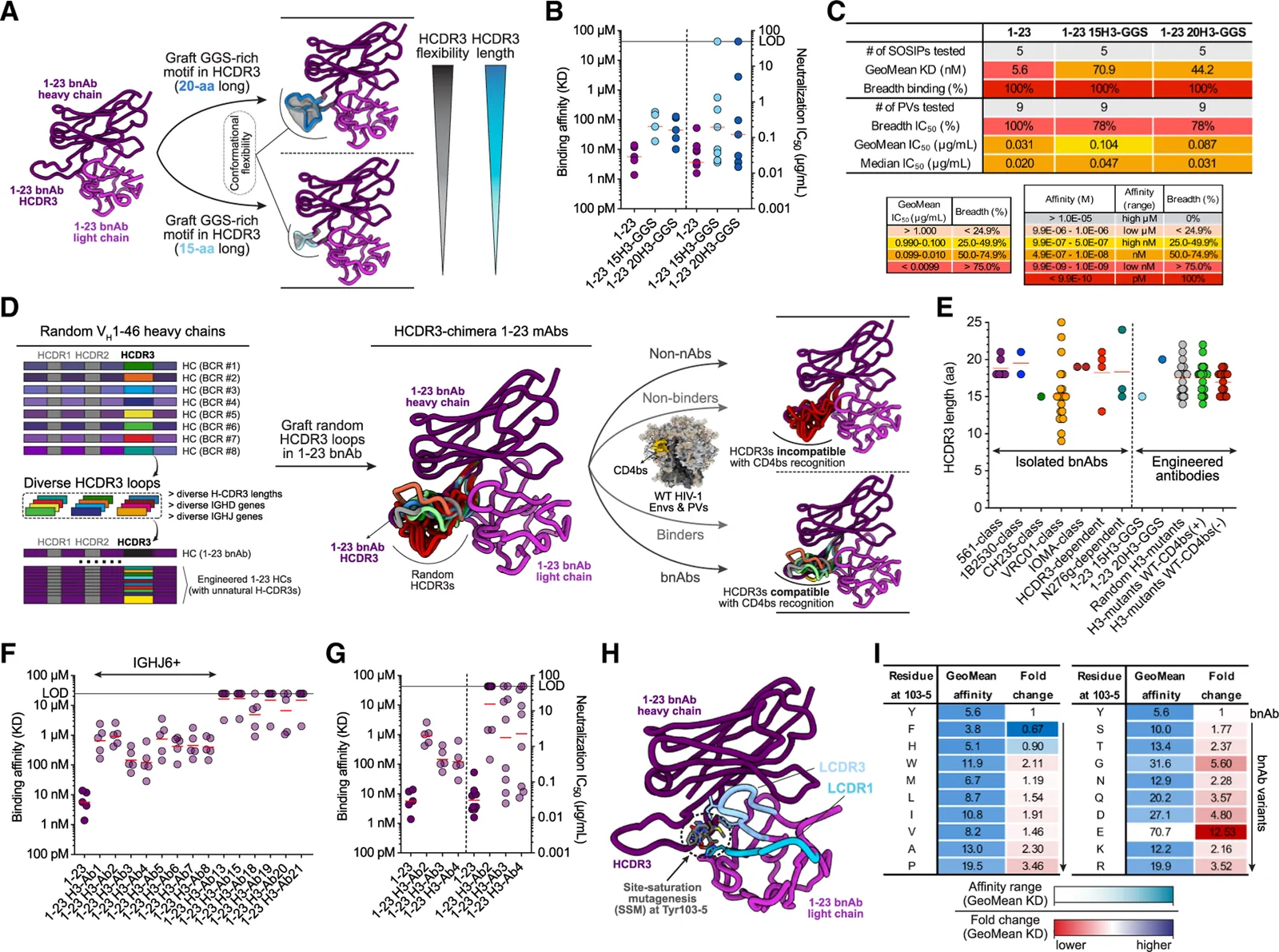
CD4bs recognition by 561-class bnAbs is V_H_ gene dependent (A) Schematic illustrating engineered 1–23 variants with 15- and 20-residue-long GGS-rich HCDR3 loops. (B) Dot plot displaying the binding affinity and neutralization potency by 1–23 bnAb (purple) and engineered GGS-rich monoclonal antibodies (light and dark blue) against a panel of five WT stabilized trimers (001428, 3000–090-pblib002, DU422–1, BG505, and CNE40) and nine WT pseudo-typed HIV-1 viruses (TRO.11, DU422–1, 16055–2.3, CE1176-A3, CNE21, Q23.17, BJOX009000.02.4, X1194_C1, and 6480.v4.c25). Red horizontal lines indicate geometric mean affinity and potency. (C) Summary of geometric mean affinity and HIV-1 neutralizing capacity for experimental data plotted in (B). Values are colored according to the legend. (D) Schematic of the strategy to demonstrate that 561-class HIV-1 neutralization is primarily V_H_ gene dependent. Diverse HCDR3 loops from randomly selected IGHV1–46/IGKV3–20-paired BCR sequences from the Observed Antibody Space (OAS) database were grafted onto 1–23 to generate 22 unique bnAb variants. Variants were categorized by their ability to maintain CD4bs recognition through binding and neutralization assays. (E) HCDR3 length distribution of CD4bs bnAbs: 561-class (purple), 1B2530-class (blue), CH235-class (green), VRC01-class (orange), IOMA-class (brown), HCDR3-dependent (red), N276g-dependent bnAbs (teal), and engineered 1–23 HCDR3 variants. (F) Dot plot showing the binding affinity of 1–23 (purple) and HCDR3 variants (light purple) against a panel of five WT stabilized trimers. The geometric mean affinity is shown as a red line. (G) Dot plot displaying the binding affinity and neutralization potency by 1–23 bnAb (purple) and three representative HCDR3 variant mAbs (light purple) against a panel of five WT stabilized trimers and nine WT pseudo-typed HIV-1 viruses. Geometric mean values for affinity and neutralization potency are indicated by red horizontal lines. (H) Cartoon representation of 1–23 Fv, depicting site-saturation mutagenesis (SSM) at key HCDR3 residue at position 103–5, where variants containing each amino acid (except cysteine) were expressed and tested for Env binding. (I) Geometric mean affinity (K_D_) and fold change upon single point mutation at residue Y103–5 when tested against a panel of five stabilized Env trimers. The fold change is depicted by a red-to-blue gradient, with blue and red representing improvement and reduction in affinity, respectively. Blue gradient intensity represents highest to lowest geometric mean affinity (white = lowest, dark blue = highest).

**Figure 7.**
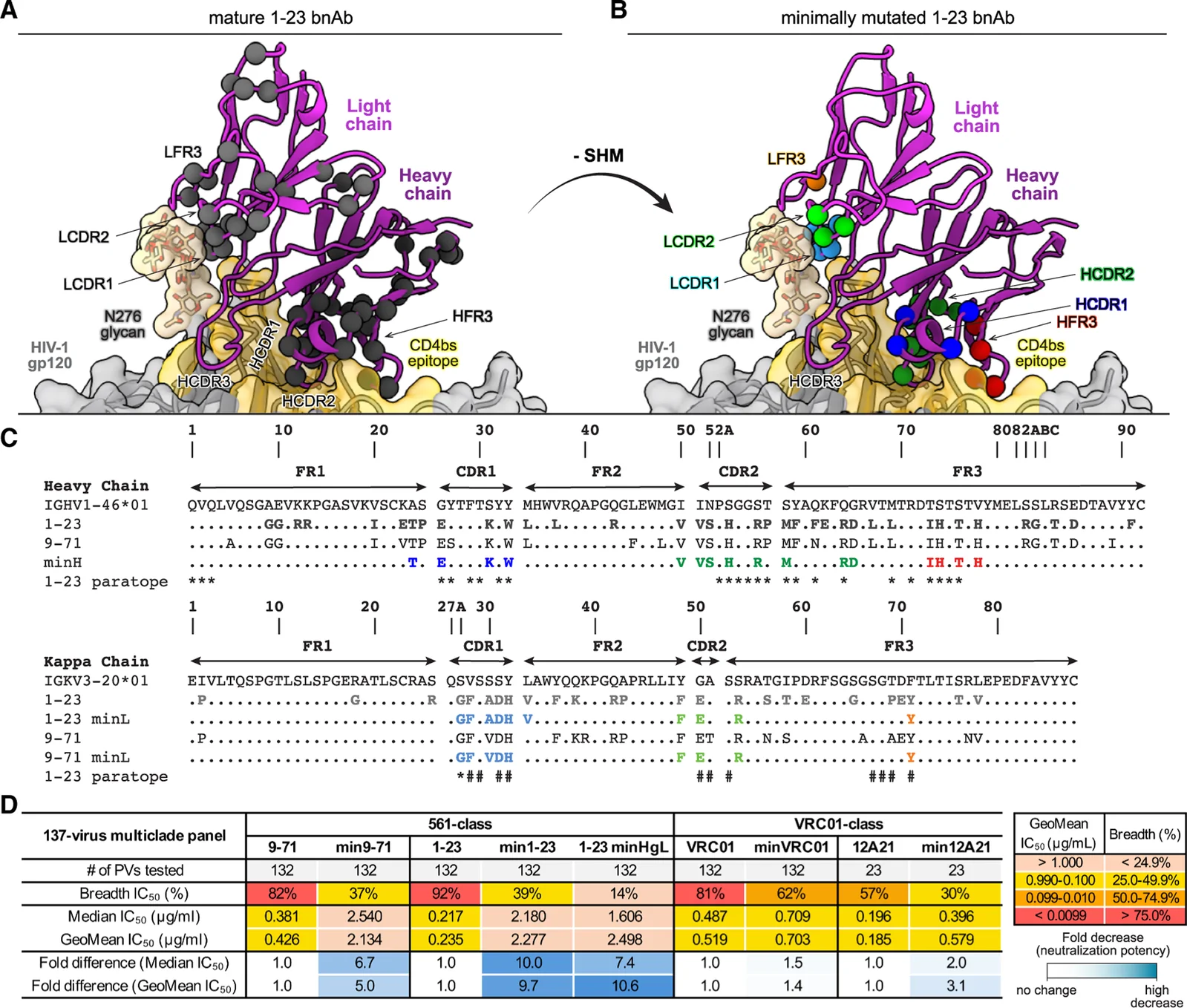
Minimally mutated 561-class bnAbs guide reductionist vaccine design (A) The cryo-EM structure of 1–23 (purple) in complex with the BG505 gp120 protomer (gray), displaying its interactions with the CD4bs (yellow) and the N276 glycan (beige) and the extensive SHM in the light (dark gray) and heavy (black) chains. (B) The same structure as in (A), displaying the key mutations in the light and heavy chains that are required for HIV-1 neutralization. Mutations are color coded in patches distinguished by unique colors. (C) Alignment of 1–23, 9–71, min1–23, and min9–71 with their germline variable genes (IGHV1–46*01 and IGKV3–20*01), highlighting key mutations for min9–71 and min1–23 with the identical colors as used in (B). The 1–23 antibody paratope is highlighted below the V_H_ and V_K_ sequence alignments, showing gp120 contacts (*) and N276 glycan (#) contacts. (D) Table summarizing the neutralization breadth and potency of 561- and VRC01-class bnAbs and corresponding minimally mutated variants against a 137-virus multi-clade HIV-1 panel. Blue gradient intensity represents fold reduction in geometric mean and median IC_50_ values (white = no change, dark blue = maximum reduction).

**Table:** KEY RESOURCES TABLE

REAGENT or RESOURCE	SOURCE	IDENTIFIER
Antibodies		

1–23	Schommers et al.^26^	N/A
min1–23	This manuscript.	N/A
1–23 mHgL	This manuscript.	N/A
1–23 minHgL	This manuscript.	N/A
1–23 mH1mL	This manuscript.	N/A
1–23 mH2mL	This manuscript.	N/A
1–23 mFR3mL	This manuscript.	N/A
1–23 mH1-H2mL	This manuscript.	N/A
1–23 mH1-FR3mL	This manuscript.	N/A
1–23 mH2-FR3mL	This manuscript.	N/A
1–23 mH1gL	This manuscript.	N/A
1–23 mH2gL	This manuscript.	N/A
1–23 mFR3gL	This manuscript.	N/A
1–23 mH1-H2gL	This manuscript.	N/A
1–23 mH1-FR3gL	This manuscript.	N/A
1–23 mH2-FR3gL	This manuscript.	N/A
1–23 20H3-GGS	This manuscript.	N/A
1–23 15H3-GGS	This manuscript.	N/A
1–23 H3-Ab1	This manuscript.	N/A
1–23 H3-Ab2	This manuscript.	N/A
1–23 H3-Ab3	This manuscript.	N/A
1–23 H3-Ab3	This manuscript.	N/A
1–23 H3-Ab5	This manuscript.	N/A
1–23 H3-Ab6	This manuscript.	N/A
1–23 H3-Ab7	This manuscript.	N/A
1–23 H3-Ab8	This manuscript.	N/A
1–23 H3-Ab9	This manuscript.	N/A
1–23 H3-Ab10	This manuscript.	N/A
1–23 H3-Ab11	This manuscript.	N/A
1–23 H3-Ab12	This manuscript.	N/A
1–23 H3-Ab13	This manuscript.	N/A
1–23 H3-Ab14	This manuscript.	N/A
1–23 H3-Ab15	This manuscript.	N/A
1–23 H3-Ab16	This manuscript.	N/A
1–23 H3-Ab17	This manuscript.	N/A
1–23 H3-Ab18	This manuscript.	N/A
1–23 H3-Ab19	This manuscript.	N/A
1–23 H3-Ab20	This manuscript.	N/A
1–23 H3-Ab21	This manuscript.	N/A
1–23 H3-Ab22	This manuscript.	N/A
9–71	Schommers et al.^26^	N/A
min9–71	This manuscript.	N/A
9–71 mHgL	This manuscript.	N/A
9–89	Schommers et al.^26^	N/A
1–18	Schommers et al.^26^	N/A
CH235.12	Bonsignori et al.^25^	N/A
CH235.12 mHgL	This manuscript.	N/A
PC63–71I1a	Umotoy et al.^41^	N/A
N6	Huang et al.^33^	N/A
N6 mHgL	This manuscript.	N/A
12A21	Scheid et al.^21^	N/A
Min12A21	Jardine et al.^36,51^	N/A
VRC01	Scheid et al.^21^	N/A
VRC01 mHgL	This manuscript.	N/A
minVRC01	Jardine et al.^36,51^	N/A
PCT64–35S	Landais et al., 2017^86^	N/A
PCT64–18D	Landais et al., 2017^86^	N/A
CH01	Bonsignori et al., 2012^87^	N/A
PG16	Pejchal et al., 2010^88^	N/A
PG9	Pejchal et al., 2010^88^	N/A
PGT145	Walker et al., 2011^89^	N/A
10E8	Huang et al., 2012^90^	N/A
min10E8	Schiffner et al.^56^	N/A
LN01	Pinto et al., 2019^91^	N/A
DH511	Williams et al., 2017^92^	N/A
PGT151	Falkowska et al., 2014^93^	N/A
VRC23	Wu et al.^19^	N/A
PGT121	Walker et al., 2011^89^	RRID: AB_2491041
PGT130	Doores et al., 2014^94^	N/A
BG18	Freund et al., 2017^95^	N/A
minBG18.11	Steichen et al.^54^	N/A
Anti Human CD3 APC-Cy7	BD Bioscience	Cat# 557757; RRID_AB:396863
Anti Human CD4 APC-Cy7	BD Bioscience	Cat# 566913; RRID_AB:2739681
Anti Human CD8 APC-Cy7	BD Biosciences	Cat# 557760; RRID_AB:400383
Anti Human CD14 APC-H7	BD Biosciences	Cat# 561384; RRID_AB:10611720
Anti Human CD16 APC-H7	BD Biosciences	Cat# 560195; RRID_AB:1645466
Anti Human CD56 APC-Cy7	Biolegend	Cat# 362512; RRID:395813
Anti Human CD19 PE	BD Biosciences	Cat# 555413; RRID_AB:395813
Anti Human IgD PerCP-Cy5.5	BD Biosciences	Cat# 561315; RRID_AB:10646033
IgG BV786	BD Biosciences	Cat# 564230; RRID_AB:2874964
IgM PE-Cy7	Biolegend	Cat# 314532; RRID_AB:737291
Human Hashtag 1 (C0251) - TotalSeq C	Biolegend	Cat# 394661; RRID_AB:2801031
Human Hashtag 2 (C0252) - TotalSeq C	Biolegend	Cat# 394663; RRID_AB:2801032
Human Hashtag 3 (C0253) - TotalSeq C	Biolegend	Cat# 394665; RRID_AB:2801033
Human Hashtag 4 (C0254) - TotalSeq C	Biolegend	Cat# 394667; RRID_AB:2801034
Human Hashtag 5 (C0255) - TotalSeq C	Biolegend	Cat# 394661; RRID_AB:2801035
Human Hashtag 6 (C0256) - TotalSeq C	Biolegend	Cat# 394663; RRID_AB:2820042
Human Hashtag 7 (C0257) - TotalSeq C	Biolegend	Cat# 394665; RRID_AB:2820043
Human Hashtag 8 (C0258) - TotalSeq C	Biolegend	Cat# 394667; RRID_AB:2820044
Human Hashtag 9 (C0259) - TotalSeq C	Biolegend	Cat# 394669; RRID:AB_2820045
Human Hashtag 10 (C0260) - TotalSeq C	Biolegend	Cat# 394671; RRID:AB_2820046

Bacterial and virus strains		

DH5α Competent Cells	Thermo Fisher Scientific	Cat # GACC-96

Biological samples		

none	–	–

Chemicals, reagents, peptides, and recombinant proteins		

BG505 MD39 SOSIP	Steichen et al.^96^	N/A
BG505 MD39 SOSIP N197Q	This manuscript.	N/A
BG505 MD39 SOSIP N276Q	This manuscript.	N/A
BG505 MD39 SOSIP N301Q	This manuscript.	N/A
BG505 MD39 SOSIP N462Q	This manuscript.	N/A
3000 RnS.4 SOSIP	This manuscript.	N/A
DU422–1 MD39.2 SOSIP	This manuscript.	N/A
CNE40 MD39.2 SOSIP	This manuscript.	N/A
001428 MD39.2 SOSIP	Cottrell et al.^46^	N/A
20X HBS-EP+ Buffer, pH 7.6 (1 L, sterile)	Teknova	Cat# H8022
Water (Optima LC-MS grade)	Fisher Scientific	Cat# W6–4
IgG Elution Buffer	Thermo Fisher Scientific	Cat# 210099
Opti-MEM	Thermo Fisher Scientific	Cat# 31985–088
Bambanker Freezing Media BB01	Fisher Scientific (Bulldog Bio Inc.)	Cat# 50–999-554
PEI MAX Transfection Grade Linear Polyethylenimine Hydrochloride (MW 40,000)	Polysciences (Kyfora Bio)	Cat# 24765
Human Capture Antibody	Southern Biotech	Cat# 2047–01
Endosafe Compendial LAL Cartridges, 0.05, 10/pk	Fisher Scientific	Cat# NC9594798
Phosphoric Acid 85 wt.% in H_2_O	Thermo Fisher Scientific	Cat# 345245–500ML
Sodium Acetate pH 4.5, 10 mM	Carterra	Cat# 3622
Tween 20	Millipore Sigma	Cat# P1379
Bovine Serum Albumin (BSA), Protease free	Gold Biotechnologies	Cat# A-420–10
PBS pH 7.4 (1X)	Life Technologies	Cat# 10010049
Tris-buffered saline (10X)	Thermo Scientific	Cat# J60764
rProtein A Sepharose Fast Flow resin	Cytiva	Cat# 17127903
FreeStyle 293 Expression Medium	Thermo Fisher Scientific	Cat# 12338026
Pierce^™^ Protease Inhibitor Tablets, EDTA-free	Thermo Fisher Scientific	Cat# A32965
5M Sodium chloride (500 mL)	Corning	Cat# 46–032-CV
1M Tris-HCl, pH 8.0 solution. (1 L, Sterile)	Teknova	Cat# T1080
Bovine Serum Albumin (BSA)	Fisher (Rockland)	Cat# NC0263771
Ampicillin Sodium Salt (Crystalline Powder)	Fisher Scientific	Cat# BP1760–25
Carterra LSA Sensor Chip (CMDP)	Carterra	Cat# 4282
Amine Coupling Kit	Cytiva	Cat# BR100050
Biacore^™^ Maintenance Kit, type 3	Cytiva	Cat# 29229054
Dialyzer Midi	VWR	Cat# 71506–3
Lauryl Maltose Neopentyl Glycol (LMNG)	Anatrace	Cat# NG310
Penicillin-Streptomycin (10′000 U/mL)	Thermo Fisher Scientific	Cat# 15140122
L-Glutamine (200 mM)	Thermo Fisher Scientific	Cat# 25030081
Dulbecco’s Modified Eagle Medium (DMEM)	Thermo Fisher Scientific	Cat# 10313039
Heat Inactivated FBS	Omega Scientific	Cat# FB-02
FuGENE 6 Transfection Reagent	FuGENE Inc.	Cat# F6–5000
DEAE-Dextran	Thermo Fisher Scientific	Cat# 9015–73-0
Trypsin-EDTA (0.25%), phenol red	Thermo Fisher Scientific	Cat# 25200056
1X Dulbecco’s Phosphate-Buffered Saline (DPBS)	Corning	Cat# 21–031-CV
Streptavidin AF647	Invitrogen	Cat# S11223
Streptavidin-AF488	Invitrogen	Cat# S21374
Streptavidin-BV421	Biolegend	Cat# 405225
BD FC Beads BV510	BD Biosciences	Cat# 661628
BD FC Beads APC-H7	BD Biosciences	Cat# 661621
BD FC Beads APC-Cy7	BD Biosciences	Cat# 661622
BD FC Beads PE	BD Biosciences	Cat# 661616
BD FC Beads PerCP Cy 5.5	BD Biosciences	Cat# 661619
BD FC Beads BV786	BD Biosciences	Cat# 661630
BD FC Beads PE Cy7	BD Biosciences	Cat# 661617
BD FC Beads FITC	BD Biosciences	Cat# 661615
BD FC Beads APC	BD Biosciences	Cat# 661620
BD FC Beads BV421	BD Biosciences	Cat# 661627
BD Resuspension Buffer (35mL)	BD Biosciences	Cat# 661614
Spherotech Anti-Human IgG/Protein Beads	Spherotech	Cat# HUP1505
Anti-Mouse Ig, κ/Negative Comp Beads	BD Biosciences	Cat# 552843
Mouse anti-human IgG	BD Biosciences	Cat# 555784
UltraComp Beads Plus Compensation Beads	Thermo Fisher Scientific	Cat# 01–333-42
ArC Reactive Beads	Invitrogen	Cat# A10346
Biotin Polystyrene Particles	Spherotech	Cat# TP-30–5
Glycerol, molecular grade	Ricca Chemical Company	Cat# 3290–32
Buffer EB	Qiagen	Cat# 19086
Ethanol, 100 Proof	Pharmco	Cat# 111000200
SPRI Beads	Beckman Coulter	Cat# B23319
Dynabeads	Invitrogen	Cat# 37002D
Ambion Nuclease-free Water	Invitrogen	Cat# AM9937
RPMI	Gibco	Cat# 61870–036
EDTA	Invitrogen	Cat# 15575–038
CS&T Beads	BD Biosciences	Cat# 661414
Accudrop beads	BD Biosciences	Cat# 661612
Sheath Fluid	BD Biosciences	Cat# 99–30211-00
High Sensitivity D5000 ScreenTape	Agilent	Cat# 5067–5592
High Sensitivity D5000 Reagents	Agilent	Cat# 5067–5593
High Sensitivity D1000 ScreenTape	Agilent	Cat# 5067–5584
High Sensitivity D1000 Reagents	Agilent	Cat# 5067–5585
PhiX Control Library NextSeq V3	Illumina	Cat# FC-110–3002
NextSeq 2000 P3 Reagents (100 Cycles)	Illumina	Cat# 20040559
Bovine Serum Albumin 30% Solution	Fisher (Rockland)	Cat# NC0263771
Microdialysis Plate 48 Wells 1 mL	Thermo Fisher Scientific	Car# A50466

Deposited data		

BG505 MD39 + RM19R Fab + 1–23 Fab model	PDB: 9PIT	This manuscript.
BG505 MD39 + RM19R Fab + 1–23 Fab map	EMD: 71677	This manuscript.
BG505 MD39 + RM19R Fab + 9–71 Fab model	PDB: 9PIV	This manuscript.
BG505 MD39 + RM19R Fab + 9–71 Fab map	EMD: 71678	This manuscript.
Paired BCR Dataset	https://github.com/SchiefLab/Bader2025	N/A
Commercial assays		
Zymo PURE II Plasmid Maxiprep kit	Zymo Research	Cat#D4203-B
Luciferase Assay Systems	Promega	Cat# E1501
EasySep^™^ Human Pan-B Cell Enrichment Kit	Stem Cell	Cat# 19554
Live/Dead Fixable Aqua	Invitrogen	Cat# L34966
Chromium Next GEM Chip K Single Cell Kit	10X Genomics	Cat# 1000286
Chromium Next GEM Single Cell 5ʹ Kit v2	10X Genomics	Cat# 1000263
5′ Feature Barcode Kit, 16 rxns	10X Genomics	Cat# 1000286
Single Cell Human BCR Amplification Kit	10X Genomics	Cat# 1000286
Dual Index Kits	10X Genomics	Cat# 1000215/1000250
Qubit 1x dsDNA HS Assay Kit	Invitrogen	Cat# Q33231
Cell lines		
Human: Freestyle 293F cells	Invitrogen	Cat#R79007
Human: TZM-bl cells	NIH AIDS Reagent Program	Cat#8129
Human: 293T cells	ATCC	Cat#CRL-3216

Experimental models: Organisms/strains		

none	–	–

Oligonucleotides		

none	–	–

Recombinant DNA		

pHL-sec	–	Addgene #99845
pCW-sec	–	N/A
pCW-CHIg-hG1	–	N/A
pCW-CLIg-hk	–	N/A

Software and algorithms		

UCSF ChimeraX v1.10.1	Goddard et al., 2021	https://www.cgl.ucsf.edu/chimerax/; RRID: SCR_015872
UCSF Chimera v1.15	Pettersen et al.^97^	https://www.cgl.ucsf.edu/chimera/; RRID: SCR_004097
PyMol	Schrodinger	RRID: SCR_000305
Carterra Software	Carterra Inc.	N/A
Coot	Casañal et al.^98^	http://www2.mrc-lmb.cam.ac.uk/personal/pemsley/coot/; RRID: SCR_014222
Phenix software suite (v1.21.2)	Liebschner et al.^99^	https://www.phenix-online.org; RRID: SCR_014224
Leginon	Suloway et al.^100^	https://sbgrid.org/software/titles/leginon; RRID: SCR_016731
CryoSPARC	Punjani et al.^101^	https://cryosparc.com; RRID: SCR_016501
FlowJo v10.9.0_CL	BD Bioscience	https://www.flowjo.com
R package	R Foundation for Statistical Computing	https://www.r-project.org
Office 365	https://www.office.com/	N/A
Illustrator	Adobe	N/A
GraphPad Prism v10.2.1	GraphPad	RRID: SCR_002798

Other		

NanoDrop 2000c Spectrophotometer	Thermo Fisher Scientific	ND-2000
BD FACSMelody Cell Sorter	BD Bioscience	N/A
Octet RED96e Instrument	FortéBio	RED96E
Dawn HELEOS II	Wyatt	N/A
Optilab T-REX	Wyatt	N/A
HisTrap HP column (5 mL)	Cytiva	Cat# 17524801
Superdex 200 Increase 10/300 GL	Cytiva	Cat# 28990944
HiLoad 16/600 Superdex 200 prep grade column	Cytiva	Cat# 28989335
Endosafe nexgen-PTS Instrument	Charles River	N/A
Microdialysis plate 48-wells 1 mL	Thermo Fisher Scientific	Cat# A50466
Amicon^®^ Ultra Centrifugal Filter, 30 kDa MWCO (15 mL)	Millipore Sigma	Cat# UFC9030
Amicon^®^ Ultra Centrifugal Filter, 30 kDa MWCO (4 mL)	Millipore Sigma	Cat# UFC8030
Amicon^®^ Ultra Centrifugal Filter, 100 kDa MWCO (15 mL)	Millipore Sigma	Cat# UFC9100
Amicon^®^ Ultra Centrifugal Filter, 100 kDa MWCO (4 mL)	Millipore Sigma	Cat# UFC8100
UltrAuFoil 1.2/1.3–300 mesh holey gold foil EM grids	Electron Microscopy Sciences	Cat# Q350AR13A
PELCO easiGlow^™^ Glow Discharge Cleaning System	Ted Pella, Inc.	N/A
Vitrobot Mark IV System	Thermo Fisher Scientific	N/A
BioTek Synergy HTX Multimode Reader	Agilent	N/A
BD FACS Melody with ACDU collection plate	BD Biosciences	N/A
10X Controller + Accessories	10X Genomics	Cat# 1000202
C1000 Touch Thermal Cycler	BioRad	Cat# 1851197
TapeStation 4200	Agilent	Cat# G29991AA
Qubit^™^ 4 Fluorometer	Invitrogen	Cat# Q33238
NextSeq Sequencer	Illumina	N/A

## Highlights

- IGHV1–46 alleles offer universal bnAb-permissive coverage across global populations
- V_H_1–46 bnAb classification guides rational vaccine template selection
- V_H_1–46 holds germline features distinct from V_H_1–2, requiring gene-specific immunogen design
- V_H_1–46 bnAb precursor priming necessitates class-specific priming immunogens
