# Supplementary material for: Structural and immunogenetic signatures guide CD4-mimetic HIV vaccine development
Source: Cell Rep. Author manuscript; Available in PMC 2026 Oct 30. (PMC13720660; doi:10.1016/j.celrep.2026.117180)
Supplement: Supplementary file 1 [file NIHMS2170784-supplement-1.pdf]

Cell Reports, Volume *45*

Supplemental information

Structural and immunogenetic signatures guide CD4-mimetic HIV vaccine development

Daniel L.V. Bader, Claudia T. Flynn, Oleksandr Kalyuzhniy, Gabriel Ozorowski, Martin M. Corcoran, Alison Burns, Pilar Altman, Romy Rouzeau, Troy Sincomb, Alessia Liguori, Monica L. Fernández-Quintero, Johannes R. Loeffler, Jonathan L. Torres, Hannah L. Turner, Erik Georgeson, Amelia Zhou, Hannah Voic, Sue Goo, Lara Shahin, Iszac Burton, Mengyu Wu, Robyn L. Stanfield, Saman Eskandarzadeh, Danny Lu, Nushin Alavi, Nicole Phelps, Ryan Tingle, Katherine McKenney, John Youhanna, Sonya Amirzehni, Torben Schiffner, Jon M. Steichen, Dennis R. Burton, Ian A. Wilson, Gunilla B. Karlsson Hedestam, Elise Landais, Jeong Hyun Lee, Devin Sok, Christopher A. Cottrell, Andrew B. Ward, and William R. Schief

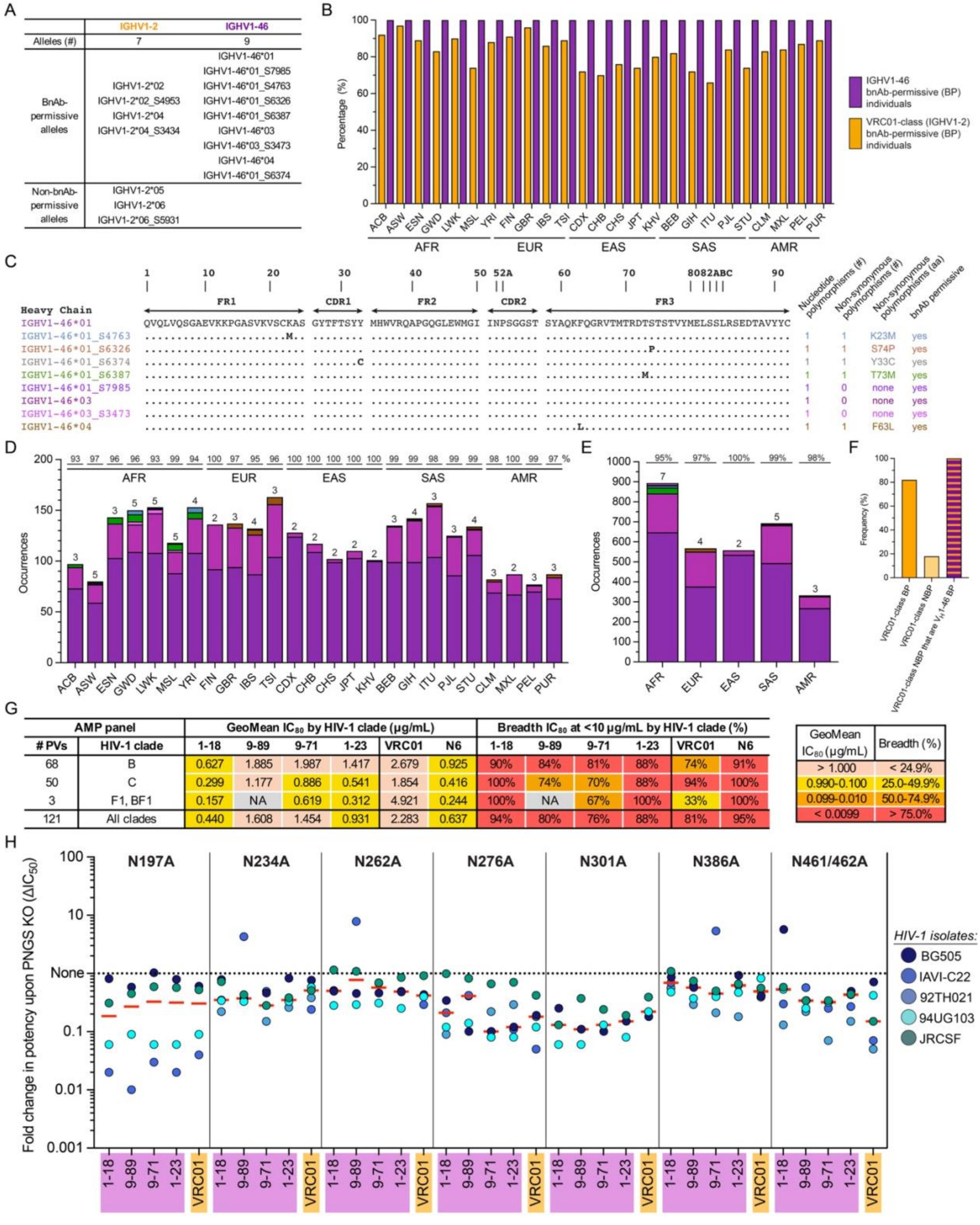

Figure S1. IGHV1-46 conservation enables universal vaccine strategies targeting V_H1-46/V_K3-20-derived bnAbs with broad HIV-1 neutralization and distinct glycan sensitivity profiles. Related to Figure 1.

(A) Table listing all bnAb-permissive and non-bnAb-permissive alleles for IGHV1-2 and IGHV1-46 genes identified in the 1KGP.

(B) Frequency of individuals across 25 1KGP subpopulations carrying bnAb-permissive (BP) and non-bnAb-permissive (NBP) alleles for V_H1-2 and V_H1-46 genes (color-coded as indicated).

(C) Amino acid sequence alignment of the nine IGHV1-46 alleles identified in 1KGP, using IGHV1-46*01 as reference, with summary table listing nucleotide and amino acid polymorphism counts relative to IGHV1-46*01 and classification of bnAb-permissiveness.

(D) Distribution of IGHV1-46 alleles across the twenty five 1KGP populations (D) and five superpopulations (E) (color-coded as indicated). Numbers above bars represent the total number of IGHV1-46 alleles identified in that population or superpopulation. The percentage of IGHV1-46*01 homologous alleles among all identified IGHV1-46 alleles in each population or superpopulation is displayed at the top of the panel, above each bar.

(F) Frequency of individuals that are VRC01-class IGHV1-2 bnAb-permissive (BP), VRC01-class IGHV1-2 non-bnAb-permissive (NBP), and the frequency of VRC01-class non-bnAb-permissive individuals that are IGHV1-46 bnAb permissive.

(G) Summary of neutralization potency (GeoMean IC_{80}) and breadth (%) by HIV-1 clade for VRC01, N6, 9-89, 1-18, 9-71, and 1-23 bnAbs against a 121-virus AMP-derived panel of HIV-1 pseudoviruses. Values are colored according to the legend.

(H) Fold change in neutralization potency (GeoMean IC_{50}) for VRC01-class (orange) and 561-class (purple) antibodies against eight different CD4bs PNGS-KO pseudoviruses versus wild-type HIV-1 isolates. Red lines depict median fold change across isolates, and colors indicate specific HIV-1 isolates: BG505 (navy blue), IAVI-C22 (royal blue), 92TH021 (sky blue), 94UG103 (cyan), JRCSF (turquoise).

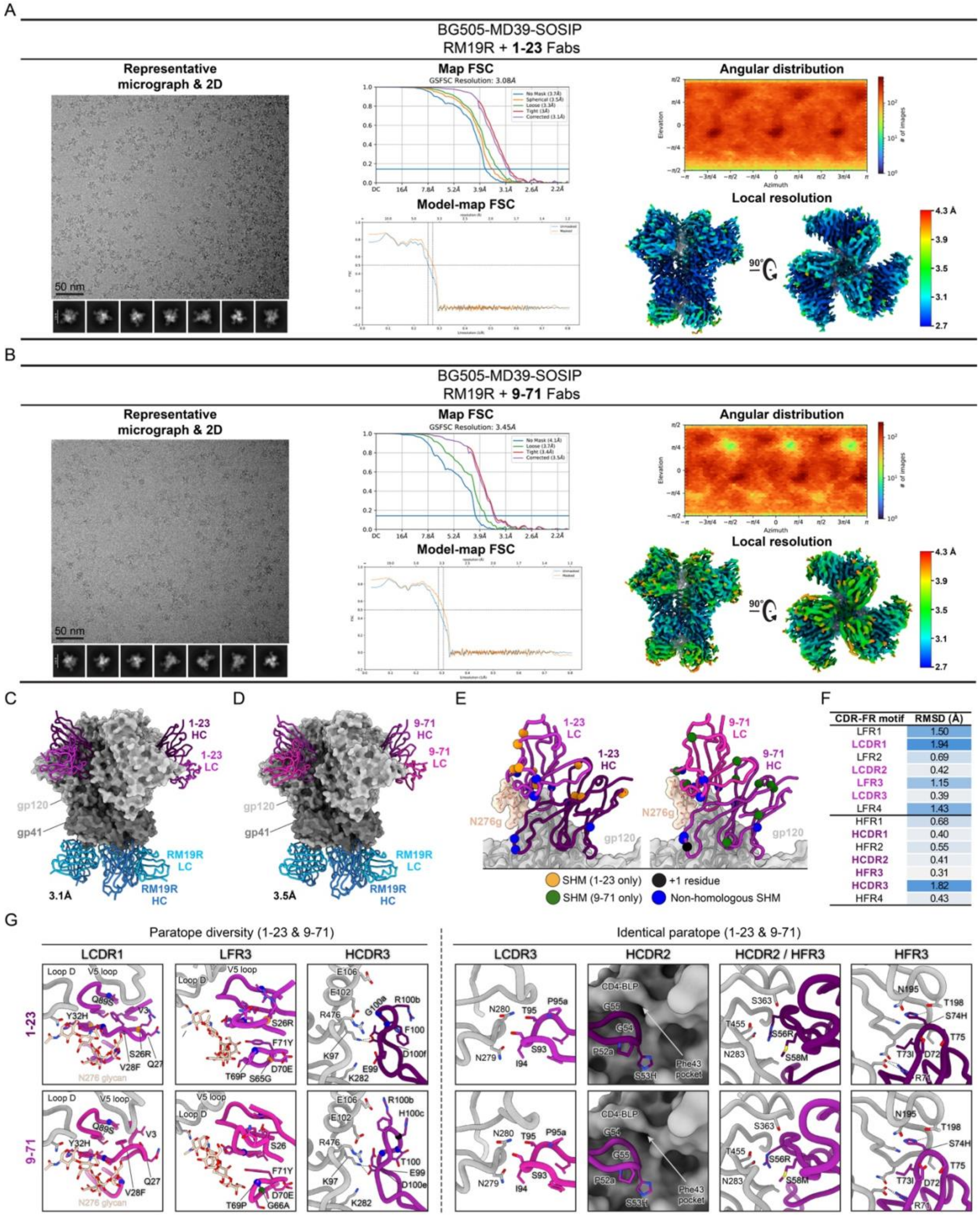

Figure S2. Cryo-EM reconstruction statistics and structural details of for 1-23 and 9-71 Fabs in complex with RM19R Fab and BG505 MD39. Related to Figure 2.

(A) Cryo-EM data for 1-23 Fab in complex with RM19R Fab and BG505 MD39 showing: representative dose weighted micrograph (Gatan K2 Summit), 2D class averages, gold-standard FSC curves of the final 3D reconstruction, atomic model-to-map FSC curves, angular distribution plots, and final 3D reconstruction colored by local resolution. Color gradient indicates local resolution estimate: red (>4.0 Å), green (3.2-3.8 Å), blue (2.7-3.1 Å).

(B) Cryo-EM data for 9-71 Fab in complex with RM19R Fab and BG505 MD39 showing: representative dose weighted micrograph (Gatan K2 Summit), 2D class averages, gold-standard FSC curves of the final 3D reconstruction, atomic model-to-map FSC curves, angular distribution plots, and final 3D reconstruction colored by local resolution. Color gradient indicates local resolution estimate: red (>4.0 Å), green (3.2-3.8 Å), blue (2.7-3.1 Å).

(C) Atomic model of 1-23 (purple ribbons) and RM19R (blue ribbons) Fabs bound to BG505 MD39 (grey surface rendering).

(D) Atomic model of 9-71 (purple ribbons) and RM19R (blue ribbons) Fabs bound to BG505 MD39 (grey surface rendering).

(E) Structural comparison of 1-23 (left) and 9-71 (right) bnAbs demonstrating identical CD4bs binding modes. gp120 (grey ribbons/surface) and N276 (beige atoms/surface) are shown. Color-coded spheres indicate SHM differences: 1-23-specific (orange), 9-71-specific (green), non-homologous (blue), and loop extensions (black).

(F) Cα root mean square deviation (RMSD) values for all heavy and light chain framework (FR) and complementarity-determining regions (CDRs) of 1-23 and 9-71 Fabs, illustrating overlapping binding modes with moderate differences in loop conformation at HCDR3, LCDR1, and LFR3.

(G) Comparison of 1-23 and 9-71 Fab interactions with BG505 gp120 (light grey) showing moderately diverse (LCDR1, LFR3, HCDR3; left) versus near-identical (LCDR3, HCDR2, HFR3; right) paratope regions. Highlighted is the location of the CD4 Phe43 cavity on the surface of the gp120 CD4bs.

A

IGHV gene	Antibody	Heavy chain interface (BSA) gp120$_A$	gp120$_B$	Total	Light chain interface (BSA) gp120$_A$	N276	Total	
V_H1-2	VRC01	1011	90	1101	199	374	573	
	N6	863	NA	863	201	114	315	*
	ACS101	926	7	933	330	140	469	
	IOMA	835	0	835	217	140	357	
V_H1-46	1B2530	783	NA	783	306	NA	306	*
	CH235.12	967	72	1039	364	0	364	
	8ANC131	1016	NA	1016	209	93	302	*
	8ANC134	1050	NA	1050	178	99	277	*
	1-18	1108	261	1368	170	420	589	
	1-55	1133	250	1383	156	181	337	
	1-23	886	165	1051	161	388	548	
	9-71	1010	106	1115	182	340	522	
V_H4-59	CH103	822	150	972	277	0	277	
V_H3-30	HJ16	611	NA	611	367	60	427	*
V_H1-69	VRC13	919	NA	919	24	0	24	*
V_H2-23	VRC16	901	NA	901	302	0	302	*

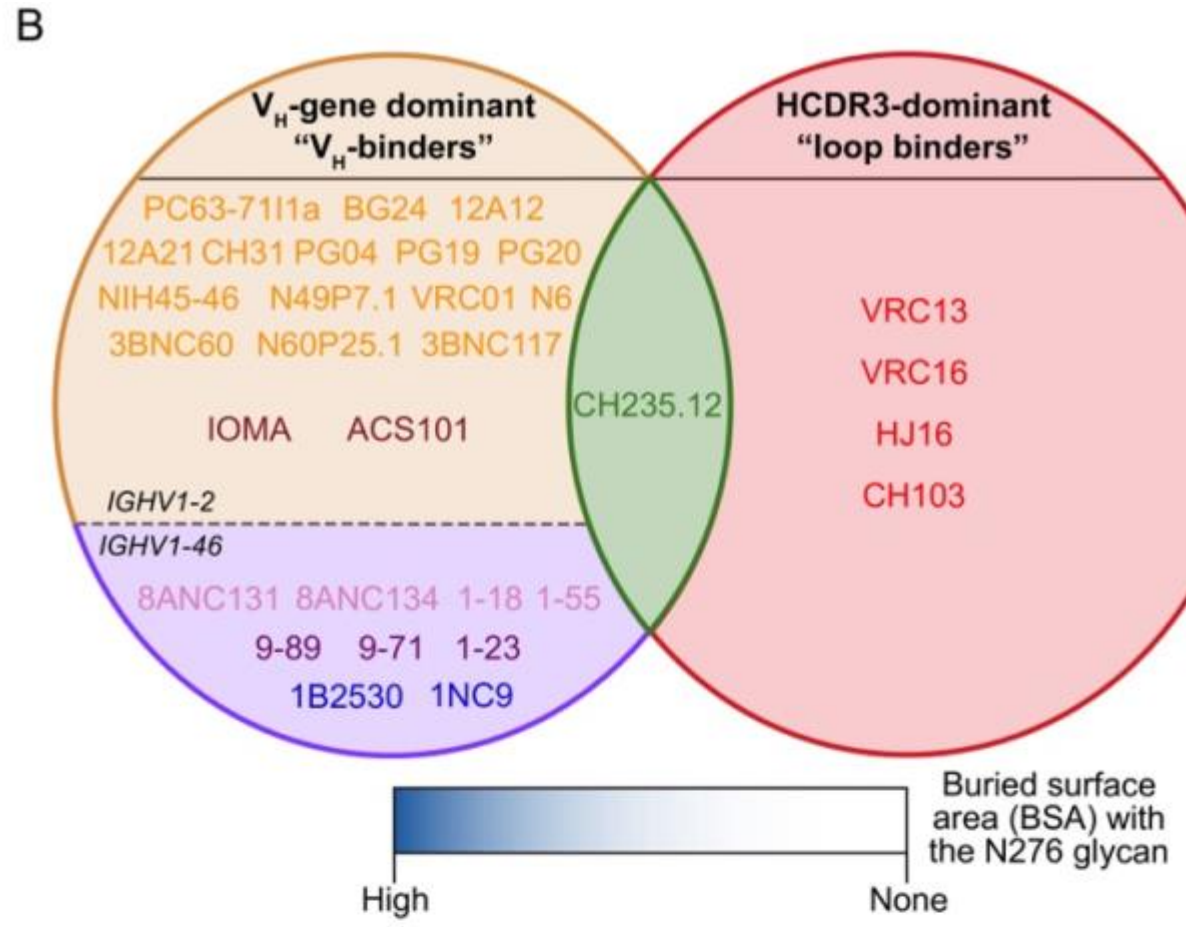

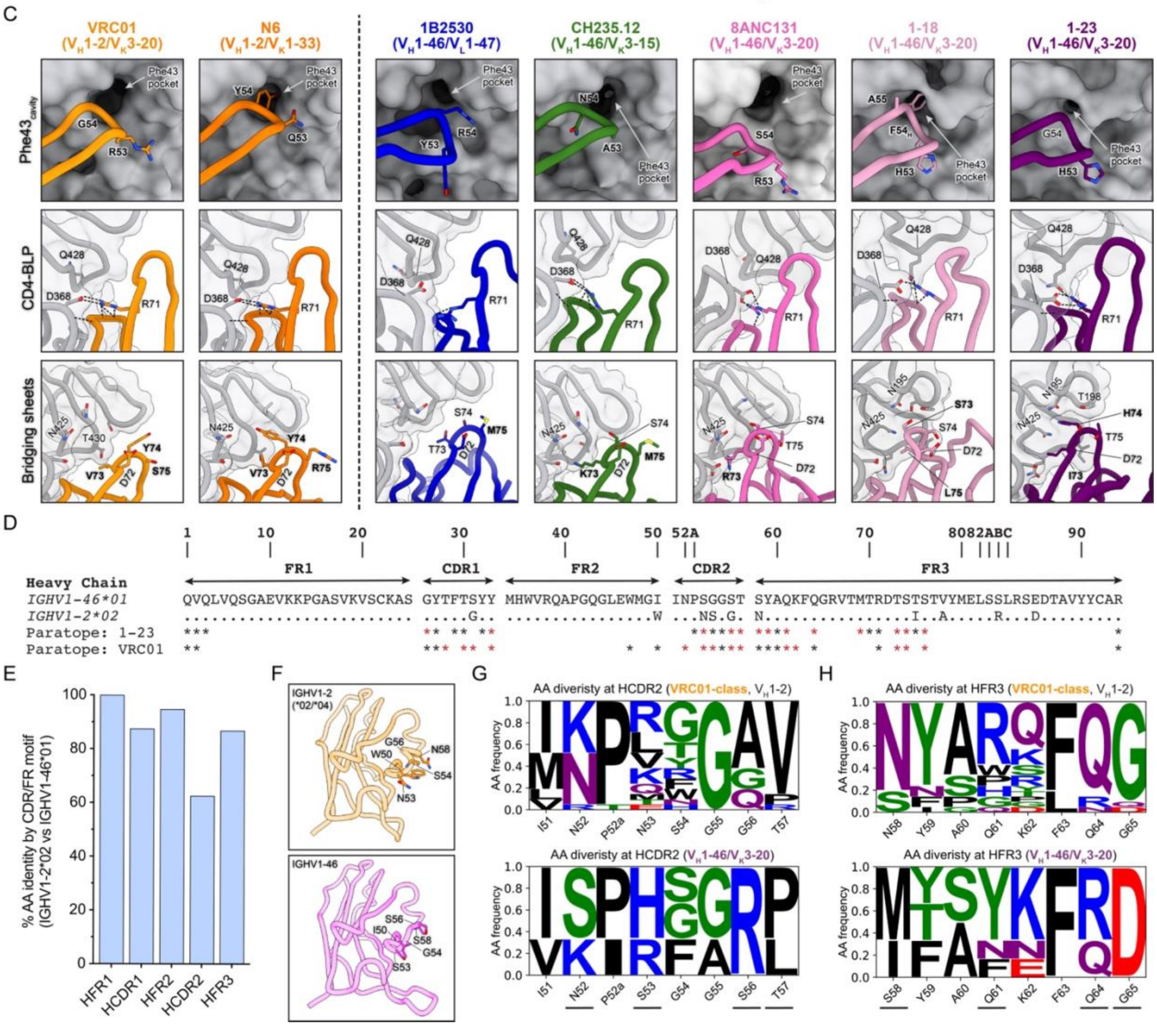

Figure S3. V_H1-46/V_K3-20-derived bnAbs exhibit distinctive epitope footprints and conserved heavy chain affinity maturation. Related to Figure 2.

(A) Heavy and light chain interface buried surface area (BSA) for representative VRC01-class bnAbs VRC01 (PDB:5FYJ), N6 (PDB:5TE6); IOMA-class bnAbs IOMA (PDB:5T3Z) and ACS101 (PDB:7Z3A); V_H1-46-derived bnAbs 1B2530 (PDB:4YFL), CH235.12 (PDB:8SXJ), 8ANC131 (PDB:4RWY), 8ANC134 (PDB: 4RX4) 1-18 (PDB:6UDJ), 1-55 (PDB:6UDK), 1-23 and 9-71; and HCDR3-dependent bnAbs CH103 (PDB:9D7O), HJ16 (PDB:4YE4), VRC13 (PDB:4YDJ), and VRC16 (PDB:4YDK) interacting with gp120$_A$, gp120$_B$ (adjacent protomer) and the N276 glycan. BSA data shown as NA and highlighted with an asterisk (*) indicate Fab-gp120 complex structures where the adjacent gp120$_B$ protomer was absent or the N276 glycan was not modeled. Blue gradient intensity represents relative BSA among bnAbs (white = lowest, dark blue = highest).

(B) Schematic comparing V_H-gene-dependent (V_H1-2, orange; V_H1-46, purple) from HCDR3-dependent (red) bnAbs. V_H-dependent bnAbs show pronounced N276 glycan contacts and rely primarily on HCDR2/HFR3 for paratope BSA, while HCDR3-dependent bnAbs avoid N276 glycan entirely and depend on HCDR3 for paratope BSA. CH235.12 (green) represents a hybrid mechanism combining both strategies. Blue gradient scale bar indicates relative N276 glycan BSA (white = no interaction, dark blue = highest interaction). Related to Figure S3A and Table S5C.

(C) Key interactions of V_H1-2-dependent bnAbs VRC01 (PDB:3NGB) and N6 (PDB:5TE6), and V_H1-46-dependent bnAbs 1B2530 (PDB:4YFL), CH235.12 (PDB:5F96), 8ANC131 (PDB:4RWY), 1-18 (PDB:6UDJ), and 1-23 with HIV-1 gp120 (light grey) at three CD4-binding site regions: Phe43 cavity, CD4-binding loop (D368), and the bridging β-sheets. Expected hydrogen bonds and salt bridges using a cutoff distance of 3.2 Å are shown as dotted lines. Critical interactions formed by somatically mutated heavy chain residues are highlighted in bolt.

(D) Amino acid sequence alignment of IGHV1-2*02 and IGHV1-46*01 germline genes revealing high sequence diversity at critical paratope regions. Heavy chain paratope residues are marked with asterisks (*), and residues present in minimally mutated antibodies are highlighted in red.

(E) Amino acid identity (%) by CDR and FR region between IGHV1-2*02 and IGHV1-46*01 germline genes, showing highest sequence diversity in the HCDR2 loop.

(F) HCDR2 structural diversity between germline genes: IGHV1-2*02/04 (orange, VRC01c-HuGL2 Fab/eOD-GT8, PDB:5IF0) versus IGHV1-46 (purple, CH235-UCA/Man5-enriched CH505.N279K.G458Y SOSIP, PDB:6UDA).

(G) Amino acid frequency at key HCDR2 residues comparing VRC01-class bnAbs with six V_H1-46/V_K3-20-paired bnAbs (1-18, 1-55, 1-23, 9-71, 8ANC131, 8ANC134). X-axis labels show germline residues for each IGHV gene type (V_H1-2, top; V_H1-46, bottom). Black horizontal line underscores SHMs conserved among V_H1-46/V_K3-20-paired bnAbs.

(H) Amino acid frequency at key HFR3 residues comparing VRC01-class bnAbs (VRC01, N6, PG04, VRC03, CH31, 3BNC117, 3BNC60, VRC27, 12A21, 12A12, N49P7.1, NIH45-46, VRC07,VRC08, VRC18, PG20, VRC23, PG19, N60P25.1, BG24, PCIN63-71I1a) with six V_H1-46/V_K3-20-paired bnAbs (1-18, 1-55, 1-23, 9-71, 8ANC131, 8ANC134). X-axis labels show germline residues for each IGHV gene type (V_H1-2,

top; V_H1-46, bottom). Black horizontal line underscores SHMs conserved among V_H1-46/V_K3-20-paired bnAbs.

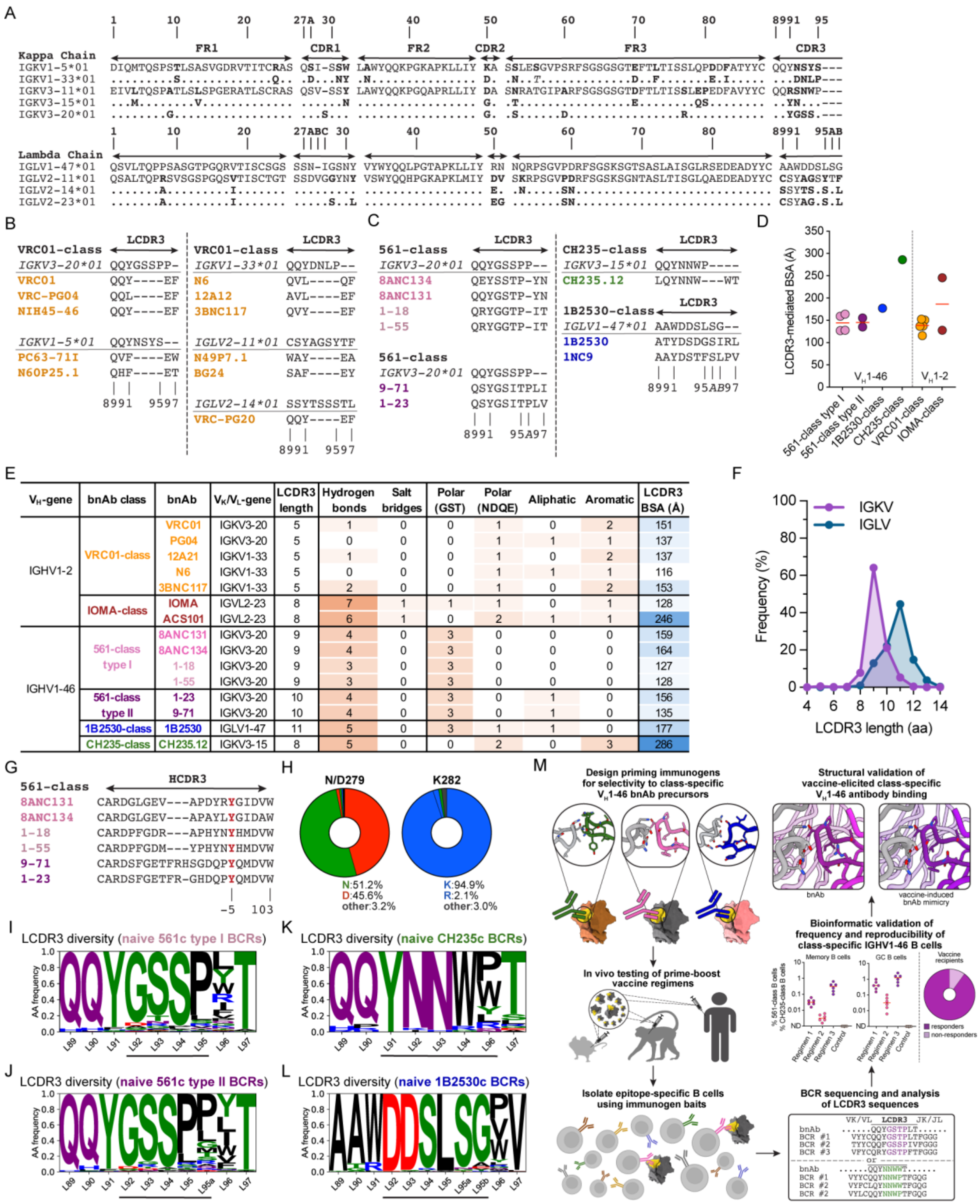

Figure S4. Light chain pairing dictates V_H1-46 bnAb signatures and precursor frequencies. Related to Figure 3.

(A) Sequence alignment of diverse IGKV and IGLV germline genes employed by V_H1-2 and V_H1-46-dependent bnAbs, showing the sequence diversity in LCDR3 region. Bold letters highlight amino acid position with diversity within the same gene family.

(B) LCDR3 sequence alignments for diverse VRC01-class (orange) bnAbs. Sequences are shown relative to germline V_K/V_L sequences.

(C) LCDR3 sequence alignments of three different classes of V_H1-46 bnAbs, compared to their respective germline V_K/V_L sequences.

(D) Dot plot visualizing the LCDR3-mediated binding surface area (BSA) on gp120 CD4bs-targeting bnAbs described in (E).

(E) Summary table of V_K/V_L-gene usage, LCDR3 properties (length, paratope chemistries), and LCDR3-mediated gp120 interactions (hydrophilic contacts, binding surface area) for representative VRC01-class bnAbs VRC01 (PDB:4JPW), N6 (PDB:5TE6), 12A21 (PDB:4JPW), PG04 (PDB:3SE9), 3BNC117 (PDB:5V8M); IOMA-class bnAbs IOMA (PDB:5T3Z) and ACS101 (PDB:7Z3A); and V_H1-46-derived bnAbs 1B2530 (PDB:4YFL), CH235.12 (PDB:8SXJ), 8ANC131 (PDB:4RWY), 8ANC134 (PDB: 4RX4) 1-18 (PDB:6UDJ), 1-55 (PDB:6UDK), 1-23, and 9-71.

(F) LCDR3 length distribution in the human IGKV and IGLV repertoire as a frequency of total sequences.

(G) Alignment of 561-class type I and type II HCDR3 sequences highlighting conserved Y103-5 residue (red).

(H) Pie graphs of amino acid diversity at key loop D residues, D279 and K282, that form critical contacts with VRC01-class and 561-class bnAbs.

(I-L) LCDR3 sequence diversity observed in the naïve human B cell repertoire for putative 561-class type I (I), 561-class type II (J), CH235-class (K), 1B2530-class (L) precursor BCRs. Black lines indicate key bnAb paratope residues that contact gp120. Heavy and light chain residues are numbered according to the Kabat numbering scheme.

(M) Schematic highlighting key metrics for evaluating vaccine-elicited V_H1-46 antibody responses in pre-clinical and clinical germline-targeting vaccine regimens. Icons for IgGs, B cells, syringe and animal models were created with BioRender.com and can be found under (https://biorender.com/53plvya).

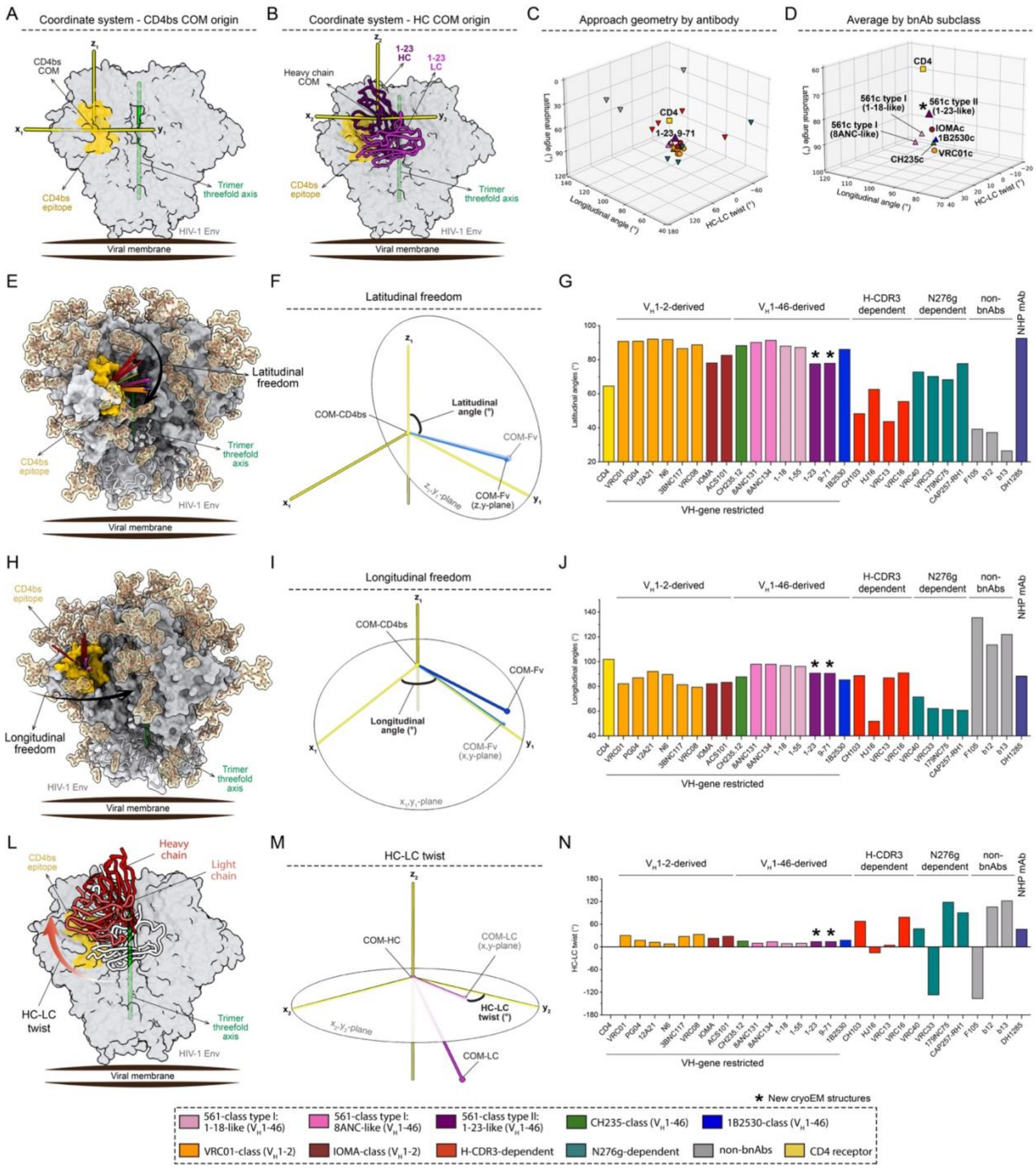

Figure S5. Approach geometries of CD4bs-targeting antibodies. Related to Figure 4.

(A) Three-dimensional coordinate system centered at the CD4bs center of mass (COM_{CD4bs}) with the z_1-axis parallel to the trimer threefold axis was used to measure latitudinal and longitudinal angles of approach. The coordinate system (yellow) is depicted spatially in regard to the HIV-1 trimer (grey), the CD4bs (orange), and the trimer threefold axis (green).

(B) Cartesian coordinate system centered at the heavy chain COM (COM_{HC}) the z_2-axis parallel to the trimer threefold axis was used to determine the HC-LC twist and HC-LC latitudinal angles (binding mode). The coordinate system (yellow) is depicted spatially in regard to the HIV-1 trimer (grey), the CD4bs (orange), the trimer threefold axis (green) and a CD4bs-targeting bnAb (purple).

(C) 3D scatter plot showing latitudinal, longitudinal, and HC-LC twist angles for all CD4bs-targeting antibodies and the CD4 receptor.

(D) 3D scatter plot showing average latitudinal, longitudinal, and HC-LC twist angles for diverse classes of V_H-gene dependent bnAbs and the CD4 receptor.

(E&H) Latitudinal and longitudinal angles of approach for CD4bs antibodies on the trimer. The HIV-1 trimer (gray) with N-linker glycans (beige) in stick and surface representation highlight the CD4bs (yellow) steric occlusion imposed by surrounding glycans and the adjacent protomer.

(F) Cartesian coordinate system defining the latitudinal angle of approach on the z_1-y_1 plane.

(G) Histogram depicting the variability in latitudinal approach angles across CD4bs-targeting bnAbs and human CD4. Stars (*) indicate new cryo-EM structures.

(I) Cartesian coordinate system defining the longitudinal angle of approach on the x_1-y_1 plane.

(J) Histogram showing the longitudinal approach angles adopted by CD4bs-targeting bnAbs and human CD4. Stars (*) indicate new cryo-EM structures.

(L) Structural schematic depicting HC-LC twist around the CD4bs epitope (yellow) from V_H-gene mediated (white) to HCDR3-mediated and non-neutralizing (red) twist angles.

(M) Cartesian coordinate system defining the HC-LC twist angle on the x_2-y_2 plane.

(N) Histogram highlighting V_H-dependent bnAbs adopt a conserved HC-LC twist; whilst other CD4bs-targeting antibodies exhibit greater HC-LC twist variability. Stars (*) indicate new cryo-EM structures.

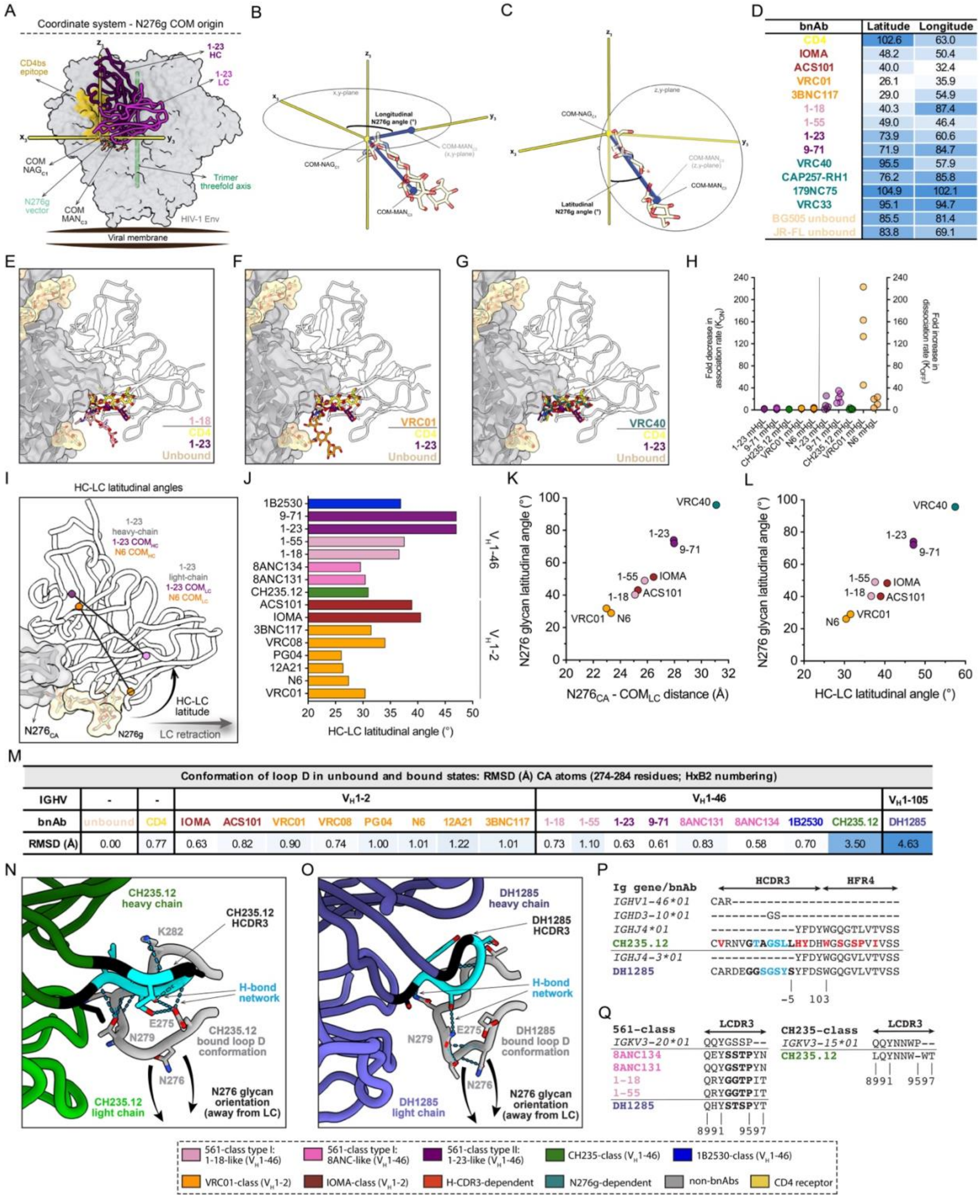

Figure S6. Two distinct mechanisms enable V_H1-46 bnAbs to overcome N276 glycan barrier. Related to Figure 5.

(A) Three-dimensional coordinate system centered at the N276 glycan NAG_{C1} center of mass (COM), with a vector from COM-NAG_{C1} to COM-MAN_{C3} defining the orientation of the first three sugars at the base of

the N276 glycan tree. The coordinate system (yellow) is depicted spatially with regard to the HIV-1 trimer (grey), CD4bs (orange), and a CD4bs-targeting bnAb (purple).

(B-C) Cartesian coordinate system defining N276 glycan orientation angles. Longitudinal angles are measured in the x-y plane and latitudinal angles in the y-z plane. Blue vectors between COM-NAG_{C1} (yellow) and COM-MAN_{C3} (blue) centroids define glycan tree (beige) directionality relative to the CD4-binding site epitope.

(D) N276 glycan longitudinal and latitudinal angles upon binding by CD4 receptor (PDB:5VN3,yellow), IOMA-class bnAbs [IOMA (PDB:5T3Z) and ACS101 (PDB:7Z3A)], VRC01-class bnAbs [VRC01 (PDB:5FYJ) and 3BNC117 (PDB:5V8M)], 561-class type-I [1-18 (PDB:6UDJ) and 1-55 (PDB:6UDK)], and 561-class type-II [1-23 and 9-71], N276g-dependent bnAbs [VRC40 (PDB:7LL1), CAP257-RH1 (PDB:5T33), 179NC75 (PDB:7LLK), and VRC33 (PDB:7LL2)], compared to the unbound native conformation [BG505 (PDB:5FYL) and JR-FL (PDB:5FUU)].

(E-G) Cartoon representation showing N276 glycan displacement and steric clashes with the light chain of CD4bs-targeting VRC01-class antibody (PBD:5FYJ, white). N276 glycan conformation upon binding to VRC01 (PDB:5FYJ, orange), 1-18 (PDB:6UDJ, pink), 1-23 (purple), VRC40 (PDB:7LL1, teal), and CD4 (PDB:5VN3, yellow) compared to unbound state (beige).

(H) The fold decrease in association rate (K_{ON}) and dissociation rate (K_{OFF}) upon reverting light chain somatic hypermutations to germline residues in CD4bs-targeting bnAbs.

(I) Structure schematic depicting the HC-LC latitudinal angles in CD4bs-targeting bnAbs. The glycan (beige surface) interaction with the 1-23 light chain (white) highlights its retracted positioning compared to the VRC01 light chain (PBD:5FYJ).

(J) The HC-LC latitudinal angle upon CD4bs engagement by V_H-gene dependent bnAbs: IOMA-class (brown), VRC01-class (orange), 561-class type-I (pink), 561-class type-II (purple), CH235-class (green) and 1B2530-class (blue).

(K) 2D-scatter plots showing the correlation between the N276 glycan latitudinal angle and the distance between the $N276_{CA}$ and the light chain of CD4bs-targeting antibodies.

(L) Correlation between the N276 glycan latitudinal angle and the HC-LC latitudinal angle in CD4bs-targeting bnAbs.

(M) Root mean square deviation (RMSD) of loop D $C\alpha$ atoms (residues 274-284, HxB2 numbering) upon binding to CD4bs bnAbs described in (D), CH235.12 (PDB:8SXJ), DH1285 (PDB:8U1D), and the CD4 receptor, compared to CD4bs-unbound conformation (PDB:5FYL).

(N) HCDR3-mediated interactions that stabilize the loop D in unnatural conformation upon CH235.12 (PDB:8SXJ) engagement. Hydrogen bonding residues and the H-bond network is colored in cyan.

(O) HCDR3-derived contacts that stabilize the loop D in unnatural conformation upon DH1285 (PDB:8U1D) binding. Hydrogen bonding residues and the H-bond network is colored in cyan.

(P) HCDR3 sequences of CH235.12 and DH1285 with loop D interacting (black) and hydrogen bonding (cyan) residues highlighted.

(Q) Sequence alignment of LCDR3 sequences showing shared immunogenetic signatures between DH1285 and 561-class antibodies.

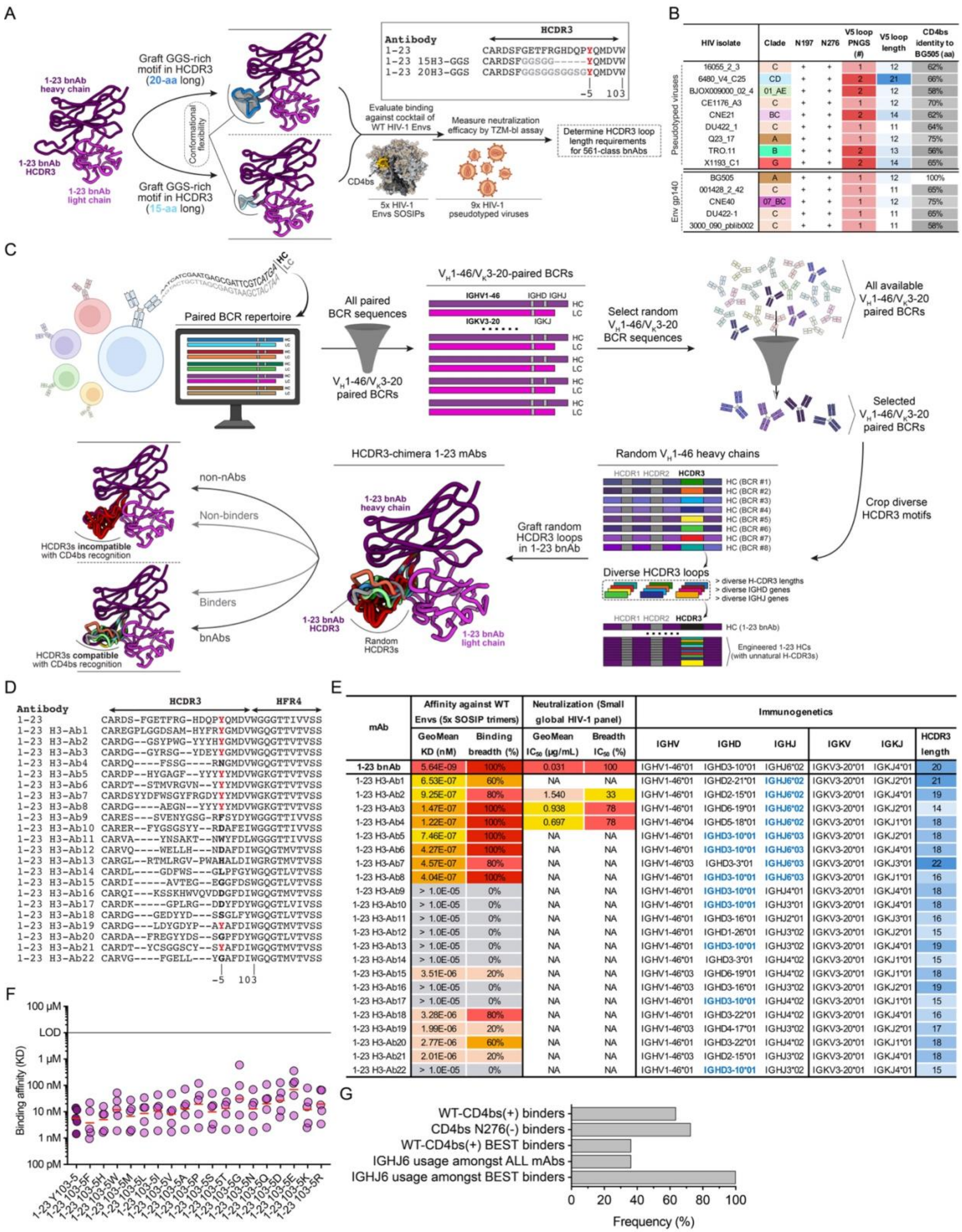

Figure S7. CD4bs recognition by 561-class bnAbs is V_H-gene dependent. Related to Figure 6.

(A) Schema illustrating strategy to probe HCDR3 length requirements for HIV-1 neutralization by 561-class bnAbs. 15- and 20-residue long HCDR3 variants of 1-23 bnAbs were generated by replacing the native HCDR3 sequence by GGS-rich motifs. Engineered antibodies were then evaluated for their capability to bind WT HIV-1 trimers and neutralize HIV-1 pseudo-typed viruses.
(B) HIV-1 isolates included in the Env panels, represented by five soluble gp140 SOSIPs and nine pseudotyped viruses. Shown are clade assignment, presence of absence of the N276 and N197 glycans, V5 loop length, number of predicted N-glycans within the V5 loop, and percent amino acid identity at the CD4-binding site relative to BG505.
(C) Schematic diagram of the strategy to reproduce HCDR3 structural and sequence diversity from the human B cell repertoire. Random IGHV1-46/IGKV3-20-paired sequences from the OAS database were selected, and their HCDR3 loops were grafted onto 1-23 bnAb to generate twenty-two unique variants evaluated for CD4bs recognition and HIV-1 neutralization. Icons for filter, monitor, B cells and antibodies used to generate schematic diagram were created with BioRender.com and can be found under Bader, D. (2026) (https://BioRender.com/bjmsm12).
(D) Amino acid sequence of the twenty-two selected HCDR3 variants aligned to the 1-23 bnAb HCDR3 sequence. The Tyr103-5 residue is highlighted in bold (red).
(E) Summary table of selected IGHV1-46/IGKV3-20-paried BCRs showing HCDR3 length, immunoglobulin gene usage, and the binding affinity and neutralization capacity of corresponding 1-23 HCDR3 chimeric variants. White cells (NA) indicate untested antibodies. Values are colored according to the legend.
(F) Dot plot visualizing the binding affinity of 1-23 bnAb (purple) and single point mutant mAbs (light purple) against a panel of five wild-type stabilized trimers. The geometric mean affinity for each antibody is displayed by a red line.
(G) Histogram displaying the percentage of 1-23 HCDR3 variants that bind WT HIV-1 Env trimers, bind both WT and N276-knockout trimers, or represent best binders (binding all five WT stabilized trimers), along with the IGHJ6 gene usage frequency amongst all selected variants and best binders.

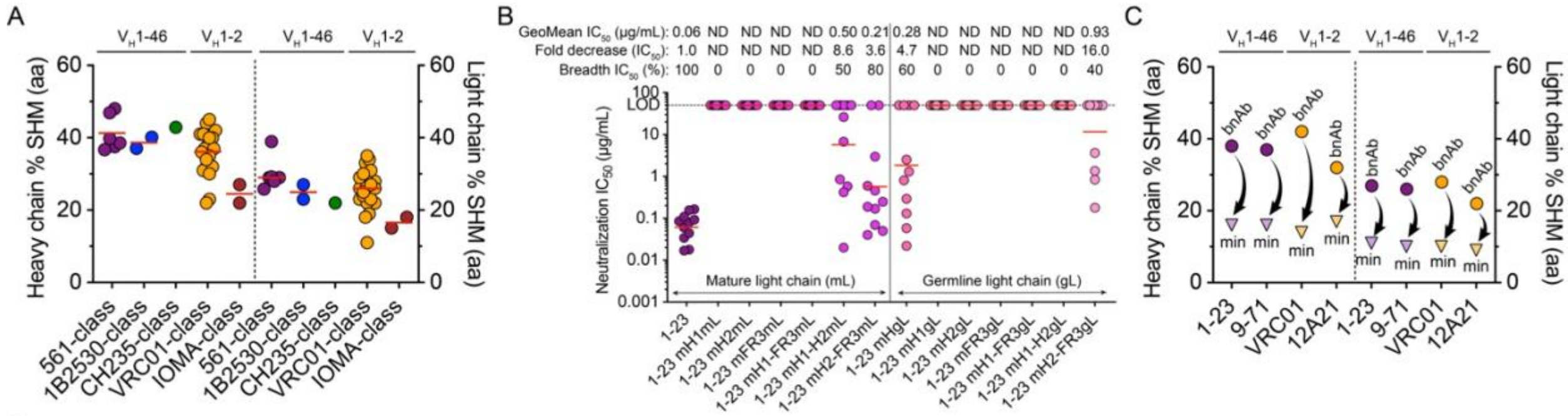

D

Multiclade AMP panel	CD4-binding site								V3-glycan		MPER	
	12A21	min12A21	VRC01	minVRC01	1-23	min1-23	9-71	min9-71	BG18	minBG18.11	10E8	min10E8
# PVs tested	23	23	23	23	23	23	23	23	23	23	23	23
Breadth IC_{50} (%)	59%	35%	91%	70%	91%	61%	91%	61%	61%	52%	87%	52%
Median IC_{50} (μg/mL)	0.196	0.396	0.446	0.395	0.137	5.630	0.307	6.480	0.022	0.685	0.903	5.703
GeoMean IC_{50} (μg/mL)	0.185	0.579	0.442	0.328	0.134	3.600	0.374	4.075	0.027	0.567	0.720	3.676
Fold difference (Median IC_{50})	1.0	2.0	1.0	0.9	1.0	12.6	1.0	21.1	1.0	30.8	1.0	6.3
Fold difference (GeoMean IC_{50})	1.0	3.1	1.0	0.7	1.0	8.1	1.0	10.9	1.0	20.9	1.0	5.1

GeoMean IC_{50} (μg/mL)	Breadth (%)
> 1.000	< 24.9%
0.990-0.100	25.0-49.9%
0.099-0.010	50.0-74.9%
< 0.0099	> 75.0%

E

Multiclade AMP panel	Mature bnAbs								Minimally mutated bnAbs					
Epitope	V2 apex						V3-glycan	FP	CD4-binding site				V3-glycan	MPER
Antibody	PGT145	PG9	PG16	CH01	PCT64.18D	PCT64.35S	PGT130	VRC34	min12A21	minVRC01	min1-23	min9-71	minBG18.11	min10E8
# PVs tested	23	23	23	23	23	23	23	23	23	23	23	23	23	23
Breadth IC_{50} (%)	57%	57%	43%	26%	26%	22%	35%	61%	35%	70%	61%	61%	52%	52%
Median IC_{50} (μg/mL)	6.687	0.982	0.040	24.608	3.647	6.129	0.148	0.460	0.396	0.395	5.630	6.480	0.685	5.703
GeoMean IC_{50} (μg/mL)	3.160	1.068	0.057	17.783	2.117	2.691	0.159	0.688	0.579	0.328	3.600	4.075	0.567	3.676

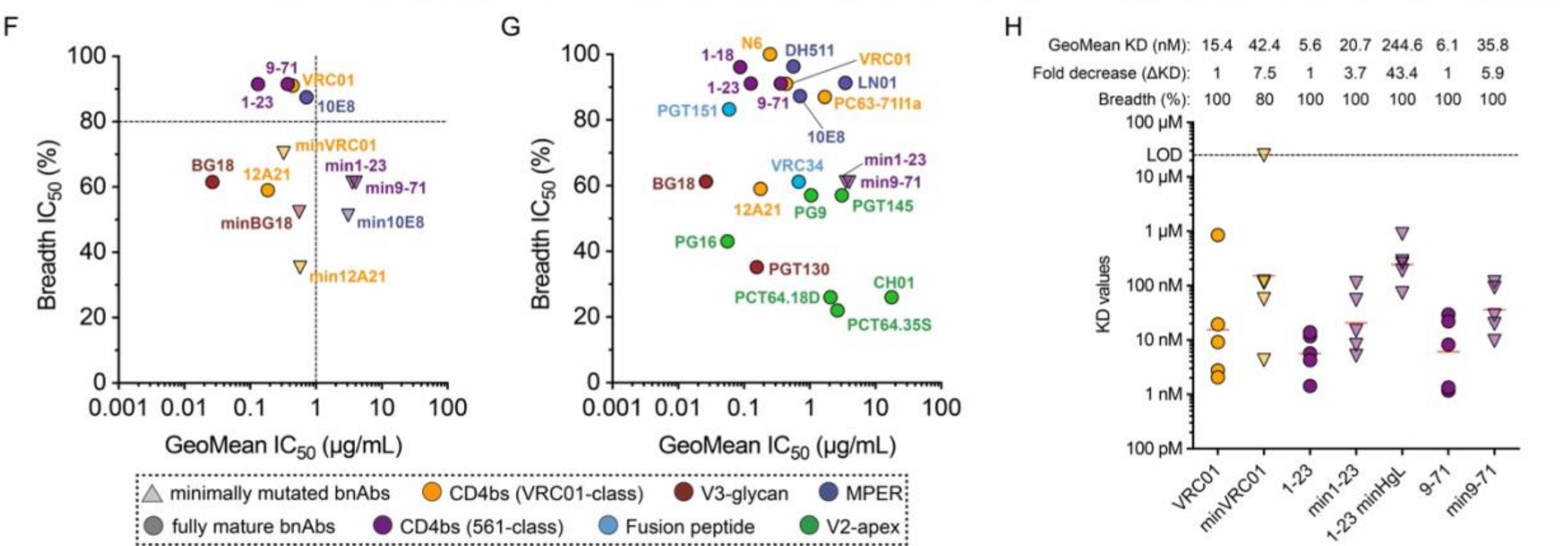

I

Effect of SHM reversion	ΔKon (fold decrease)				ΔKoff (fold increase)				ΔKD (fold decrease)			
	min1-23	1-23 minHgL	min9-71	minVRC01	min1-23	1-23 minHgL	min9-71	minVRC01	min1-23	1-23 minHgL	min9-71	minVRC01
1428	1.2	2.6	1.5	1.2	1.0	9.2	2.4	1.3	1.3	24.1	3.4	1.5
3000	1.6	2.0	1.5	1.0	12.1	78.8	2.7	12.0	19.8	156.4	3.9	12.3
DU422-1	1.5	1.3	2.2	-	2.6	13.6	1.9	-	3.9	18.2	4.2	-
BG505	1.8	2.4	1.4	2.4	0.7	7.0	5.9	11.5	1.2	17.0	8.3	27.3
CNE40	1.5	3.3	1.5	1.2	3.8	39.9	9.5	4.9	5.7	131.7	14.6	6.1
BG505 WT	1.8	2.4	1.4	2.4	0.7	7.0	5.9	11.5	1.2	17.0	8.3	27.3
BG505 N197Q	1.8	3.8	1.1	2.5	0.6	1.9	3.1	11.7	1.0	7.3	3.4	29.0
BG505 N276Q	2.4	3.5	2.8	2.1	0.5	1.7	3.0	0.9	1.3	5.9	8.5	1.9
BG505 N462Q	1.4	2.2	1.4	2.1	0.3	5.6	5.7	8.4	0.4	12.2	7.8	18.0

Coloring scale	Effect on affinity
min value	increase
<1.0	
1	none
>1.0	decrease
max value	

Figure S8. Minimally mutated 561-class bnAbs guide reductionist vaccine design. Related to Figure 7.

(A) Dot plot showing the number of amino acid mutations in heavy chain (right) and light chain (left) in 561-class (purple), 1B2530-class (blue), CH235-class (green), VRC01-class (orange), and IOMA-class (brown) antibodies.

(B) Neutralization breadth and potency of 1-23 variants with heavy chain CDR/FR patches mutated from germline paired with mature (mL; dark pink) and germline-reverted (gL; light pink) light chains against a 10-

virus panel of multi-clade HIV-1 isolates. Geometric mean IC_{50} values are shown as red horizontal lines and labeled above the graph, along with fold decrease in potency and neutralization breadth.

(C) Dot plot depicting the number of amino acid mutations in heavy chain (right) and light chain (left) in mature bnAbs (dark tone) and minimally mutated variants (light tone).

(E) Neutralization breadth and potency of bnAbs and corresponding minimally mutated antibodies targeting the CD4bs (VRC01, 1-23, and 9-71), the V3 glycan supersite (BG18), and the MPER epitope (10E8) against a clade B & C 23-virus panel of AMP-derived HIV-1 isolates. Values are colored according to the legend, and blue gradient intensity represents fold reduction in geometric mean and median IC_{50} values (white = no change, dark blue = maximum reduction).

(F) Neutralization breadth and potency of minimally mutated antibodies targeting the CD4bs (VRC01, 1-23, and 9-71), the V3 glycan supersite (BG18), and the MPER epitope (10E8) compared to mature bnAbs targeting the V2 apex (PGT145, PG9, PG16, CH01, PCT64.18D, PCT64.35S), the V3 glycan (PGT130) and the fusion peptide (VRC34) against a clade B & C 23-virus panel of AMP-derived HIV-1 isolates.

(G) Scatter plot illustrating the neutralization breadth and potency of bnAbs (circles) and corresponding minimally mutated (triangles) antibodies described in summary table (S7E).

(H) Neutralization breadth and potency scatter plot corresponding to summary table (S7F).

(I) Dot plot visualizing the binding affinity of mature minimally mutated variants of 561-class (purple) and VRC01-class (orange) antibodies against a panel of five wild-type stabilized trimers. The binding breadth, geometric mean affinity and average fold reduction in affinity are highlighted on top of the graph.

(J) The fold decrease in association rate (K_{ON}) versus dissociation rate (K_{OFF}) and their effect on overall changes in binding affinity (K_D) against native-like Env trimers upon reverting >50% of mutations to germline residues in 561-class (purple) and VRC01-class (orange) antibodies.

Table S2. Comprehensive list of CD4bs bnAbs with their immunogenetic signatures and neutralization breadth. Related to Figure 1 & 7.

bnAb type	bnAb class	CD4bs bnAb	VH gene	DH gene	JH gene	VK/VL gene	JK/JL gene	LCDR3 length	HCDR3 length	Indels HC {region}	Indels LC {region}	VH %SHM (aa)	VK/VL %SHM (aa)	Breadth at IC50 <50μg/mL {# PVs tested}	Ref.
HCDR3-dependent	CH103-class	**CH103**	VH4-59*01			VL3-1*01		10	13	-	-			80% {285}	34
	HJ16-class	**HJ16**	VH3-30*01			VK4-1*01		8	19	-	-			31% {391}	35
	VRC13-class	**VRC13**	VH1-69*01			VL2-14*01		6	21	-	-			80% {433}	24
	VRC16-class	**VRC16**	VH2-23*01			VK1-39*01		9	20	-	-			56% {241}	24
V_H1-2-derived	IOMA-class	**IOMA**	VH1-2*01			VL2-23*02		9	19	-	-	22%	15%	52% {244}	76
		**ACS101**	VH1-2*01	DH1-26*01	JH5*02	VL2-23*02		9	19	-	Δ2-aa {L1}	27%	18%	39% {143}	47
	VRC01-class	**N6**	VH1-2*02	DH3-10*01	JH5*02	VK1-33*01	JK5*01	5	15	-	-	41%	33%	98% {520}	33
		**VRC01**	VH1-2*02	DH3-16*01	JH1*01	VK3-20*01	JK2*01	5	14	-	Δ3-aa {L1}	42%	28%	87% {1836}	19
		**VRC03**	VH1-2*02	DH2-15*01	JH1*01	VK3-20*01	JK2*01	5	23	7-aa {FR3}	Δ2-aa {L1}	38%	30%	57% {474}	19
		**VRC07**	VH1-2*02	DH2-8*02	JH1*01	VK3-20*01	JK2*01	5	18	-	Δ3-aa {L1}	44%	26%	91% {400}	6
		**VRC08**	VH1-2*02	DH2-15*01	JH1*01	VK3-20*01	JK2*01	5	25	-	-	37%	34%	90% {202}	48
		**VRC18**	VH1-2*02	DH3-3*02	JH4*02	VK3-20*01		5	12	2-aa {FR3}	-	37%	23%	67% {174}	24
		**VRC23**	VH1-2*02			VK3-15*01	JK2*01	5	9	-	-	32%	26%	64% {180}	37
		**VRC27**	VH1-2*02			VK1-33*01	JK5*01	5	10	-	-	40%	35%	78% {178}	24
		**VRC-PG04**	VH1-2*02	DH6-25*01	JH2*01	VK3-20*01	JK5*01	5	16	1-aa {H1&H2}	Δ3-aa {L1}	45%	31%	81% {447}	22
		**VRC-PG19**	VH1-2*02	DH3-3*01	JH1*01	VL2-14*03	JL2*01	5	13	-	Δ1-aa {L1}	31%	22%	75% {20}	23
		**VRC-PG20**	VH1-2*02	DH1-26*01	JH1*01	VL2-14*03	JK5*01	5	15	-	Δ6-aa {L1}	37%	11%	77% {182}	23
		**VRC-CH31**	VH1-2*02	DH3-16*01	JH1*01	VK1-33*01	JK2*01	5	15	9-aa {H1}	-	41%	22%	81% {454}	22
		**12A12**	VH1-2*02	DH1-26*01	JH5*02	VK1-33*01	JK1*01	5	15	-	-	35%	24%	95% {237}	21
		**12A21**	VH1-2*02	DH1-26*01	JH5*02	VK1-33*01	JK3*01	5	15	-	-	32%	23%	73% {199}	21
		**3BNC117**	VH1-2*02	DH6-25*01	JH2*01	VK1-33*01	JK3*01	5	12	4-aa {FR3}	Δ4-aa {L1}	36%	27%	87% {1237}	21
		**BG24**	VH1-2*02	DH1-1*01	JH3*02	VL2-11*01	JL2*01	5	16	-	Δ6-aa {L1}	23%	25%	83% {139}	39
		**PC63-71I**	VH1-2*02	DH5-24*01	JH5*02	VK1-5*01	JK1*01	5	15	-	-	22%	19%	87% {135}	41
		**NIH45-46**	VH1-2*02	DH2-8*02	JH1*01	VK3-20*01	JK2*01	5	18	-	Δ3-aa {L1}	41%	27%	82% {368}	21
		**N49P7.1**	VH1-2*02	DH3-10*01	JH2*01	VL2-11*01	JL2*01	5	21	-	Δ6-aa {L1}	34%	29%	99% {117}	38
		**N60P25.1**	VH1-2*02	DH5-24*01	JH5*02	VK1-5*01	JK1*01	5	13	-	Δ2-aa {L1}	30%	18%	74% {71}	38
		**3BNC60**	VH1-2*02	DH3-3*01	JH2*01	VK1-33*01	JK3*01	5	12	4-aa {FR3}	Δ4-aa {L1}	40%	30%	88% {51}	21
V_H1-46-derived	1B2530-class	**1B2530**	VH1-46*01	DH6-13*01	JH5*02	VL1-47*01	JL3*02	11	18	-	-	40%	23%	57% {225}	21
		**1NC9**	VH1-46*01	DH5-24*01	JH4*02	VL1-47*01	JL2*01	11	21	-	-	37%	27%	77% {71}	21
	CH235-class	**CH235.12**	VH1-46*01	DH6-13*01	JH4*02	VK3-15*01	JK1*01	8	15	-	-	43%	22%	81% {434}	25
	561-class	**1-18**	VH1-46*01	DH3-10*01	JH6*03	VK3-20*01	JK4*01	9	18	6aa {H1}	-	48%	29%	94% {435}	26
		**9-89**	VH1-46*01	DH5-24*01	JH6*03	VK3-20*01	JK4*01	10	18	Δ2aa {FR1}	2aa {FR1}	47%	39%	92% {12}	26
		**9-71**	VH1-46*01	DH3-10*01	JH6*02	VK3-20*01	JK4*01	10	21	Δ2aa {FR1}	-	38%	26%	83% {12}	26
		**1-23**	VH1-46*01	DH3-10*01	JH6*02	VK3-20*01	JK4*01	10	20	Δ2aa {FR1}	-	39%	28%	92% {12}	26
		**8ANC131**	VH1-46*01	DH3-16*02	JH6*01	VK3-20*01	JK3*01	9	18	Δ1aa {H1}	Δ2aa {L1}	40%	29%	74% {191}	21
		**8ANC134**	VH1-46*01	DH3-16*02	JH6*01	VK3-20*01	JK3*01	9	18	Δ1aa {H1}	Δ2aa {L1}	37%	29%	51% {173}	21

Table S5. Cryo-EM data collection, refinement and validation statistics. Related to Figure 2.

	BG505 MD39 SOSIP RM19R Fab **1-23 Fab**	BG505 MD39 SOSIP RM19R Fab **9-71 Fab**
**PDB ID**	9PIT	9PIV
**EMDB ID**	71677	71678
**Data collection and processing**		
Microscope	TFS Titan Krios	TFS Titan Krios
Magnification	130,000x	130,000x
Voltage (kV)	300	300
Electron exposure ($e^-/Å^2$)	50	50
Exposure rate (e^-/pixel/sec)	7.78	7.78
Number of EER frames	35	35
Defocus range (µm)	-0.8 to -1.6	-0.8 to -1.4
Detector	Gatan K2 Summit	Gatan K2 Summit
Recording mode	Counting	Counting
Automation Software	Leginon	Leginon
Pixel size (Å)	1.045	1.045
Symmetry imposed	C3	C3
Micrographs collected (no.)	4,808	5,300
Micrographs used (no.)	4,136	3,006
Initial particle images (no.)	958,003	1,052,718
Final particle images (no.)	500,071	230.472
Map resolution (masked/unmasked Å)	3.1/3.7	3.5/4.1
FSC threshold	0.143	0.143
Map sharpening *B* factor ($Å^2$)	-129	-134
Map pixel size (Å)	1.045	1.045
Map resolution range (Å)	2.7-5.0	3.0-5.5
**Refinement**		
Initial model used (PDB code)	6VKN	6VKN
Refinement package	Rosetta Relax & Phenix real space refine	Rosetta Relax & Phenix real space refine
Model resolution (Å)	3.2	3.6
FSC threshold	0.5	0.5
Model resolution range (Å)	2.7-3.9	3.0-4.4
EMRinger score	3.63	2.83
CC (mask)	0.83	0.79
Model composition		
Non-hydrogen atoms	25,533	25,338
Protein residues	3,075	3,069
Ligands (glycans)	105	96
Mean B factors ($Å^2$)		
Protein	78	84
Ligand	113	90
R.m.s.d. deviations		
Bond lengths (Å)	0.004	0.007
Bond angles (°)	0.986	1.108
Validation		
MolProbity score	1.13	1.26
Clashscore	2.73	3.37
Poor rotamers (%)	0.00	0.11
Ramachandran plot		
Favored (%)	97.68	97.31
Allowed (%)	2.22	2.69
Disallowed (%)	0.10	0.00
Cβ outliers (%)	0.00	0.00
CaBLAM outliers (%)	3.27	1.72

Table S6. Buried surface area (BSA) contributions of CD4bs bnAbs to critical CD4bs epitope regions. Related to Figure 2.

Table S6A: Buried surface area (BSA) contributions of VRC01-class bnAbs [VRC01 (PDB:5FYJ) and N6 (PDB:5TE6)], 561-class type-**I** bnAbs [8ANC131 (PDB:4RWY) and 1-18 (PDB:6UDJ)], 561-class type-**II** bnAbs [1-23 and 9-71], 1B2530 (PDB:4YFL), CH235.12 (PDB:8SXJ), DH1285 (PDB:8U1D), and CD4 receptor (PBD:5VN3) to critical CD4bs epitope regions. Blue gradient intensity indicates relative BSA contribution of overall bnAb epitope (white = lowest, dark blue = highest).

CD4-binding site epitope regions	V_H1-2-derived bnAbs		V_H1-46-derived bnAbs						DH1285	CD4 receptor	CD4bs residues (HxB2)
	VRC01-class		CH235-class	1B2530-class	561-class						
	VRC01	N6	CH235.12	1B2530	8ANC131	1-18	1-23	9-71			
C1-α0, C2-loop (adjacent protomer)	102	NA	61	NA	NA	100	166	106	83	31	60-66, 205-208
V3 loop (adjacent protomer)	0	0	0	0	0	175	0	0	0	0	301-324
Inner domain (C1-α1)	29	21	33	43	112	116	47	127	0	0	96-106
Bridging sheets (β20/21-sheets, V2)	207	171	176	80	280	**305**	**299**	**308**	130	**354**	423-435, 194-199
Outer domain exit loop (α5)	101	45	35	67	145	158	90	98	**201**	128	471-483
CD4-binding loop	171	206	219	249	159	222	219	219	166	259	362-375
Loop D	314	**337**	**549**	244	**312**	255	229	257	168	156	274-284
Loop V5 (β23/24-sheets, V5)	**373**	233	308	**365**	190	213	149	162	56	200	451-470
**TOTAL**	1297	1012	1382	1047	1198	1543	1198	1276	802	1128	

Table S6B: Average BSA contribution of distinct CD4bs bnAb classes to critical CD4bs epitope regions. Blue gradient intensity indicates relative BSA contribution of overall bnAb epitope for respective bnAb classes (white = lowest, dark blue = highest).

CD4bs epitope regions	BSA contribution per bnAb class (Å)					
	VRC01-class	CH235-class	1B2530-class	561-class	DH1285	CD4 receptor
C1-α0, C2-loop (adjacent protomer)	102	61	NA	124	83	31
V3 loop (adjacent protomer)	0	0	0	44	0	0
Inner domain (C1-α1)	25	33	43	101	0	0
Bridging sheets (β20/21-sheets, V2)	189	176	80	**298**	130	**354**
Outer domain exit loop (α5)	73	35	67	123	**201**	128
CD4-binding loop	189	219	249	204	166	259
Loop D	**325**	**549**	244	263	168	156
Loop V5 (β23/24-sheets, V5)	303	308	**365**	178	56	200
**TOTAL**	1155	1382	1047	1304	802	1128

Relative BSA contribution

lowest — highest

Table S6C: Contribution of heavy- and light-chain CDR/FR regions to HIV-1 Env binding by VRC01-class bnAbs [VRC01 (PDB:5FYJ) and N6 (PDB:5TE6)], IOMA-class bnAbs [IOMA (PDB:5T3Z) and ACS101 (PDB:7Z3A)], 561-class type-I [8ANC131 (PDB:4RWY), 8ANC134 (PDB:4RX4), 1-18 (PDB:6UDJ), and 1-55 (PDB:6UDK)], 561-class type-II [1-23 and 9-71], 1B2530-class antibody [1B2530 (PDB:4YFL)], CH235-class antibody [CH235.12 (PDB:8SXJ)], and HCDR3-dependent bnAbs [CH103 (PDB:9D7O), HJ16 (PDB:4YE4), VRC13 (PDB:4YDJ), and VRC16 (PDB:4YDK)]. Blue gradient intensity indicates relative BSA contribution of overall bnAb paratope (white = no BSA, dark blue = highest). An asterisk (*) indicates complex structures resolved by X-ray crystallography with gp120; which have some missing paratope BSA values (NA).

Antibody	Heavy chain paratope with Env (Å)							Light chain paratope with Env (Å)						Light chain paratope with N276 (Å)					
	HFR1	HCDR1	HFR2	HCDR2	HFR3	HCDR3	Total	LFR1	LCDR1	LCDR2	LFR3	LCDR3	Total	LCDR1	LCDR2	LFR3	LCDR3	Total	
VRC01	40	82	64	313	442	146	1087	21	17	0	0	171	209	181	0	168	13	362	
N6	NA	16	59	343	330	156	904	73	16	0	0	119	208	56	0	NA	16	72	*
ACS101	0	17	23	218	362	322	943	79	25	0	0	230	334	134	0	0	0	134	
IOMA	0	3	10	338	332	188	871	0	82	0	0	158	240	142	0	19	0	162	
1B2530	NA	2	63	315	334	99	813	83	0	0	0	227	310	NA	NA	NA	NA	NA	*
CH235.12	79	5	11	268	327	335	1025	26	110	0	0	210	346	0	0	0	0	0	
8ANC131	NA	0	2	343	368	218	931	42	19	0	0	158	219	74	0	NA	20	93	*
8ANC134	NA	117	0	346	360	259	1082	0	18	1	0	164	183	75	0	NA	16	91	*
1-18	0	322	0	432	349	234	1338	31	9	0	0	133	173	238	0	116	20	374	
1-55	0	345	0	443	370	268	1426	21	2	0	0	137	160	152	0	11	0	162	
1-23	24	139	0	327	428	142	1059	0	5	0	0	157	162	148	45	103	35	330	
9-71	0	104	0	316	457	258	1134	10	31	0	0	141	182	188	6	85	36	316	
CH103	0	62	19	251	274	377	983	0	49	144	10	76	279	0	0	0	0	0	
HJ16	NA	31	0	126	0	457	614	6	149	0	0	209	364	52	0	0	0	52	*
VRC13	NA	343	0	21	7	587	958	0	10	14	0	0	25	0	0	0	0	0	*
VRC16	NA	0	0	161	109	675	944	0	103	41	41	112	296	0	0	0	0	0	*

Table S7. Quantitative analysis of approach angles and binding modes in CD4bs recognition. Related to Figure 4.

Approach geometry to the CD4bs by VRC01-class bnAbs [VRC01 (PDB:5FYJ), PG04 (PDB:3SE9), 12A21 (PDB:4JPW), 3BNC117 (PDB:5V8M), VRC08 (PDB:4XMP) and N6 (PDB:5TE6)], IOMA-class bnAbs [IOMA (PDB:5T3Z) and ACS101 (PDB:7Z3A)], 561-class type-I [8ANC131 (PDB:4RWY), 8ANC134 (PDB:4RX4), 1-18 (PDB:6UDJ), and 1-55 (PDB:6UDK)], 561-class type-II [1-23 and 9-71], 1B2530-class antibody [1B2530 (PDB:4YFL)], CH235-class antibody [CH235.12 (PDB:8SXJ)], DH1285 antibody (PDB:8U1D), HCDR3-dependent bnAbs [CH103 (PDB:9D7O), HJ16 (PDB:4YE4), VRC13 (PDB:4YDJ), and VRC16 (PDB:4YDK)], N276 glycan-dependent bnAbs [VRC40 (PDB:7LL1), VRC33 (PDB:7LL2), 179NC75 (PDB:7LLK), and CAP257-RH1 (PDB:5T33)], non-neutralizing antibodies [F105 (PDB:3HI1), b12 (PDB:2NY7), and b13 (PDB:3IDX)], and the CD4 receptor (PDB:5VN3).

IGHV gene	bnAb class	Antibody	Latitudinal angle (°)	Longitudinal angle (°)	HC-LC twist (°)	HC-LC latitudinal angle (°)
-	-	CD4	64.7	102.2	NA	NA
IGHV1-2*02	VRC01-class	VRC01	90.9	82.5	31.2	30.4
		PG04	91.1	87.3	18.4	26.1
		12A21	92.3	92.4	13.5	26.4
		N6	92.1	90.0	9.0	27.5
		3BNC117	86.8	81.7	28.7	31.6
		VRC08	89.0	79.7	34.1	29.9
	IOMA-class	IOMA	78.3	82.4	23.9	40.6
		ACS101	82.8	83.6	28.9	39.0
IGHV1-46*01	CH235-class	CH235.12	88.5	88.0	16.5	31.0
	1B2530-class	1B2530	86.3	85.6	18.9	36.9
	561-class (type I)	8ANC131	90.4	98.2	10.8	30.5
		8ANC134	91.6	98.2	14.6	29.6
	561-class (type I)	1-18	88.2	97.1	9.7	36.6
		1-55	87.4	96.5	10.7	37.6
	561-class (type II)	1-23	77.8	91.0	14.7	47.1
		9-71	78.1	90.8	14.8	47.1
IGHV4-59*01	H-CDR3-dependent	CH103	48.5	89.0	68.8	NA
IGHV3-30*01		HJ16	62.7	52.2	-16.3	NA
IGHV1-69*01		VRC13	43.9	87.2	5.6	NA
IGHV2-23*01		VRC16	55.6	91.2	79.7	NA
IGHV1-2*06	N276g-dependent	VRC40	72.9	71.7	49.1	58.5
IGHV3-21*01		VRC33	70.4	62.5	-127.8	NA
IGHV4-34*02		179NC75	68.5	61.6	119.2	NA
		CAP257-RH1	77.9	61.0	91.4	NA
	non-nAbs	F105	39.4	135.8	-137.6	NA
		b12	37.5	113.9	106.8	NA
		b13	26.6	122.2	122.8	NA
IGHV1-105*01	DH1285-class	DH1285	92.7	88.6	47.4	25.2

Table S8. CD4bs binding modes and N276 glycan interactions across CD4bs bnAb classes. Related to Figure 5.

Binding mode and N276 glycan angles upon binding by VRC01-class bnAbs [VRC01 (PDB:5FYJ), PG04 (PDB:3SE9), 12A21 (PDB:4JPW), 3BNC117 (PDB:5V8M), VRC08 (PDB:4XMP) and N6 (PDB:5TE6)], IOMA-class bnAbs [IOMA (PDB:5T3Z) and ACS101 (PDB:7Z3A)], 561-class type-I [8ANC131 (PDB:4RWY), 8ANC134 (PDB:4RX4), 1-18 (PDB:6UDJ), and 1-55 (PDB:6UDK)], 561-class type-II [1-23 and 9-71], 1B2530-class antibody [1B2530 (PDB:4YFL)], CH235-class antibody [CH235.12 (PDB:8SXJ)], N276 glycan-dependent bnAbs [VRC40 (PDB:7LL1), VRC33 (PDB:7LL2), 179NC75 (PDB:7LLK), and CAP257-RH1 (PDB:5T33)], and the CD4 receptor (PDB:5VN3) compared to the CD4bs-unbound structures [BG505 (PDB:5FYL) and JR-FL (PDB:5FUU)]. Missing values are represented by (NA, grey).

IGHV gene	bnAb class	Antibody	$N276_{CA}$ - COM_{LC} distance (Å)	HC-LC latitudinal angle (°)	N276 glycan latitudinal angles (°)	N276 glycan longitudinal angles (°)
IGHV1-2*02	VRC01-class	VRC01	23.3	30.4	26.1	35.9
		N6	22.8	27.5	NA	NA
		12A21	21.4	26.4	NA	NA
		PG04	21.7	26.1	NA	NA
		VRC08	23.3	29.9	NA	NA
		3BNC117	23.0	31.6	29.0	54.9
	IOMA-class	IOMA	26.5	40.6	48.2	50.4
		ACS101	25.3	39.0	40.0	32.4
IGHV1-46*01	CH235-class	CH235.12	23.7	31.0	NA	NA
	1B2530-class	1B2530	25.7	36.9	NA	NA
	561-class (type I)	8ANC131	23.9	30.5	NA	NA
		8ANC134	23.5	29.6	NA	NA
	561-class (type I)	1-18	25.1	36.6	40.3	87.4
		1-55	25.8	37.6	49.0	46.4
	561-class (type II)	1-23	28.0	47.1	73.9	60.6
		9-71	28.0	47.1	71.9	84.7
IGHV1-2*06	N276g-dependent	VRC40	31.1	58.5	95.5	57.9
		CAP257-RH1	NA	NA	76.2	85.8
IGHV4-34*02		179NC75	NA	NA	104.9	102.1
IGHV3-21*01		VRC33	NA	NA	95.1	94.7
-	-	CD4	NA	NA	102.6	63.0
-	-	BG505 unbound	NA	NA	85.5	81.4
		JR-FL unbound	NA	NA	83.8	69.1
